# A Continuous Flow Process for the Defluorosilylation of HFC-23 and HFO-1234yf

**DOI:** 10.1021/acs.orglett.4c03274

**Published:** 2024-10-01

**Authors:** Sarah L. Patrick, James A. Bull, Philip W. Miller, Mark R. Crimmin

**Affiliations:** Department of Chemistry, Molecular Sciences Research Hub, 82 Wood Lane, Shepherds Bush, London, W12 0BZ, U.K.

## Abstract

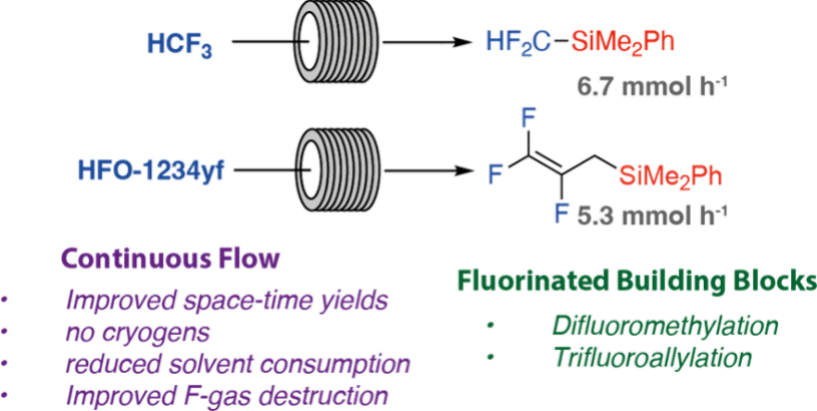

A continuous flow process has been developed for the defluorosilylation of trifluoromethane (HFC-23) and 2,3,3,3-tetrafluoropropene (HFO-1234yf) through reaction with lithium silanide reagents under inert conditions. Design of experiment optimization improved process conditions, including productivity, yields, reduction of solvent use, and gas destruction. The small chain fluorinated organosilane products R_3_SiCF_2_H and R_3_SiCH_2_C(F)=CF_2_ were competent nucleophiles in the fluoride-catalyzed difluoromethylation of aldehydes, and trifluoroallylation of aldehydes, ketones, and imines.

The use of hydrofluorocarbons (HFCs) and hydrofluoroolefins (HFOs) as fluorinated building blocks for synthetic chemistry is an attractive strategy.^1^ These fluorinated gases are produced on large scales due to their application as refrigerants, propellants, and blowing agents and, as such, are readily available. For example, HCF_3_ is produced on-scale as a byproduct of PTFE production, while HFO-1234yf is manufactured for use in air-conditioning units in the automotive sector. HFCs and HFOs typically contain between 1 and 4 carbon atoms and 1–6 fluorine atoms, meaning they are useful sources of small, fluorinated carbon chains, which are privileged fragments across the pharmaceutical, agrochemical, materials, and energy sectors.^2−6^ Moreover, there are established networks for recovery and purification of HFCs as they are controlled substances due to their high global warming potentials and known detrimental impacts on the environment. While HFOs have lower global warming potentials and are not currently recovered on scale, there is increasing concern over the fate of HFOs which contain CF_3_ groups as they breakdown into trifluoroacetic acid in the environment.^7,8^ There is also the potential that certain HFCs and HFOs may be classed as per- or polyfluorinated alkyl substances (PFAS) under newly proposed EU regulatlation.^9^

Recently, there has been a growth in the reports of synthetic transformations involving HFCs and HFOs as small chain fluorinated building blocks.^10−17^ Despite these advances, the scale-up of the new methodology continues to be challenging. HFCs and HFOs are often available in the form of low-pressure gas reservoirs. Scaling up reactions in batch is impractical due to the large volumes of gases involved and potential for extreme exotherms. Continuous flow methods offer key enabling technologies to solve these problems. Flow methods allow safe scale-up and process optimization, through efficient liquid–gas mixing, increased heat dissipation, and reduction of reactor volumes.^18−20^

One of the first continuous flow processes that used HFCs was reported by Grushin and co-workers in 2014.^21^ They demonstrated that “ligandless” [CuCF_3_] could be prepared from HCF_3_, CuCl, and KOtBu in DMF using Et_3_N**·**3HF as a stabilizing agent to slow degradation. [CuCF_3_] itself is a versatile trifluoromethylating agent that has been applied in a series of carbon–carbon bond-forming reactions.^22^ Since this breakthrough, HCF_3_ has been used as a trifluoromethylating agent under flow conditions in base-mediated (e.g., KOtBu, KHMDS) reactions with aldehydes and ketones,^23,24^ imines,^25^ and esters.^26^ Kappe and co-workers have reported the difluoromethylation of enolizable esters with HCF_3_ in the presence of a base.^27^ This methodology has been applied to a scalable continuous flow process for the synthesis of Eflornithine with production rates of 24 mmol h^–1^.^28^ A similar approach has been used for the difluoromethylation of enolizable nitriles, albeit with the design of a specialist reactor to control the temperature in flow.^29^ To the best of our knowledge, continuous flow methods have yet to be applied to the synthetic transformations of HFOs.

Over the past few years, we have reported several synthetic approaches for the defluorosilylation and defluoroborylation of HFCs and HFOs.^30−32^ This includes the defluorosilylation of industrially relevant HCF_3_ (HFC-23) and 2,3,3,3-tetrafluoropropene (HFO-1234yf) under batch conditions using lithium silanide reagents. In the case of HFC-23, the reaction proceeds efficiently over 10 min at 25 °C, provided a low concentration of **1·PMDETA** (20 mM) and excess of HCF_3_ (approximately 7 equiv) is used. In the case of HFO-1234yf, cryogenic temperatures of −78 °C and a reaction time of 3 h are required. Both reactions could be performed on a 1–3 mmol scale, allowing the isolation of products in 68–69% yield (Figure 1).

**Figure 1:**
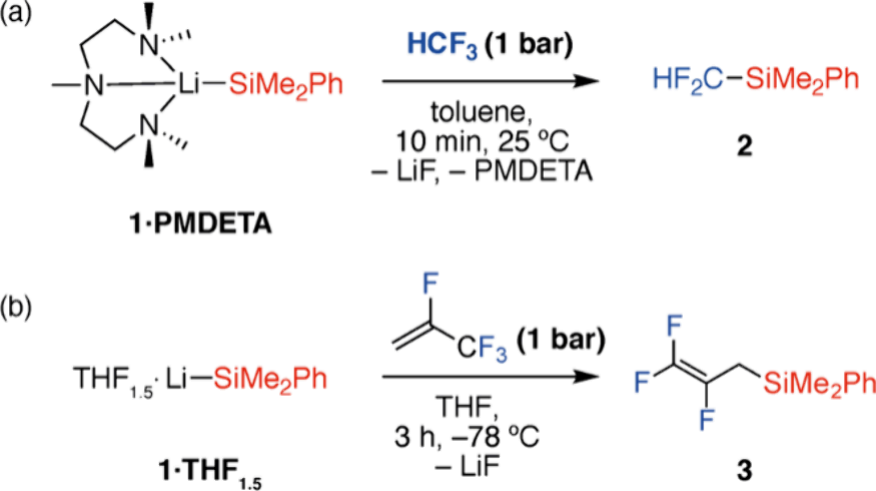
Defluorosilylation of (a) HFC-23 and (b) HFO-1234yf under batch conditions.

In this article, we describe a continuous flow method for defluorosilylation of both HFC-23 and HFO-1234yf. The approach allows for facile scale-up. Optimal process conditions result in improved production rates and reduced solvent use compared to batch conditions while also obviating the need for cryogenic temperatures. The new enabling technology provides access to small chain fluorinated building blocks in sufficient quantities to effectively explore their onward chemistry. We demonstrate a broad scope of difluoromethylation and trifluoroallylation.

The defluorosilylation of HCF_3_ with **1·PMDETA** was conducted in flow using a Vapourtec easy-scholar reactor system (Figure 2a). A reactor coil of length = 495 cm and volume = 3.9 mL was used, leading to residence times of between 30 s and 6 min depending on flow rates used. All reactions were conducted at 25 °C. Flow rates were regulated to initially produce a segmented flow regime directly after the T-junction; however, as the reaction proceeded, HCF_3_ was consumed, resulting in dissipation of the segmented flow. Samples for analysis were collected following dispensing of 1 to 2 reactor volumes under flow. Precipitation of a solid, presumed to be LiF, was observed during reactions but did not cause fouling of the reactor tubing. Formation of **2** was quantified by both ^19^F NMR spectroscopy and GC-FID analysis.

**Figure 2:**
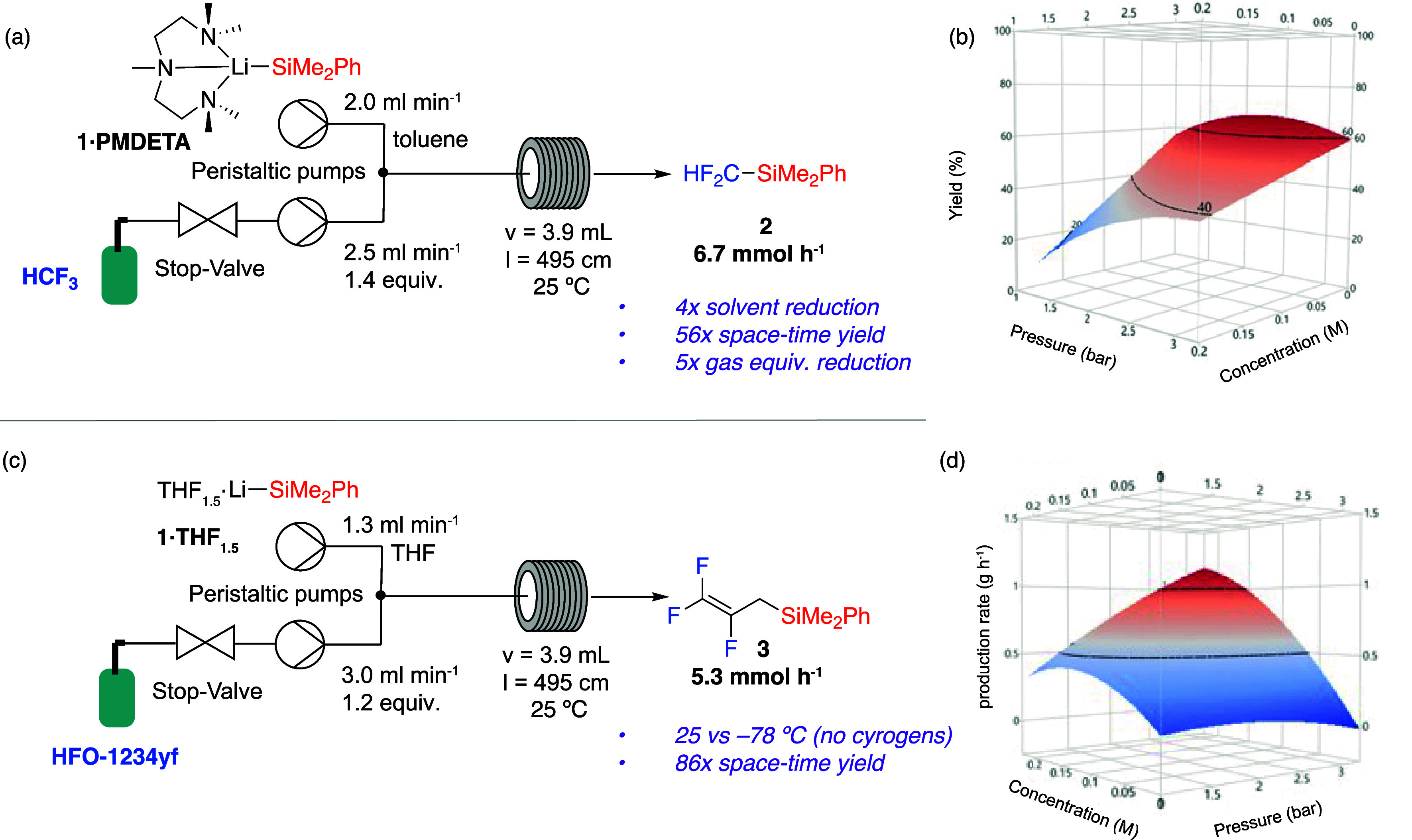
(a) Flow-setup for defluorosilylation of HCF_3_ with **1·PMDETA** and (b) DoE surface showing a yield response to changes in pressure and concentration of **1·PMDETA**. (c) Flow-setup for defluorosilylation of HFO-1234yf with **1·THF**_**1.5**_ and (d) DoE surface showing production rates response changes in pressure and concentration of **1·PMDETA**.

A design-of-experiment (DoE) strategy was used to optimize the process and gain a better understanding of which variables had the greatest statistical significance on the outcome. A definitive screen DOE was constructed by using 17 initial experiments, augmented by a further 10 experiments to extend the range of each variable beyond the initial conditions. The yield of **2** was measured across an average of three samples per run. All statistical analysis and modeling were performed using JMP software. Modeling of the resultant data revealed that concentration of **1·PMDETA** has the greatest statistical influence on yield, followed by gas pressure, then gas flow rate (Figure 2b).

Optimal conditions for maximizing the formation of product were found to be 1.2 mL min^–1^ liquid flow rate of a 20 mM solution of **1·PMDETA**, with 2.4 mL min^–1^ gas flow rate of HCF_3_ at 2.5 bar forming **2** in 74% yield, corresponding to a production rate of 1.1 mmol h^–1^. With a space-time yield of 0.27 mol L^–1^ h^–1^, this corresponds to a 10-fold increase from the previously reported batch conditions. A multiresponse model was then fit using productivity, solvent usage, and calculated gas destruction to accurately predict within experimental error the optimal process conditions. The optimal process conditions were found to be a 2.0 mL min^–1^ liquid flow rate of a 110 mM solution of **1·PMDETA** and 2.5 mL min^–1^ gas flow rate of HCF_3_ at 3.1 bar, forming **2** at a predicted 53% yield (Figure 2d). These conditions were tested on a 3 g scale to give a confirmed 51% in situ yield corresponding to a production rate of 6.7 mmol h^–1^, and a space-time yield of 1.5 mol L^–1^ h^–1^. Comparison of these optimal process conditions to those in batch reveals a dramatic reduction in solvent use (74 mL mmol^–1^ in batch; 18 mL mmol^–1^ in flow) and equivalents of HCF_3_ required (7 equiv. in batch; 1.4 equiv. in flow), with a 56-fold improvement in space-time yield (0.026 mol L^–1^ h^–1^ in batch; 1.5 mol L^–1^ h^–1^ in flow).

The same Vapourtec system can be adapted for the defluorosilylation of HFO-1234yf, forming **3** successfully at room temperature (Figure 2c). This is the first example of applying flow methods to upgrade HFOs through synthesis. Optimal process conditions were found to be a 1.3 mL min^–1^ liquid flow rate of a 110 mM solution of **1·THF**_**1.5**_, 3.0 mL min^–1^ gas flow rate of HCF_3_ at 1.4 bar, leading to 62% yield of **3** with a production rate of 5.3 mmol h^–1^. The reaction proceeds selectively at room temperature, with just 1.2 equiv of gas and a residence time of 2.5 min, compared to a 3 h reaction time in batch.

This reaction could also be effectively run for an extended period using crude **1·THF**_**1.5**_ to allow removal of a purification step from the overall procedure. The flow methodology offers a marked increase in space-time yield of 1.05 mol l^–1^ h^–1^, an 86-fold improvement compared to batch.

CF_2_HSiMe_3_ has previously been demonstrated to be a versatile difluoromethylation agent, reacting with a range of electrophiles in the presence of a fluoride source (e.g., CsF) or base (e.g., KOtBu).^33^ Due to its lower volatility, **2** might offer improved ease of handling and storage compared to CF_2_HSiMe_3_; however, its applications in synthesis are limited to difluoromethylation of a range of aromatic and aliphatic aldehydes catalyzed by KF.^34^ To further expand the utility of this compound, we investigated reactions with a series of aromatic, α,β-unsaturated, and heteroaromatic aldehydes. Reactions were run at 25 °C using CsF as a catalyst. Once conversion to the silylated alcohols was complete, TBAF was added as a stoichiometric reagent to effect the complete desilylation of the products. Difluoromethylated alcohols **4a**–**f** were prepared in modest to good yields (Figure 3). The reaction tolerated furyl, pyridyl, benzothiothenyl, and pyrazolyl groups along with halogens. An analogue of the key intermediate in the preparation of Telotristat Ethyl (a tryptophan hydroxylase inhibitor) in which the CF_3_ group has been substituted for a CF_2_H group could be prepared.^35^ Difluoromethane was observed as a minor byproduct in certain cases, most likely arising from the desilylation of **2** with CsF.

**Figure 3:**
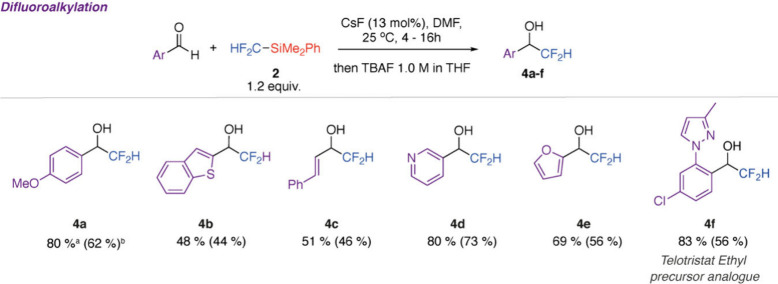
Reaction scope of difluoromethylation of aldehydes by **2**. ^a^Yields determined by ^19^F NMR spectroscopy as compared to that of trifluorotoluene as an internal standard. ^b^Isolated yields were obtained following purification by column chromatography.

The trifluoroallylsilyl reagent **3** was similarly an effective reagent for the trifluoroallylation of carbonyl compounds in the presence of catalytic TBAF yielding **5a**–**5m** (Figure 4). While allyl silanes are well-established allylating reagents, typically under Lewis acidic conditions, the use of fluorinated analogues is under-developed^36^—likely due to the availability of suitable reagents. Electron-deficient and electron-rich aldehydes, along with activated imines and ketones, were all successful electrophiles. Structures of **5a** and **5j** were determined by single crystal X-ray diffraction. 2,3,3-Trifluoroprop-1-ene was observed as a minor side-product in these reactions but is readily separated from the product.

**Figure 4:**
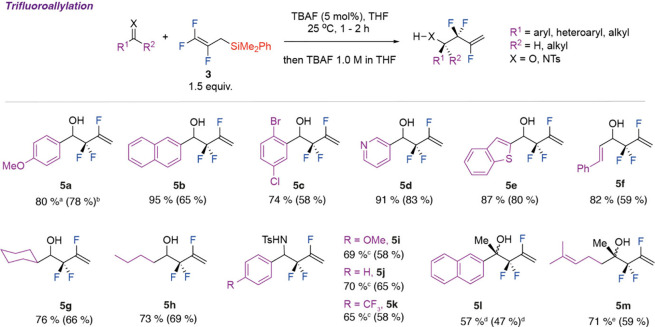
Reaction scope of the trifluoroallylation of aldehydes, ketones, and imines by **3**. ^a^Yields determined by ^19^F NMR spectroscopy as compared to trifluorotoluene as an internal standard. ^b^Isolated yields following purification by column chromatography. ^c^Reactions performed at 60 °C with 15 mol % portions of TBAF added every 2–3 h. ^d^Product was contaminated by 15% ketone starting material. ^e^Product contaminated by 8% ketone starting material.

In summary, we have developed a continuous flow process for the production of small chain fluorinated building blocks from trifluoromethane (HFC-23) and 2,3,3,3-tetrafluoropropene (HFO-1234yf). Flow conditions offer significant advantages over batch, including: (i) improved production rates, (ii) better control of exotherms (meaning cryogens can be avoided), and (iii) reductions in the volume of solvent and fluorinated gas required for efficient conversion. Accessing building blocks **2** and **3** on ∼10 mmol scale has enabled their further development as reagents to install C_1_ to C_3_ fluorinated motifs in organic molecules. This has been achieved through carbon–carbon bond formation via the difluoromethylation and trifluoroallylation, of carbonyl and imine functional groups.^37^

## Data Availability

The data underlying this study are available in the published article and its Supporting Information.

## Supporting Information Available

Synthetic procedures, flow setup and DoE, NMR spectra of all compounds, crystal structures of **5a**, **5j** (PDF)
