# Supplementary material for: A Continuous Flow Process for the Defluorosilylation of HFC-23 and HFO-1234yf
Source: Org Lett. 2024 Oct 1;26(40):8605–9. doi: 10.1021/acs.orglett.4c03274 (PMC11474948; doi:10.1021/acs.orglett.4c03274)
Supplement: Supplementary file 1 — ol4c03274_si_001.pdf [file ol4c03274_si_001.pdf]

A Continuous Flow Process for the Defluorosilylation of HFC-23 and HFO-1234yf

Supplementary Information

Sarah L. Patrick, James A. Bull,* Philip W. Miller,* Mark R. Crimmin*

*m.crimmin@imperial.ac.uk, Philip.miller@imperial.ac.uk, j.bull@imperial.ac.uk

Department of Chemistry, Molecular Sciences Research Hub, 82 Wood Lane, Shepherds Bush, London, W12 0BZ, UK.

Table of Contents

1. General Procedures: 3

2.1 Synthesis of lithium based nucleophiles, **1** 4

3.1 Reaction of HCF_3 with **1•PMDETA** in continuous flow 6

3.2 Design of experiment: Definitive screen design 11

4.1 Reaction of HFO-1234yf with **1•THF** in continuous flow 20

5.1 Difluoromethylation Reactions 22

6.1 Allylation reactions 27

7.1 X-ray data for compounds **5a**, and **5j**. 39

8.1 NMR spectra 41

9. References 77

1. General Procedures:

Unless otherwise specified, standard Schlenk line and glovebox techniques were used for all manipulations under an inert atmosphere of nitrogen or argon. NMR scale reactions were performed in J. Young NMR tubes equipped with internal standard capillaries of ferrocene (^{1}H NMR spectroscopy), internal standard of α,α,α-trifluorotoluene or 1,2-difluorobenzene was used (^{19}F NMR spectroscopy), and prepared in a glovebox. An MBraun Labmaster glovebox was used, operating at <0.1 ppm and H_2O and <0.1 ppm O_2.

^{1}H, ^{13}C, ^{7}Li and ^{19}F NMR spectra were recorded on BRUKER 400 MHz or 500 MHz machines, and referenced against $Si(CH_3)_4$ (^{1}H and ^{13}C), $CFCl_3$ (^{19}F) or LiCl (^{7}Li). A 55 s delay was used for quantitative ^{19}F NMR integration. Data was processed using the MestReNova software package. The reported values for ^{1}H NMR data are as follows: chemical shifts (δ ppm), multiplicity (where s = singlet, d = doublet, dd = doublet of doublets, and m = multiplet), integration and coupling constant, J (Hz).

Solvents were dried over activated alumina from a solvent purification system (SPS) based upon the Grubbs design and degassed before use. Glassware was dried for >6 h prior to use at 120 °C. Benzene-d_6, toluene, diethyl ether, pentane, hexane, and THF were de-gassed and stored over 3 Å molecular sieves before use. All reagents were acquired from Sigma Aldrich (Merck), Tokyo Chemical Industry, Scientific Laboratory Supplies, or Fluorochem and used without further purification unless specified. All aldehydes were distilled *via* vacuum distillation at 0.1 mbar prior to use. Trifluoromethane (HCF_3) and HFO-1234yf were acquired from CK special gases and as a donation from Apollo Scientific and used without further purification or drying. Where liquids at 25 °C, reagents were dried over activated 3 Å molecular sieves and freeze-pump-thaw degassed prior to use. N,N,N′,N″,N″-pentamethyldiethylenetriamine (PMDETA) were distilled over CaH_2 and dried over activated 3 Å molecular sieves and freeze-pump-thaw degassed prior to use. IPrCuCl[1] and IPrCuO^{t}Bu[2] were prepared according to literature procedures.

Purifications were carried out by column chromatography on silica gel (tech grades, 60 Å, 230-400 mesh, 40-63 µm particle size) or by automated silica chromatography using a Biotage Selekt with Biotage Sfär Silica D Duo 60 µm 5 g or 10 g cartridges.

Quantitative gas chromatography (GC) analyses were performed with an Agilent Technologies 7820A GC with FID detector using an Agilent DB-WAX column. The carrier gas used was helium. Durene was used as internal standard.

AT-IR spectra were recorded on an Agilent Technologies Cary 630 FTIR spectrometer. The high-resolution mass spectrometry (HRMS) analyses were performed using electrospray ion source (ESI). ESI was performed using a Waters LCT Premier (ES-TOF) equipped with an ESI source operated in positive or negative ion mode.

2.1 Synthesis of lithium based nucleophiles, 1

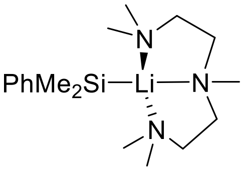

Synthesis of 1•PMDETA: Lithium metal cut into ~0.2 cm pieces (606 mg, 87.3 mmol) was washed with *n*-hexane, and added to a Schlenk flask and dried under vacuum for 30 mins. Under a positive pressure of argon, the lithium pieces were suspended in THF (80 mL) and dimethylphenylsilane chloride (6.00 mL, 35.2 mmol) was added at 0 °C (ice bath). The reaction mixture was allowed to warm to room temperature and stirred for 16 h, while the solution turned dark red. The solvent was removed *in vacuo,* then under positive flow of argon toluene (80 mL) was added. The reaction mixture was filtered *via* cannulation and the filtrate was concentrated *in vacuo* to half volume. PMDETA (8.8 mL, 42.2 mmol) was added at room temperature and the reaction mixture was stirred for 1 h. Under a positive flow of argon, *n*-hexane (60 mL) was added and the resulting solution was placed in a freezer (-35 °C) overnight affording brown crystals (5.89 g, 18.7 mmol, 53%).

NMR data are consistent with literature.[3]

^{1}H NMR (400 MHz, C_6D_6) δ/ppm: 7.95 (d, 2H, $^{3}J_{HH}$ = 6.8 Hz, *o*-C**H$_{Ph}$**), 7.44 (dd, 2H, $^{3}J_{HH}$ = 7.3 Hz, $^{3}J_{HH}$ = 6.8 Hz, *m*-C**H$_{Ph}$**), 7.19 (t, 1H, $^{3}J_{HH}$ = 7.3 Hz, *p*-C**H$_{Ph}$**), 1.90 (s, 3H, N(C**H**$_3$)), 1.83 (br s, 12H, (N(C**H**$_3$)$_2$), 1.69-1.40 (br m, 8H, (N(CH)$_2$)$_4$), 0.87 (s, 6H, Si(C**H**$_3$)$_2$).

^{13}C NMR (100 MHz, C_6D_6) δ/ppm: 165.2 (s, 1C, i-**C**^{IV}Si), 133.9 (s, 2C, *o*-**C**H$_{Ph}$), 127.0 (s, 2C, *m*-**C**H$_{Ph}$), 123.3 (s, 1C, *p*-**C**H$_{Ph}$), 57.0 (s, 2C, N**C**H$_2$), 53.4 (s, 2C, N**C**H$_2$), 45.9 (br s, 4C, N(**C**H$_3$)$_2$), 44.8 (s, 1C, N(**C**H$_3$)), 7.7 (s, 2C, Si(**C**H$_3$)$_2$).

^{7}Li NMR (194 MHz, C_6D_6) δ/ppm: 1.43 (s).

$PhMe_2Si{-}Li{-}THF_{1.5}$ **Synthesis of 1·THF$_{1.5}$:** Lithium (326 mg, 47.0 mmol), washed with hexane, was added to a Schlenk flask and dried under vacuum for 30 mins. Under a positive pressure of argon THF was added (40 mL) and reaction was cooled to 0°C (ice-bath), dimethylphenylsilane chloride (3.00 mL, 17.6 mmol) was added. The reaction mixture was allowed to warm to room temperature and stirred for 16 h, on return a dark red solution was observed. The solvent was removed *in vacuo* and under positive flow of argon toluene (40 mL) was added. The reaction mixture was filtered via cannulation and the filtrate was concentrated *in vacuo*, a dark red oil was obtained (3.49 g, 10.8 mmol, 61%). (The number of THF molecules varied between batches from 1-2.5 molecules).

NMR consistent with literature.[4]

^{1}H NMR (400 MHz, C_6D_6, 298 K) δ/ppm: 7.80 (d, 2H, $^3J_{HH}$ = 7.1 Hz, *o*-C**H**), 7.33 (t, 2H, $^3J_{HH}$ = 7.65 Hz *m*-C**H**), 7.13 (t, 1H, $^3J_{HH}$ = 7.1 Hz, *p*-C**H**), 3.36 (m, 6H, (C**H**$_2$)$_2$O), 1.23 (m, 6H, (C**H**$_2$)$_2$CH$_2$O), 0.72 (s, 6H, Si(C**H**$_3$)$_2$).

^{13}C NMR (100 MHz, C_6D_6, 298 K) δ/ppm: 159.6 (s, 1C, **C**^{IV}Si), 133.8 (s, 2C, *o*-**C**H), 128.1 (s, 2C, *m*-**C**H), 124.9 (s, 1C, *p*-**C**H), 68.6 (s, 3C, (**C**H$_2$)$_2$O), 25.5 (s, 3C, (**C**H$_2$)$_2$CH$_2$O), 6.0 (s, 1C, Si(**C**H$_3$)$_2$).

^{7}Li NMR (194 MHz, C_6D_6) δ/ppm: 1.38 (s).

3.1 Reaction of HCF_3 with 1•PMDETA in continuous flow

General procedure:

Flow reactions were performed using Vapourtec easy-Scholar Peristaltic Pumps. Reagents and solvents were loaded under argon with a 19 gauge stainless steel luer lock needle (1.07 mm OD). 1/16'' OD FEP tubing was used (ID = 1 mm) and a T-junction (P-714) was used as a micromixer. The gas was delivered from a lecture bottle of 36 bar regulated to a range of 1.0-3.2 bar and passed through a peristaltic pump to control the flowrate, a Swagelok micro-needle valve (SS-SS1) was used as a stop valve. The residence time T_R was measured during the reaction using a stopwatch. Prior to the reaction, the channels were primed with anhydrous toluene or THF and HCF_3 for 10 mins.

Procedure with optimum process conditions: After being primed with dry toluene, **1•PMDETA** (0.11 M) was delivered through peristaltic pump A, set to a flowrate of 2.0 mL/min. HCF_3 (3.1 bar) was delivered through peristaltic pump B, set to a flowrate of 2.5 mL/min and mixed at a T junction to give segmented flow through the coil reactor (ID = 1 mm, length 495 cm, 3.9 mL reactor volume, T_R = 1 min). The crude mixture was collected and purified as described below. Using these conditions, a production rate of 6.7 mmol/h (1.3 g/h, 51% *in situ* yield) and a gas consumption of 36% were calculated. Samples for *in situ* ^{19}F NMR analysis were collected after 1-2 reactor volumes. For each experiment 3 samples were taken for yield analysis. The system was constructed as described in the figure S9 below. ***Note:*** A back pressure regulator of 20 PSI can be used with the system with concentrations of 200 mM or lower. This causes higher solubility of the gas so the flow is no longer segmented but does not increase the yield significantly, any increase observed was still within ±5% error. Higher concentrations caused clogging of the BPR. Without the BPR higher concentration experiments did not cause fouling of the system when passing through up to 30 mL of solution. Higher volumes than this were not tested with 0.2 M but a build-up of residue within the tubing was observed, sonication can be used to displace residue build-up.

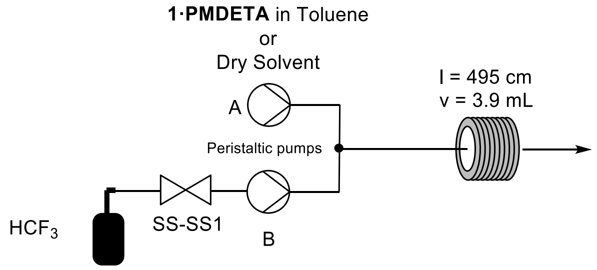

Figure S1: General Schematic for flow reaction.

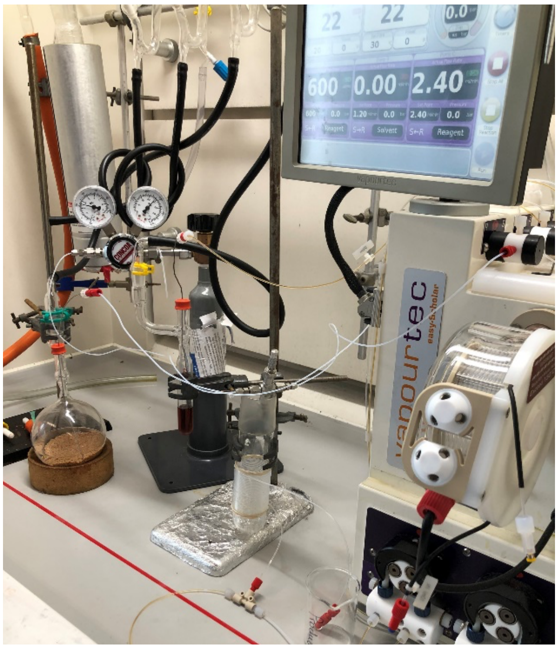

Figure S2: Photo of general setup.

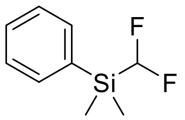

Purification of (difluoromethyl)dimethylphenylsilane 2: Solvent was removed *in vacuo*, the crude sample was separated between 2 M HCl(aq) (2 x 20 mL) and *n*-hexane (2 x 25 mL) then washed with water. The combined organics were dried over anhydrous magnesium sulfate and filtered; the solvent was removed *in vacuo*. The crude oil was purified by vacuum distillation at 40-60 °C (oil bath) at 0.1-0.5 mbar to obtain a colourless oil, (660 mg, 3.54 mmol, 39%) giving a space-time yield of 1.48 mol/L/h.

NMR data is consistent with literature.[3]

^{1}H NMR (400 MHz, C_6D_6, 298 K) δ/ppm: 7.38 (dd, 2H, $^{3}J_{HH}$ = 7.5 Hz, $^{4}J_{HH}$ = 2.0 Hz, *o*-C**H**$_{\mathbf{Ph}}$), 7.17-7.11 (m, 3H, *m,p*-C**H**$_{\mathbf{Ph}}$), 5.69 (t, 1H, $^{2}J_{HF}$ = 46.2 Hz, CF_2**H**), 0.18 (s, 6H, Si(C**H**$_3$)$_2$).

^{13}C NMR (100 MHz, C_6D_6, 298K) δ/ppm: 134.5 (s, 2C, *o*-**C**H$_{Ph}$), 132.8 (m, 1C, **C**$^{IV}{}_{Ph}$), 130.5 (s, 1C, *p*-**C**H$_{Ph}$), 128.4 (s, 2C, *m*-**C**H$_{Ph}$), 123.6 (t, 1C, $^{1}J_{CF}$ = 255.0 Hz, **C**HF_2), -7.0 (s, 2C, Si(**C**H_3)$_2$).

^{19}F NMR (376 MHz, C_6D_6, 298 K) δ/ppm: -137.7 (d, 2F, $^{2}J_{FH}$ = 46.2 Hz, C**F**$_2$H).

IR (neat)/cm^{-1}: 3074, 2966, 2906, 2118, 1488, 1428, 1319, 1253, 1115, 983, 790, 736, 700, 680, 638, 628, 538, 466.

In situ yield calculations

In situ yields were calculated using ^{19}F NMR spectroscopy, the desired product was integrated with respect to α,α,α-trifluorotoluene as shown in the spectra below. This example gave 65% yield.

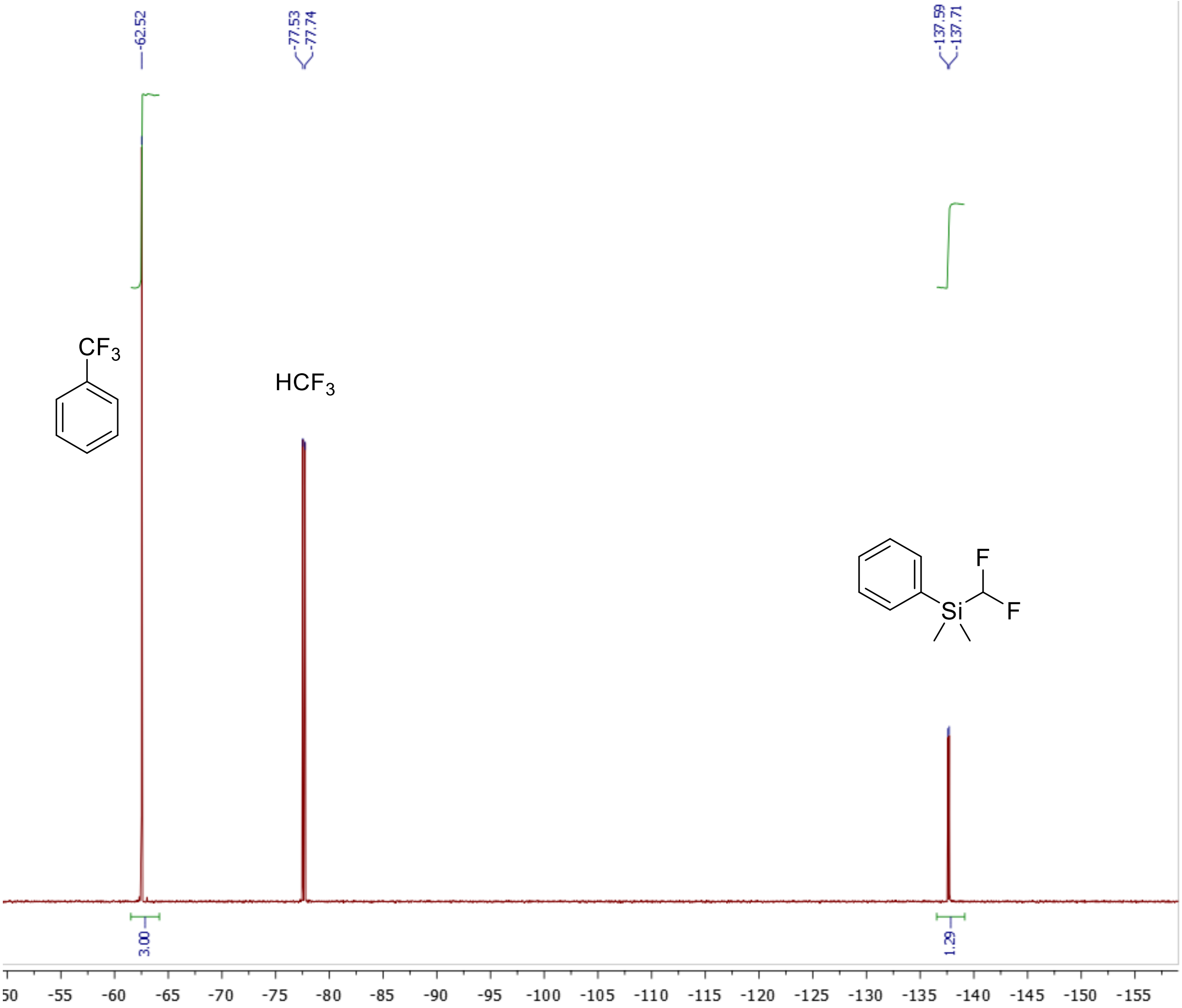

Figure S3: Example ^{19}F Spectra for yield calculation

Some yields from the DoE experiments were confirmed using Gas Chromatography using durene as a standard. A calibration curve was created as shown below by varying pure product and durene ratios.

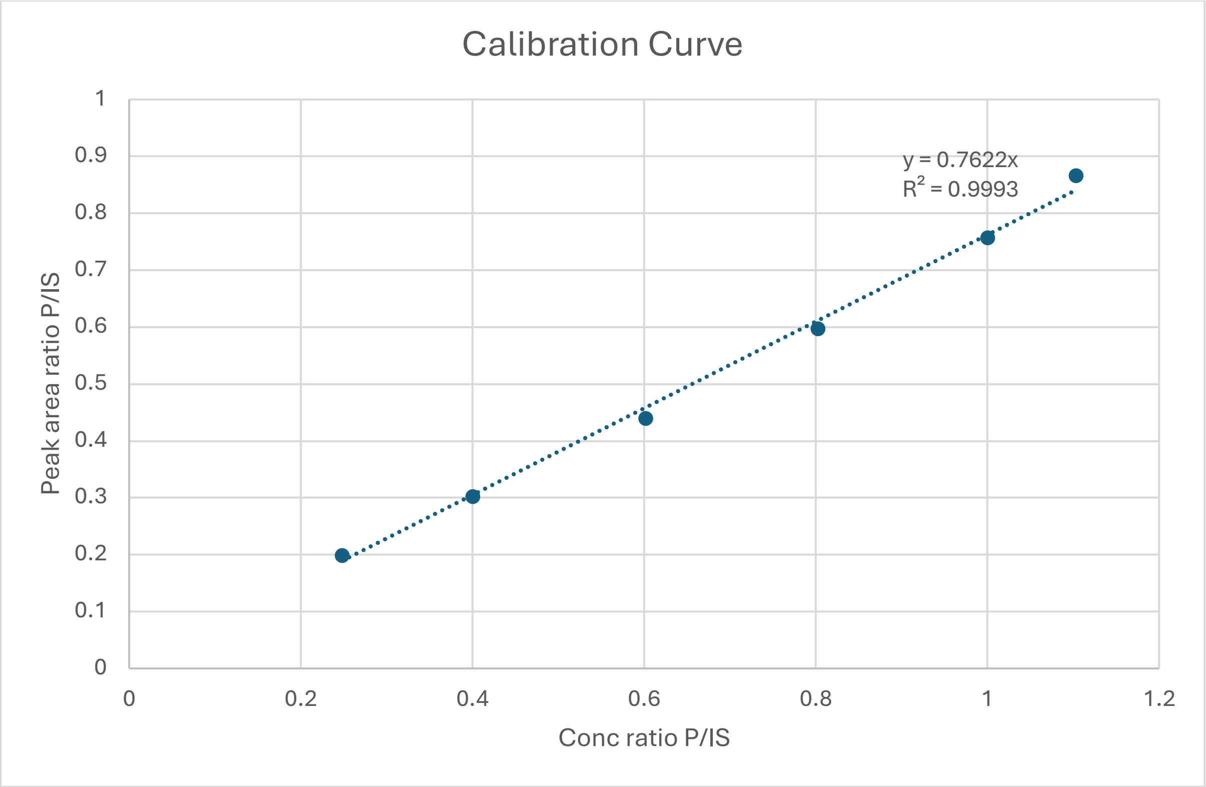

Figure S4: Calibration curve for GC analysis

Using the calibration equation the yields could be calculated directly from the peak ratios of the crude reaction samples.

An example trace is shown below, this confirmed the 65% yield shown above. Generally GC and NMR were in agreement of ±5% yield.

Method Info : start at 50 C for 1 min, ramp 4 C/min to 100, then 20 C/min to 270, no hold

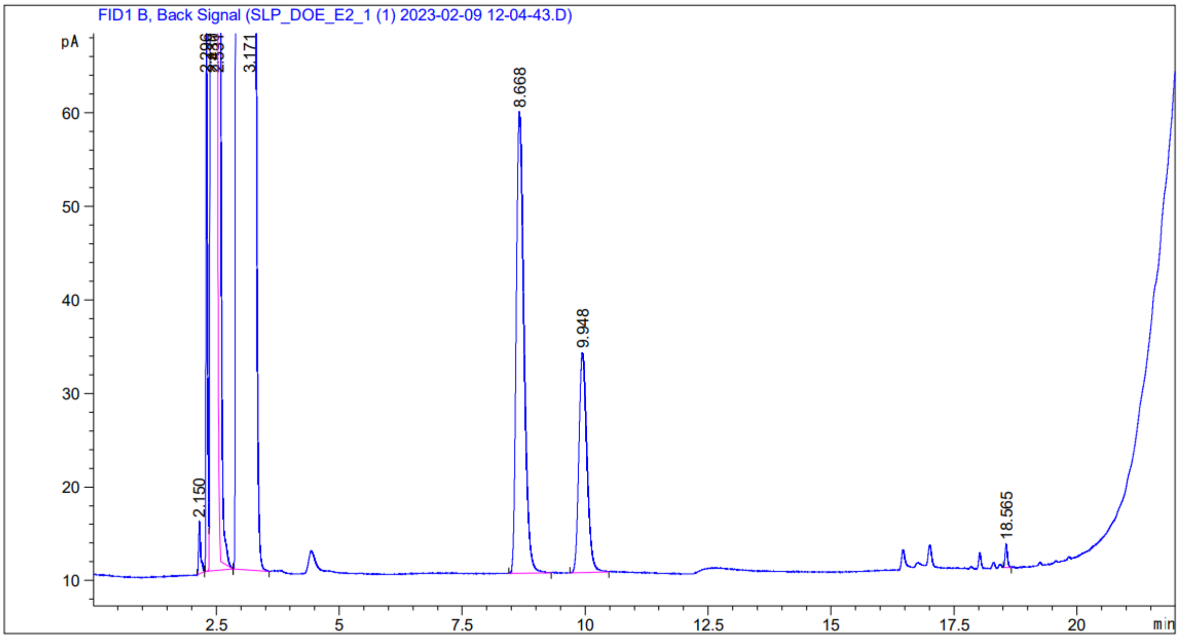

Peak #	RetTime [min]	Type	Width [min]	Area [pA*s]	Height [pA]	Area %
1	2.150	BB	0.0382	16.07269	5.62095	0.00345
2	2.296	BV E	0.0326	125.42736	57.59847	0.02695
3	2.430	BB R	0.0445	8.62068e4	2.88190e4	18.52611
4	2.531	VB E	0.0520	898.58777	247.24106	0.19311
5	3.171	BB	0.1134	3.77266e5	4.14608e4	81.07570
6	8.668	BB	0.1366	536.93634	49.32294	0.11539
7	9.948	BB	0.1543	267.61453	23.52118	0.05751
8	18.565	BB	0.0523	8.23659	2.48115	0.00177

Figure S5: Example GC trace

3.2 Design of experiment: Definitive screen design

Design of experiment (DoE) was used to optimise reaction conditions and gain greater understanding of which factors affected the yield. Screening designs can offer insight into which factors have the most statistical significance on the outcome of the reaction. While augmenting the initial screen with more experiments can allow for a response surface to be plotted with respect to the important factors. A predictive model of the set reaction space can be made using the experimental data. JMP Pro 16 software was used to generate the designs as well as statistically analyse the experimental data.

- **Screening**

A definitive screen design was chosen as an initial screen, 4 factors were chosen which are summarised in the table S7 below. The response was set as yield with the desirability set to maximise.

Factor	Type	Range
Liquid Flowrate	Continuous	0.6 – 1.2 mL/min
Gas Flowrate	Continuous	1 – 2.4 mL/min
Inlet Gas Pressure	Continuous	1.2 – 2.5 bar
Concentration	Continuous	0.02 – 0.2 M

Table S1: Summary of Factors used in definitive screen

A 17-experiment design was created including centre-points for all factors. Each experiment used 10 mL of **1•PMDETA** solution at the required concentrations to ensure steady state was reached before yield samples were taken. Yields were input as the average of three samples taken per run. The conditions which gave the optimum yield are highlighted, experiment 3 was repeated to give an average of 74% a second time. These conditions give a calculated production rate of 1.1 mmol/h and a space time yield of 0.27 mol/L/h. Standard deviation is also appended onto the data table, this was used as an indicator of which experiments needed repeating. The confirmed results are shown in the table S8 below.

Exp. No.	Liquid flowrate mL/min	Gas flowrate mL/min	Concentration M	Pressure bar	Yield %	Standard deviation
**1**	0.6	1	0.02	2.5	57	0.82
**2**	0.6	2.4	0.02	2.5	64	0.00
**3**	**1.2**	**2.4**	0.02	**2.5**	**74**	1.63
**4**	0.6	1	0.2	2.5	33	2.05
**5**	0.9	2.4	0.2	2.5	35	2.16
**6**	1.2	1.7	0.2	2.5	31	0.94
**7**	1.2	1	0.11	2.5	36	0.82
**8**	0.9	1.7	0.11	1.85	44	1.41
**9**	1.2	1	0.02	1.85	57	2.16
**10**	0.6	2.4	0.2	1.85	35	0.82
**11**	0.6	1.7	0.02	1.2	57	2.16
**12**	0.9	1	0.02	1.2	55	2.16
**13**	1.2	2.4	0.02	1.2	64	2.05
**14**	1.2	1	0.2	1.2	0	0.00
**15**	1.2	2.4	0.2	1.2	10	1.25
**16**	0.6	2.4	0.11	1.2	44	0.82
**17**	0.6	1	0.2	1.2	11	0.47

Table S2: 17 experiment definitive screen design

A two-level screening model was used to analyse the data, the fit of the model is best summarised using the actual vs predicted plot below. The R^2 is 0.97 with a root mean square error of 4.5. (Note – a definitive screening model was fit initially but this was not able to obtain important interaction data.)

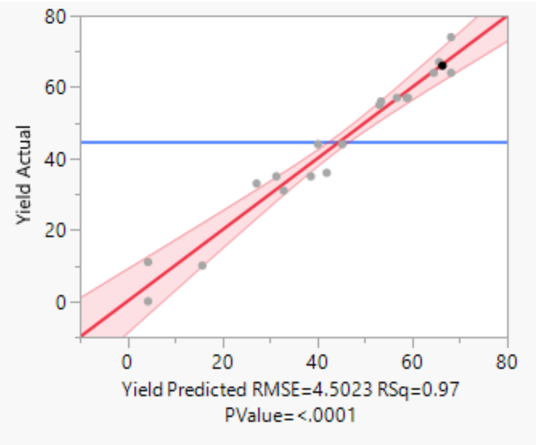

Figure S6: actual vs predicted initial screen model with average yields

The model was able to establish the factors that are statistically significant on the outcome of the yield. This is summarised below, the blue line indicates the threshold of significance that the model has set. Concentration, pressure, and gas flowrate are shown as important main effects and concentration*pressure is identified as an important secondary interaction. Pressure*Pressure is included to improve the RMSE and R^2 of the model, though this is below the threshold of significance.

Source	LogWorth	PValue
Concentration	9.882	0.00000
Pressure	4.355	0.00004
Gas flowrate	3.719	0.00019
Concentration*Pressure	2.801	0.00158
Pressure*Pressure	1.660	0.02188

Table S3: Table of factor importance

Analysing the residual plots allows for a visual representation of the factors impact on the yield. The steepness of the gradient shows greater importance. For example, concentration has the greatest impact on the yield and has a negative gradient therefore higher concentrations decrease the yield significantly.

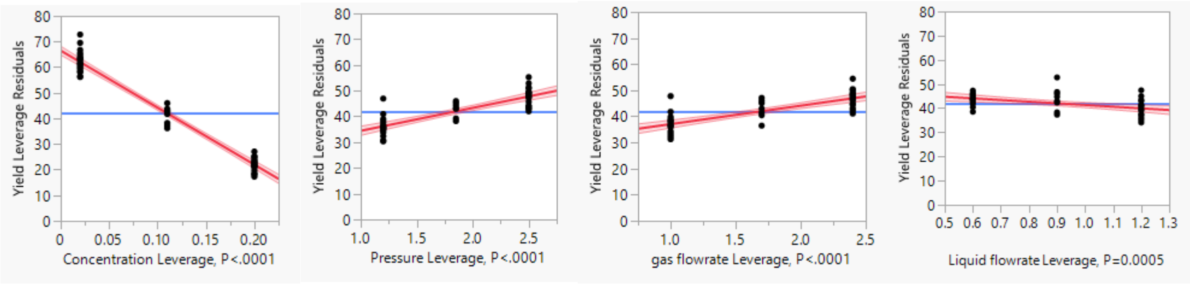

Figure S7: Main effect residual plots

JMP prediction profiler platform allows for the visualisation of the predicted yields based on the experimental data already input. Desirability can be set to specific ranges, here it is set so that any conditions which give below 40% yield are not desirable and the desirability then increasing linearly to 100%. The red dashed line shows the maximum desirability, this shows that the maximum yield is generally at the min or max of the ranges we have set for each factor. Therefore, it could be extrapolated that extending past these factor ranges could improve the yield further.

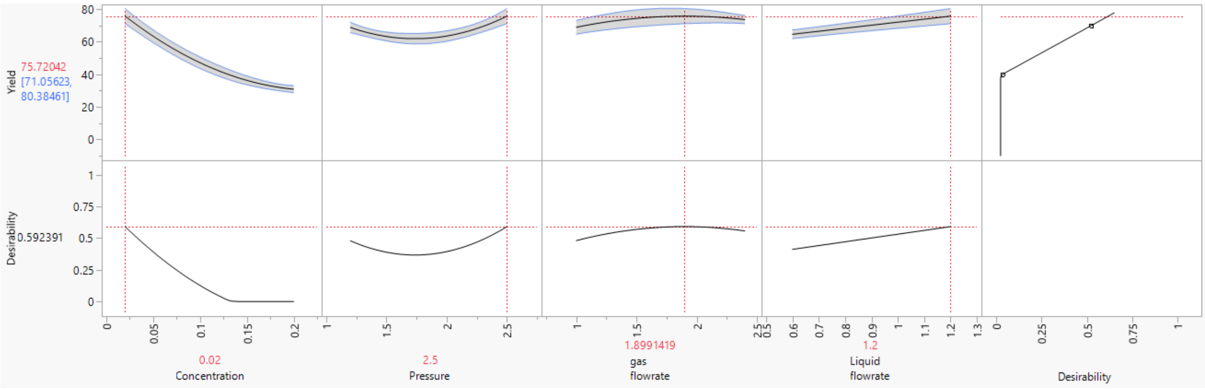

Figure S8: Prediction profiler of initial screen

Consequently, an augmented design was performed to extend the ranges of each factor with the aim to narrow in on the maximum yield conditions.

- **Augmented design**

An augmented design is used to add more information to the original screen, making the models predictions more accurate as well as allowing a higher confidence of the response surface created. The factor ranges are summarised below, while these were extended from the original screen, we were mindful not to move too far away from the original conditions as this would require a separate DoE to be performed rather than simply augmenting the original screen.

Factor	Type	Range
Liquid Flowrate	Continuous	1.0 – 2.0 mL/min
Gas Flowrate	Continuous	1.7 – 3.5 mL/min
Inlet Gas Pressure	Continuous	1.2 – 3.2 bar
Concentration	Continuous	0.01 – 0.1 M

Table S4: Summary of factors used in augmented design

The augmented design consists of 10 extra experiments using the new ranges, blocking was introduced here to indicate these experiments were ran at a separate time to the original screen. Block number was not found to be statistically significant confirming the reproducibility of the system and data.

**Exp. no**	**Liquid flowrate mL/min**	**Gas flowrate mL/min**	**Concentration M**	**Pressure bar**	**Block**	**Yield %**
**19**	1	1.7	0.055	2	2	57
**20**	**2**	**3.5**	**0.01**	**3.2**	**2**	**66**
**21**	1	3.5	0.1	3.2	2	51
**22**	2	1.7	0.01	1.2	2	35
**23**	2	1.7	0.1	3.2	2	44
**24**	2	3.5	0.055	2.2	2	56
**25**	2	3.5	0.055	1.2	2	48
**26**	1	3.5	0.01	1.4	2	60
**27**	1	1.7	0.1	1.85	2	45
**28**	1	1.7	0.01	3.2	2	54

Table S5: Augmented design experiment data table

A model was fit to this augmented design using the average yields shown in the table, this gave a RMSE of 4.5 and a R^2 of 0.96. Interestingly no conditions gave a higher yield than seen in the original screen indicating the optimum yield is within the ranges set. This is best visualised using the prediction profiler of the augmented model, generally a peak can be seen across the factors. The red dashed line indicates the predicted optimum conditions.

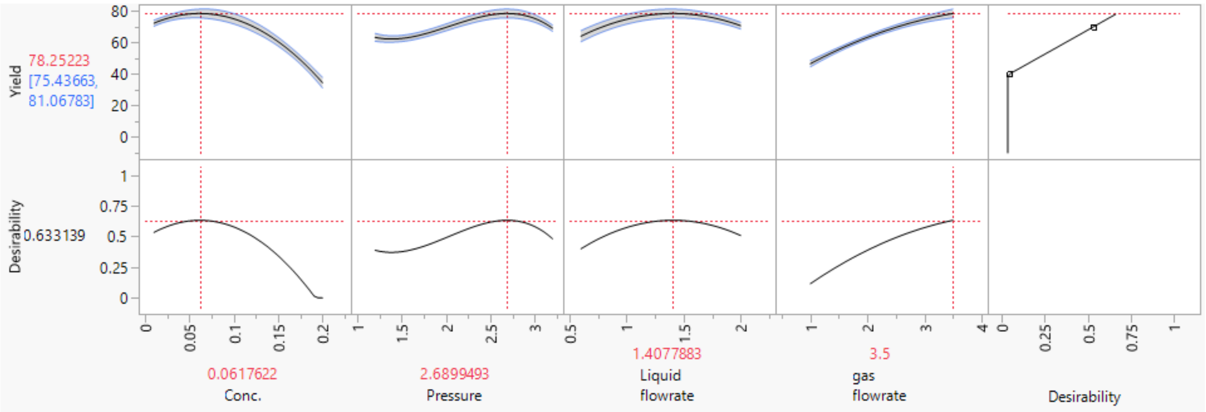

Figure S9: Prediction profiler for augmented design

After the augmented design was completed the optimum conditions from the original screen (1.2 mL/min of 0.02 M solution and 2.4 mL/min gas flowrate at 2.5 bar) were repeated to give 64%. This indicates that the standard error of 4.5 is representative of the experimental error.

The augmented design did improve the accuracy of the prediction formula, evidence by the table S12 below in which the actual average yields for the augmented design are shown next to the predictions before augmentation and after.

Exp. No.	Actual Yield	Initial Predicted	Augmented Predicted
**19**	57	54.8	56.1
**20**	66	76.6	66.4
**21**	51	68.0	49.3
**22**	35	64.5	41.3
**23**	44	55.1	43.1
**24**	56	68.4	60.2
**25**	48	64.6	43.6
**26**	60	77.3	64.3
**27**	45	44.2	46.0
**28**	54	63.7	55.9

Table S6: Initial predictions vs augmented predictions

Using the augmented model, response surfaces can be drawn with respect to the different factors such as concentration and pressure below. This is another way of plotting the optimum conditions required with respect to yield, similar to the profiler above. The surface is plotted along with residual points and contour lines displayed on each surface. The same can be plotted for other factors.

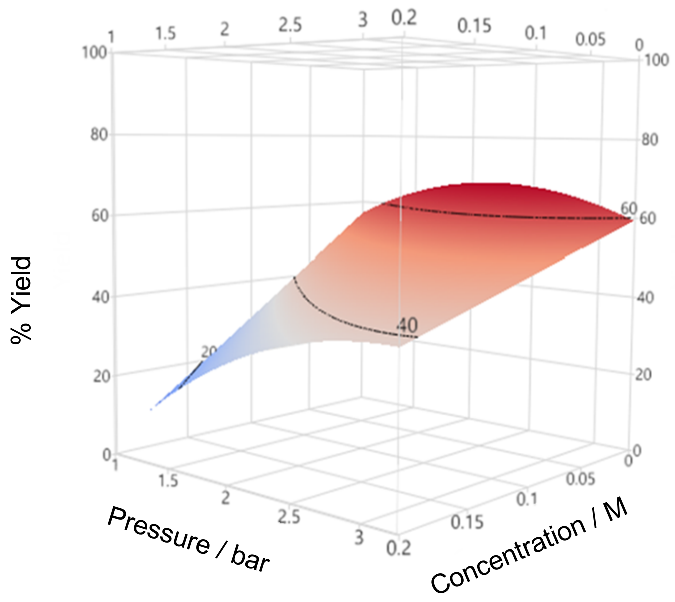

Figure S10: Surface plots of yield vs concentration vs pressure

- **Processability analysis**

The aim of earlier analysis was all focused on obtaining the optimum conditions with respect to yield as a response. However, a separate aim for this system is to find conditions that make this process scalable. Consequently, other criteria needed to be calculated for the existing experimental data. Gas consumption (using ideal gas approximation), production rate (g/h) and solvent required (mL/mmol) were selected as responses alongside yield. The data table below was used to generate the model, gas equivalents and gas consumption were calculated using ideal gas law. The other responses were calculated through unit manipulations. The production rate assumes that the flowrate of product is equal to the liquid flowrate, which we have generally observed experimentally as an underestimate.

Exp. No.	Yield %	Gas equiv.	Production rate (g/h)	Solvent needed (mL/mmol)	Gas destruction %
**7**	36	0.75	0.531	25.3	48.0
**6**	31	0.7	0.832	16.1	44.3
**4**	33	0.8	0.443	15.2	41.3
**16**	44	1.1	0.984	22.7	40.0
**27**	45	1.3	0.503	22.2	34.6
**8**	44	1.3	0.487	20.7	33.8
**25**	48	1.5	0.590	37.9	32.0
**17**	11	0.4	0.148	45.5	27.5
**5**	35	1.3	0.704	14.3	26.9
**23**	44	1.7	0.325	20.7	25.9
**10**	35	1.5	0.469	14.3	23.3
**19**	57	2.5	0.350	31.9	22.8
**12**	55	2.7	0.111	90.9	20.4
**24**	56	2.8	0.688	32.5	20.0
**15**	10	0.5	0.268	50.0	20.0
**9**	57	3	0.153	87.7	19.0
**18**	56	3.6	0.413	16.2	15.6
**13**	64	4.8	0.172	78.1	13.3
**21**	51	4.5	0.570	19.6	11.3
**22**	35	4.1	0.078	285.7	8.5
**11**	57	6.8	0.076	87.7	8.4
**3**	74	10	0.198	67.6	7.4
**1**	57	8	0.076	87.7	7.1

Exp. No.	Yield %	Gas equiv.	Production rate (g/h)	Solvent needed (mL/mmol)	Gas destruction %
**2**	64	20	0.086	78.1	3.2
**26**	60	19.7	0.067	166.7	3.0
**20**	66	23	0.148	151.5	2.9
**28**	54	22	0.060	185.2	2.5
**14**	0	0.2	0.000	0	0

Table S7: Data table with processability criteria, ordered by gas destruction.

JMP was used to fit a multi-response model using the original factors, this was fit using the augmented average yield data table. From this new response surfaces could be obtained with respect to the new response criteria. The ideal conditions for each response are different but the prediction profiler was able to combine the responses by setting user-specified desirability functions.

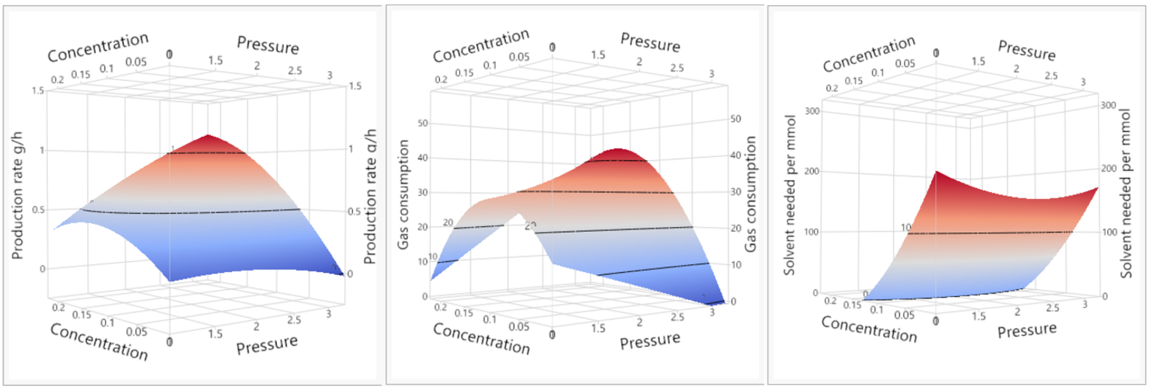

Figure S11: Surface plots for Production rate, gas consumption and solvent requirement all with respect to concentration and pressure

The desirability functions for each response are set on a scale of 0-1 for example yield was set as 40% = 0 and 100% = 1. Production rate and gas consumption were set as equally important at 1 while yield and solvent needed was set as less important at 0.5. An extrapolation control was applied to predict an optimum condition within what had been tested already to allow for greater confidence in the predictions. Using the “optimise for maximum desirability” function within the platform the conditions shown as the red-dashed line were obtained.

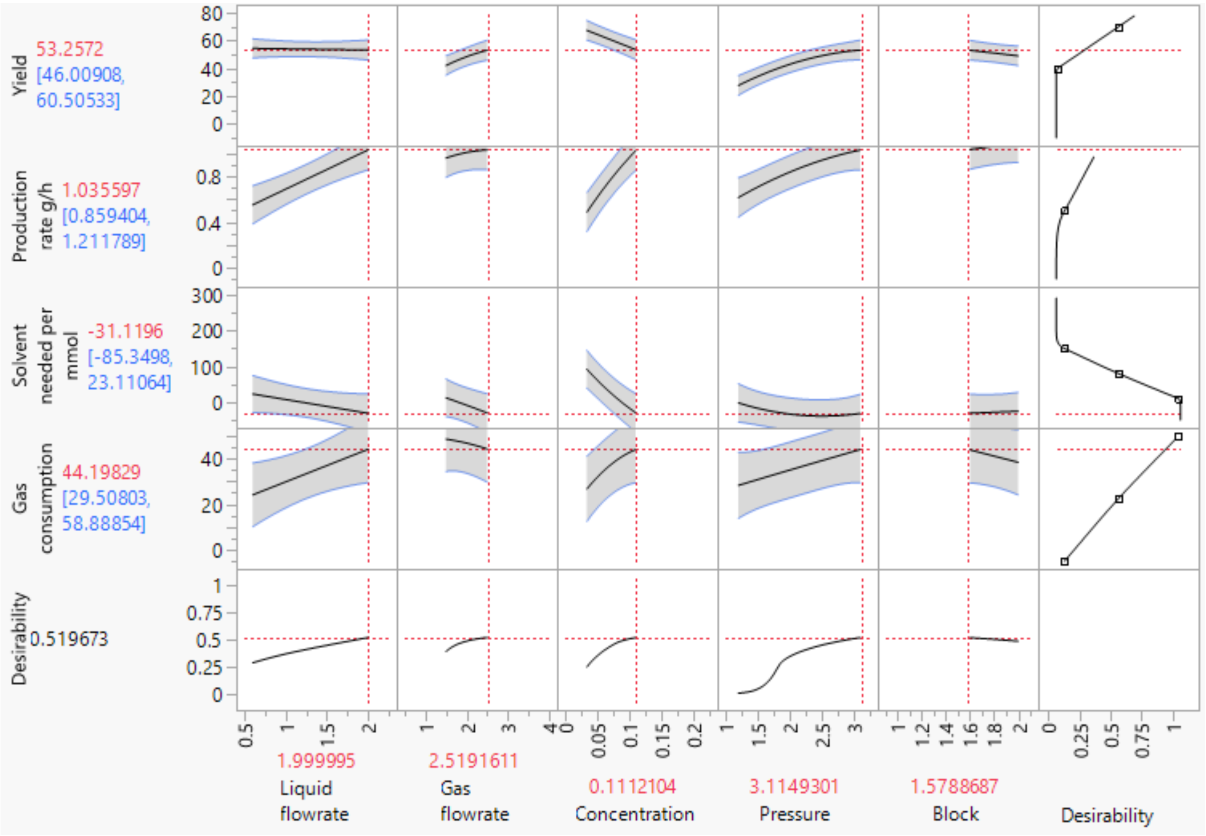

Figure S12: prediction profiler for multi-response model

These conditions were performed experimentally to give a yield of 51%, therefore the responses can be calculated as 6.7 mmol/h or 1.25 g/h production rate, 36% gas consumed and a solvent requirement of 17.8 mL per mmol of product (95.6 mL per g).

These conditions were trialled using 87 mL of 0.11 M **1•PMDETA** (2.972 g, 9.42 mmol) solution with a residence time of 1 min 3s and a process time of approximately 40 minutes. *In situ* yields at several time points including after 30 mins confirmed the 51%. After removing samples for analysis, the remaining crude mixture was purified and desired product was isolated as a colourless oil (660 mg, 3.54 mmol, 39%). These conditions give a space-time yield of 1.48 mol/L/h, a 56-fold increase on batch optimum conditions.

4.1 Reaction of HFO-1234yf with 1•THF in continuous flow

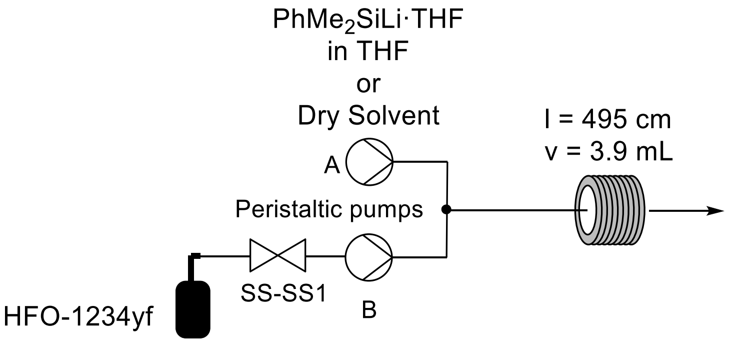

Figure S13: General Schematic for flow reaction

Procedure with optimum process conditions using isolated X·THF:

After being primed with dry THF, the lithium silanide THF adduct (0.11 M) was delivered through peristaltic pump A, set 1.3 mL min^{-1}. HFO-1234yf (1.2 bar) was delivered through peristaltic pump B, set 3.0 mL min^{-1} and mixed at a T junction to give segmented flow through the coil reactor (25 °C, ID = 1 mm, length 495 cm, 3.9 mL reactor volume, T_R = 2 min 26 s).

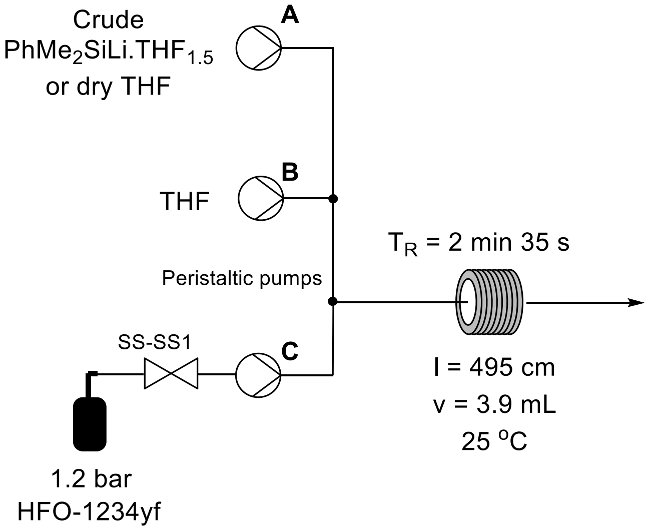

Figure 14: General schematic for dilution of crude 1·THF in flow

Optimum conditions using crude 1·THF:

Prior to the flow procedure filter the crude lithium silanide *via* cannula filtration under a positive flow of nitrogen. After being primed with dry THF, the lithium silanide crude solution (0.45 M) was delivered through pump A at 0.33 mL min^{-1} and mixed at a Y mixer with dry THF set to a flowrate of 0.96 mL min^{-1} delivered through pump B. HFO-1234yf (1.2 bar) was delivered through peristaltic pump C, set to 3.0 mL min^{-1} and mixed at a T junction to give segmented flow through the coil reactor (25 °C, ID = 1 mm, length 495 cm, 3.9 mL reactor volume, T_R = 2 min 35 s). The system was ran for a

process time of 1 h 25 minutes. The crude mixture was collected and purified by silica column chromatography eluted with *n*-pentane to obtain a colourless oil (1.29 g, 5.60 mmol, 32%). These conditions give a space-time yield of 1.05 mol/L/h, an 86-fold increase on batch optimum conditions.

$$Space\ time\ yield\ = \frac{amount\ of\ product\ isolated (mmol)}{volume\ of\ reactor\ (ml) \times process\ time\ (h)} \quad \text{Eq. 1}$$

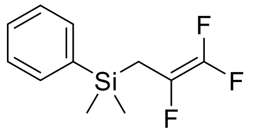

NMR data is consistent with literature.[4]

^{1}H NMR (400 MHz, C_6D_6, 298 K) δ/ppm: 7.33 (m, 2H, *o*-C**H**), 7.16 (m, 3H, *m/p*-C**H**), 1.50 (dt, 2H, $^3J_{HF}$ = 25.1 Hz, $^4J_{HF}$ = 3.7 Hz, C**H**$_2$Si), 0.16 (s, 6H, Si(C**H**$_3$)$_2$).

^{13}C NMR (100 MHz, C_6D_6, 298K) δ/ppm: 153.2 (qd, $^1J_{CF}$ = 269 Hz, $^2J_{CF}$ = 50 Hz, **C**F$_2$), 136.6 (s, Si**C**^{IV}), 133.3 (s, *o*-**C**H), 129.5 (s, *p*-**C**H), 14.3 (d, $^2J_{CF}$ = 26 Hz, **C**H$_2$Si), - 3.5 (s, Si(**C**H$_3$)$_2$).

Missing one aromatic signal due to overlap with solvent.

^{19}F NMR (376 MHz, C_6D_6, 298 K) δ/ppm: -107.8 (dd, 1F, $^2J_{FF}$ = 94.6 Hz, $^3J_{FF}$ = 30.6 Hz, (Z)-C**F**), -126.9 (dd, 1F, $^3J_{FF}$ = 113.7 Hz, $^3J_{FF}$ = 94.6 Hz, (E)-C**F**), -163.6 (dm, 1F, $^3J_{FF}$ = 113.7 Hz, C**F**CH$_2$).

IR (neat)/cm^{-1}: 1789, 1431, 1252, 1200, 1043, 834.

5.1 Difluoromethylation Reactions

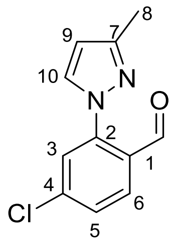

Preparation of 4-chloro-2-(3-methyl-1H-pyrazol-1-yl)benzaldehyde: 2-bromo-4-chlorobenzaldehyde (200 mg, 0.911 mmol), 3-methylpyrazole (144 mg, 1.75 mmol), copper iodide (35.0 mg, 0.184 mmol), potassium carbonate (253 mg, 1.83 mmol) and trans N,N'-dimethylcyclohexane-1,2-diamine (25.9 mg, 0.182 mmol) in 3 mL toluene was stirred at 130 °C (oil bath) for 48 hr. The crude mixture was diluted with ethyl acetate and washed with brine and water then dried over magnesium sulfate and filtered. The solution was concentrated *in vacuo* and purified by silica column chromatography, eluted with 10% ethyl acetate in *n*-pentane. White solid isolated (96.0 mg, 0.435 mmol, 48%).

^{1}H NMR (400 MHz, $CDCl_3$, 298 K) δ/ppm: 10.11 (s, 1H, C**H**O), 7.96 (d, 1H, $^3J_{HH}$ = 8.4 Hz, 6-C**H**$_{Ar}$), 7.73 (d, 1H, $^3J_{HH}$ = 2.4 Hz, 10-C**H**$_{Ar}$), 7.52 (d, 1H, $^4J_{HH}$ = 2.0 Hz, 3-C**H**$_{Ar}$), 7.45 (dd, 1H, $^3J_{HH}$ = 8.4 Hz, $^4J_{HH}$ = 2.0 Hz, 5-C**H**$_{Ar}$), 6.36 (d, 1H, $^3J_{HH}$ = 2.4 Hz, 9-C**H**$_{Ar}$), 2.39 (s, 3H, C**H**$_3$).

^{13}C NMR (100 MHz, $CDCl_3$, 298 K) δ/ppm: 189.3 (s, 1C, **C**HO), 152.3 (s, 1C, 7-**C**$^{IV}_{Ar}$), 143.0 (s, 1C, 2-**C**$^{IV}_{Ar}$), 140.3 (s, 1C, 4-**C**$^{IV}_{Ar}$), 131.3 (s, 1C, 3-**C**H$_{Ar}$), 130.2 (s, 1C, 5-**C**H$_{Ar}$), 128.1 (s, 1C, 1-**C**$^{IV}_{Ar}$), 127.8 (s, 1C, 6-**C**H$_{Ar}$), 124.1 (s, 1C, 10-**C**H$_{Ar}$), 108.7 (s, 1C, 9-**C**H$_{Ar}$), 13.6 (s, 3C, 8-**C**H$_3$).

IR (ATR)/cm^{-1}: 3114, 2899, 1686, (s, C=O stretch), 1591, 1489, 1245, 1096, 1044, 951, 840, 816, 759.

HRMS (ESI-TOF) m/z: $[M + H]^+$ Calcd for $C_{11}H_{10}N_2OCl$ 221.0482; Found 221.0483.

General procedure for difluoromethylation: Aldehyde (0.287 mmol), (difluoromethyl)dimethylphenylsilane, **2** (64.1 mg, 0.344 mmol, 1.2 equiv.) and caesium fluoride (5.8 mg, 13 mol%) were stirred in DMF (3 mL) for 7-16 h in a sealed 20 mL vial in a dinitrogen filled glovebox. TBAF (1 M in THF, 0.6 mL) was added, and the reaction mixture was stirred for 3 hours before being removed from the glovebox. Saturated ammonium chloride solution (15 mL) was added and extracted with diethyl ether (4 x 15 mL). The contained organic phases were washed with water and dried over magnesium sulfate and filtered. The crude mixture was concentrated *in vacuo* by rotary evaporation before being purified by silica column chromatography, eluted with *n*-pentane/ethyl acetate 0-40% unless otherwise stated, using a Biotage automated purification system to give the desired difluoromethylated alcohol products.

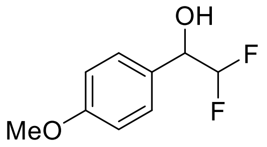

Synthesis of 2,2-difluoro-1-(4-methoxyphenyl)ethan-1-ol, 4a: Yellow oil isolated using general procedure (33.7 mg, 0.179 mmol, 62%). Purified by silica column chromatography, eluted with *n*-pentane/ethyl acetate 0-40% gradient.

NMR data are consistent with literature.[5]

^{1}H NMR (400 MHz, CDCl$_3$, 298 K) δ/ppm: 7.35 (d, 2H, $^3J_{HH}$ = 8.47 Hz, *o*-C**H$_{Ph}$**), 6.93 (d, 2H, $^3J_{HH}$ = 8.47 Hz, *m*-C**H$_{Ph}$**), 5.75 (td, 1H, $^2J_{HF}$ = 55.9 Hz, $^3J_{HH}$ = 4.9 Hz, CF$_2$**H**), 4.77 (td, 1H, $^3J_{HF}$ = 10.1 Hz, $^3J_{HH}$ = 4.9 Hz, C**H(**OH)), 3.82 (s, 3H, OC**H$_3$**), 2.38 (s (br), 1H, O**H**).

^{13}C NMR (100 MHz, CDCl$_3$, 298 K) δ/ppm: 159.9 (s, 1C, **C**$^{IV}_{Ph}$(OCH$_3$)), 128.2 (s, 2C, *o*-**C**H$_{Ph}$), 127.8 (s, 1C, **C**$^{IV}_{Ph}$), 115.7 (t, 1C, $^1J_{CF}$ = 245.3 Hz, **C**F$_2$H), 113.9 (s, 2C, *m*-**C**H$_{Ph}$), 73.1 (t, 1C, $^2J_{CF}$ = 24.7 Hz, **C**H(OH)), 55.1 (s, 3C, O**C**H$_3$).

^{19}F NMR (376 MHz, CDCl$_3$, 298 K) δ/ppm: -127.5 (dd, 2F, $^2J_{HF}$ = 55.9 Hz, $^3J_{HF}$ = 10.1 Hz, C**F$_2$**H).

IR data consistent with literature.[6]

IR (thin film)/cm^{-1}: 3430 (br, OH stretch), 2966 (m, CH stretch), 2841, 1612 (m, C=C, aromatic), 1514, 1463, 1250, 1178, 1114, 1067, 1049, 1032.

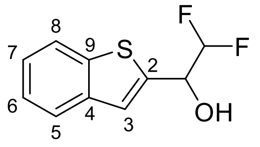

Synthesis of 1-(benzo[b]thiophen-2-yl)-2,2-difluoroethan-1-ol, 4b: Orange solid isolated using general procedure (27.2 mg, 0.127 mmol, 44%). Purified by silica column chromatography, eluted with *n*-pentane/ethyl acetate 0-40% gradient.

NMR and IR data consistent with literature.[7]

^{1}H NMR (400 MHz, CDCl$_3$, 298 K) δ/ppm: 7.85 (d, 1H, $^{3}J_{HH}$ = 7.8 Hz, 6-C**H**$_{Ar}$) , 7.78 (d, 1H, $^{3}J_{HH}$ = 7.8 Hz, 7-C**H**$_{Ar}$), 7.41-7.33 (m, 3H, 3,5,8-C**H**$_{Ar}$), 5.91 (td, 1H, $^{2}J_{HF}$ = 55.9 Hz, $^{3}J_{HH}$ = 4.8 Hz, CF$_2$**H**), 5.17 (td, 1H, $^{3}J_{HF}$ = 10.3 Hz, $^{3}J_{HH}$ = 4.8 Hz, C**H(**OH)), 2.64 (s (br), 1H, O**H**).

^{13}C NMR (100 MHz, CDCl$_3$, 298 K) δ/ppm: δ 139.9 (s, 1C, 4-**C**^{IV}), 139.3 (s, 1C, 9-**C**^{IV}), 139.0 (s, 1C, 2-**C**^{IV}), 125.0 (s, 1C, 3-CH$_{Ar}$), 124.7 (s, 1C, 8-**C**H$_{Ar}$), 124.0 (s, 1C, 5-**C**H$_{Ar}$), 123.4 (s, 1C, 6-**C**H$_{Ar}$), 122.6 (s, 1C, 7-**C**H$_{Ar}$), 115.0 (t, 1C, $^{1}J_{CF}$ = 246.9 Hz, **C**F$_2$H), 70.7 (t, 1C, $^{2}J_{CF}$ = 26.0 Hz, **C**H(OH)).

^{19}F NMR (376 MHz, CDCl$_3$, 298 K) δ/ppm: δ -127.3 (dd, 2F, $^{2}J_{HF}$ = 55.9 Hz, $^{3}J_{HF}$ = 10.3 Hz, C**F$_2$**H).

IR (thin film)/cm^{-1}: 3399 (br, OH stretch), 3056, 2972 (m, CH stretch), 2924, 1434, 1370, 1253, 1121, 1073, 749.

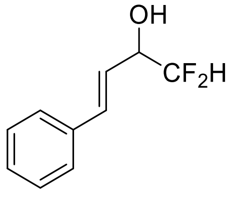

Synthesis of (E)-1,1-difluoro-4-phenylbut-3-en-2-ol, 4c: Orange oil isolated using general procedure (25.3 mg, 0.137 mmol, 46%). Purified using silica column chromatography eluted with 20% ethyl acetate in *n*-hexane.

NMR and IR data consistent with literature.[5]

^{1}H NMR (400 MHz, CDCl$_3$, 298 K) δ/ppm: 7.45 (d, 2H, $^{3}J_{HH}$ = 7.7 Hz, *o*-C**H**$_{Ph}$) , 7.39-7.31 (m, 3H, *m,p*-C**H**$_{Ph}$)), 6.84 (d, 1H, $^{3}J_{HH}$ = 16.1 Hz, C**H**=CH), 6.24 (dd, 1H, $^{3}J_{HH}$ = 16.1 Hz, $^{3}J_{HH}$ = 6.2 Hz, CH=C**H**), 5.75 (td, 1H, $^{2}J_{HF}$ = 56.0 Hz, $^{3}J_{HH}$ = 4.2 Hz, CF$_2$**H**), 4.50 (m, 1H, C**H(**OH)), 2.20 (s (br), 1H, O**H**).

^{13}C NMR (100 MHz, CDCl$_3$, 298 K) δ/ppm: 135.9 (s, 1C, **C**$^{IV}{}_{Ph}$)), 134.9 (s, 1C, *p*-**C**H$_{Ph}$), 128.8 (s, 2C, *o*-**C**H$_{Ph}$), 128.6 (s, 1C, CH=**C**H), 126.9 (s, 2C, m-**C**H$_{Ph}$), 122.6 (t, 1C, $^{3}J_{CF}$ = 3.2 Hz, **C**H=CH), 115.6 (t, 1C, $^{1}J_{CF}$ = 245.3 Hz, **C**F$_2$H), 72.4 (t, 1C, $^{2}J_{CF}$ = 24.6 Hz, **C**H(OH)).

^{19}F NMR (376 MHz, CDCl$_3$, 298 K) δ/ppm: -127.9 (ddd, 1F, $^{2}J_{FF}$ = 286.7 Hz, $^{2}J_{HF}$ = 56.0 Hz, $^{3}J_{HF}$ = 10.6 Hz, C**F$_2$**H), -129.3 (ddd, 1F, $^{2}J_{FF}$ = 286.7 Hz, $^{2}J_{HF}$ = 56.0 Hz, $^{3}J_{HF}$ = 10.6 Hz, C**F$_2$**H).

IR (thin film)/cm^{-1}: 3382, (br, OH stretch), 3027, 1495, 1448, 1124, 1114, 1057, 967, 832, 749, 691.

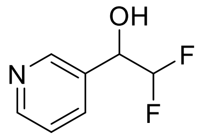

Synthesis of 2,2-difluoro-1-(pyridine-3-yl)ethan-1-ol, 4d: Pale yellow oil isolated using general procedure (33.3 mg, 0.209 mmol, 73%). Purified by silica column chromatography, eluted with *n*-pentane/ethyl acetate 0-40% gradient.

^{1}H NMR (400 MHz, CDCl$_3$, 298 K) δ/ppm: 8.66 (d, 1H, $^{4}J_{HH}$ = 1.4 Hz, *o*-C**H$_{Ar}$**), 8.62 (dd, 1H, $^{3}J_{HH}$ = 4.9 Hz, $^{4}J_{HH}$ = 1.4 Hz, *o*-C**H$_{Ar}$**), 7.84 (d, 1H, $^{3}J_{HH}$ = 7.9 Hz, *p*-C**H$_{Ar}$**), 7.36 (dd, 1H, $^{3}J_{HH}$ = 7.9 Hz, $^{3}J_{HH}$ = 4.9 Hz, *m*-C**H$_{Ar}$**), 5.80 (td, 1H, $^{2}J_{HF}$ = 56.9 Hz, $^{3}J_{HH}$ = 4.5 Hz, CF$_2$**H**), 4.77 (td, 1H, $^{3}J_{HF}$ = 10.3, $^{3}J_{HH}$ = 4.5, C**H**(OH)), 1.28 (s, 1H, O**H**).

^{13}C NMR (100 MHz, CDCl$_3$, 298 K) δ/ppm: 149.8 (s, 1C, *o*-**C**H$_{Ar}$), 148.5 (s, 1C, *p*-**C**H$_{Ar}$), 135.3, (s, 1C, *o*-**C**H$_{Ar}$), 132.1 (s, 1C, **C**$^{IV}_{Ar}$), 123.7 (s, 1C, *m*-**C**H$_{Ar}$), 115.5 (t, 1C, $^{1}J_{CF}$ = 246.0 Hz, **C**F$_2$H), 71.5 (t, 1C, $^{2}J_{CF}$ = 25.1 Hz, **C**H(OH)).

^{19}F NMR (376 MHz, CDCl$_3$, 298 K) δ/ppm: -126.9 (ddd, 1F, $^{2}J_{FF}$ = 283.6 Hz, $^{2}J_{HF}$ = 56.9 Hz, $^{3}J_{HF}$ = 10.3 Hz, C**F$_2$**H), -128.4 (ddd, 1F, $^{2}J_{FF}$ = 283.6 Hz, $^{2}J_{HF}$ = 56.9 Hz, $^{3}J_{HF}$ = 10.3 Hz, C**F$_2$**H).

IR (thin film)/cm^{-1}: 3092 (br, OH stretch), 2924 (m, CH stretch), 2852, 1584, 1481, 1428, 1121, 1055, 856, 803, 712, 550.

HRMS (ESI-TOF) m/z: [M + H]$^+$ Calcd for $C_7H_8NOF_2$ 160.0574; Found 160.0570.

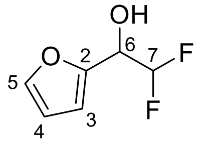

Synthesis of 2,2-difluoro-1-(furan-2-yl)ethan-1-ol, 4e: Brown oil isolated using general procedure (23.7 mg, 0.160 mmol, 56%). Purified by silica column chromatography, eluted with *n*-pentane/ethyl acetate 0-40% gradient.

NMR data consistent with literature.[8]

^{1}H NMR (400 MHz, CDCl$_3$, 298 K) δ/ppm: 7.46 (s, 1H, 5-C**H$_{Ar}$**), 6.47 (d, 1H, $^{3}J_{HH}$ = 3.4 Hz, 3-C**H$_{Ar}$**), 6.41 (t, 1H, $^{3}J_{HH}$ = 3.4 Hz, 4-C**H$_{Ar}$**), 5.97 (td, 1H, $^{2}J_{HF}$ = 55.6 Hz, $^{3}J_{HH}$ = 4.3 Hz, CF$_2$**H**), 4.86 (td, 1H, $^{3}J_{HF}$ = 10.0 Hz, $^{3}J_{HH}$ = 4.3 Hz, C**H**(OH)), 2.27 (s, 1H, O**H**).

^{13}C NMR (100 MHz, CDCl$_3$, 298 K) δ/ppm: 149.2 (s, 1C, 2-**C**$^{IV}_{Ar}$), 143.5 (s, 1C, 5-**C**H$_{Ar}$), 114.4 (t, 1C, $^{1}J_{CF}$ = 245.3 Hz, 7-**C**F$_2$H), 110.7 (s, 1C, 3-**C**H$_{Ar}$), 109.6 (s, 1C, 4-**C**H$_{Ar}$), 67.9 (t, 1C, $^{2}J_{CF}$ = 26.0 Hz, 6-**C**H(OH)).

^{19}F NMR (376 MHz, CDCl$_3$, 298 K) δ/ppm: -127.7 (ddd, 1F, $^{2}J_{FF}$ = 286.6 Hz, $^{2}J_{HF}$ = 55.6 Hz, $^{3}J_{HF}$ = 10.0 Hz, C**F$_2$**H), -129.0 (ddd, 1F, $^{2}J_{FF}$ = 286.6 Hz, $^{2}J_{HF}$ = 55.6 Hz, $^{3}J_{HF}$ = 10.0 Hz, C**F$_2$**H).

IR (thin film)/cm^{-1}: 3400 (br, OH stretch), 1502 (m, C=C stretch), 1374, 1258, 1147, 1120, 1069, 1041 1011.

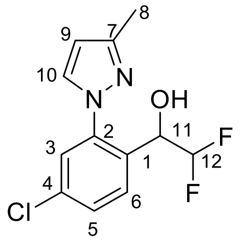

Synthesis of 1-(4-chloro-2-(3-methyl-1H-pyrazol-1-yl)phenyl)-2,2-difluoroethan-1-ol, 4f: White solid isolated (35 mg, 0.128 mmol, 56%) using general procedure with 0.228 mmol aldehyde and heated to 80 ºC for 5 days then purified by automated silica column, eluted with *n*-pentane/ethyl acetate 0-40% followed by precipitation using *n*-pentane.

^{1}H NMR (400 MHz, $CDCl_3$, 298 K) δ/ppm: 7.67 (d, 1H, $^3J_{HH}$ = 2.3 Hz, 10-C**H**$_{Ar}$), 7.54 (d, 1H, $^3J_{HH}$ = 8.3 Hz, 6-C**H**$_{Ar}$), 7.40 (d, 1H, $^3J_{HH}$ = 8.3 Hz, 5-C**H**$_{Ar}$), 7.33 (s, 1H, 3-C**H**$_{Ar}$), 6.33 (d, 1H, $^3J_{HH}$ = 2.3 Hz, 9-C**H**$_{Ar}$), 5.95 (d, 1H, $^3J_{HH}$ = 7.4 Hz, O**H**), 5.59 (td, 1H, $^2J_{HF}$ = 55.8, 5.1 Hz, CF_2**H**), 4.77 (m, 1H, $^3J_{HF}$ = 13.6, $^3J_{HH}$ = 7.4 Hz, C**H**(OH)), 2.38 (s, 3H, C**H**$_3$).

^{13}C NMR (100 MHz, $CDCl_3$, 298 K) δ/ppm: 151.4 (s, 1C, 7-**C**$^{IV}_{Ar}$), 140.1 (s, 1C, 4-**C**$^{IV}_{Ar}$), 135.3 (s, 1C, 2-**C**$^{IV}_{Ar}$), 132.4 (s, 1C, 3-**C**H$_{Ar}$), 131.8 (s, 1C, 5-**C**H$_{Ar}$), 130.8 (s, 1C, 1-**C**$^{IV}_{Ar}$), 128.3 (s, 1C, 6-**C**H$_{Ar}$), 125.1 (s, 1C, 10-**C**H$_{Ar}$), 115.0 (t, 1C, $^1J_{CF}$ = 244.5 Hz, 12-**C**F$_2$H), 108.2 (s, 1C, 9-**C**H$_{Ar}$), 71.7 (t, 1C, $^2J_{CF}$ = 26.2 Hz, 11-**C**H(OH)), 13.6 (s, 3C, 8-**C**H$_3$).

^{19}F NMR (376 MHz, $CDCl_3$ 298 K) δ/ppm: -123.91 (ddd, 1F, $^2J_{FF}$ = 287.1, $^2J_{HF}$ = 55.8, $^3J_{HF}$ = 7.4 Hz, C**F**$_2$H), -127.53 (ddd, 1F, $^2J_{FF}$ = 287.1, $^2J_{HF}$ = 55.8, $^3J_{HF}$ = 13.6 Hz, C**F**$_2$H).

IR (ATR)/cm^{-1}: 3130 (br, OH stretch), 1595, 1362, 1047, 855, 813, 767, 624.

HRMS (ESI-TOF) m/z: $[M + H]^+$ Calcd for $C_{12}H_{12}N_2OClF_2$ 273.0606; Found 273.0617.

6.1 Allylation reactions

General Procedure A:

In a glovebox, under an atmosphere of N_2, the carbonyl substrate (0.226–0.316 mmol, 1 equiv.) and dimethyl(phenyl)(2,3,3-trifluoroallyl)silane, **3** (0.339–0.474 mmol, 1.5 equiv.) were dissolved in 1.5 mL THF and transferred to a 20 mL scintillation vial. α,α,α-trifluorotoluene was added as an ^{19}F NMR internal standard (0.226 mmol–0.316 mmol). TBAF (1 M solution in THF, 5 mol%) was added, the reaction mixture was then stirred for 1 h at room temperature after which an additional equivalent of TBAF was added to ensure complete desilylation of the product. The vial was removed from the glovebox, the solvent evaporated under reduced pressure, and the crude reaction mixture was purified by silica column chromatography eluted with 20% ethyl acetate in *n*-hexane unless otherwise stated. Solid products were precipitated using *n*-pentane.

General procedure B:

In a glovebox, under an atmosphere of N_2, imine substrate (0.170–0.200 mmol, 1 equiv.) and dimethyl(phenyl)(2,3,3-trifluoroallyl)silane, **3** (0.255–0.300 mmol, 1.5 equiv.) were dissolved in 1.5 mL THF and transferred to an ampoule. α,α,α-trifluorotoluene was added as an ^{19}F NMR internal standard (0.170 mmol–0.200 mmol). An initial portion of TBAF (1 M in THF, 15 mol%) was added and the resultant solution was stirred for 2–3 h at 80 °C (oil bath). After cooling, an additional portion of TBAF (1 M in THF, 15 mol%) was added and the reaction was heated again to 80 °C for 2–3 h. This procedure was repeated until a total of 1.05 equivalents TBAF (1 M solution in THF) had been added. The reaction mixture was purified by silica column chromatography eluted with 20% ethyl acetate in *n*-hexane.

General procedure C:

In a glovebox, under an atmosphere of N_2, the carbonyl substrate (0.167 mmol, 1 equiv.) and dimethyl(phenyl)(2,3,3-trifluoroallyl)silane, **3** (0.251 mmol, 1.5 equiv.) were dissolved in 1.5 mL THF and transferred to a 20 mL scintillation vial. The vial was transferred out of the glovebox, and the reaction was stirred at 25 °C under air. TBAF (1 M in THF, 12.5 µl, 8 mol%), used as purchased, was added using a glass micro syringe. The solvent was evaporated under reduced pressure, and the crude mixture was purified according to General Procedure A.

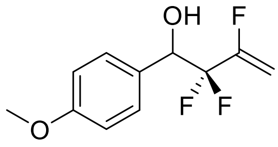

Synthesis of 2,2,3-trifluoro-1-(4-methoxyphenyl)but-3-en-1-ol, 5a: Isolated as a colourless solid (46.3 mg, 0.199 mmol, 78%) using general procedure A. Purified by silica column chromatography eluted with 20% ethyl acetate in *n*-hexane.

The reaction could also be performed on a 0.742 mmol scale. 4-methoxybenzaldehyde (101.0 mg, 0.742 mmol) and dimethyl(phenyl)(2,3,3-trifluoroallyl)silane, **3** (203.0 mg, 0.881 mmol, 1.2 equiv.) were dissolved in 3 mL THF and transferred to a 20 mL scintillation vial. TBAF (1 M solution in THF, 36.5 µL, 5 mol%) was added, the reaction mixture was then stirred for 1 h at room temperature after which an additional equivalent of TBAF was added to ensure complete desilylation of the product. The crude mixture was purified by silica column chromatography eluted with 20% ethyl acetate in *n*-hexane. (144.4 mg, 0.622 mmol, 84%).

NMR data consistent with literature.[9]

^{1}H NMR (400 MHz, $CDCl_3$, 298 K) δ/ppm: 7.40 (d, 2H, $^3J_{HH}$ = 8.5 Hz, *o*-C**H**$_{Ph}$), 6.93 (d, 2H, $^3J_{HH}$ = 8.5 Hz, *m*-C**H**$_{Ph}$), 5.10 – 5.00 (m, 2H, CF=C**H**$_2$), 4.92 (d, 1H, $^3J_{HH}$ = 4.1 Hz, C**H**(OH)), 3.84 (s, 3H, OC**H**$_3$), 2.40 (d, 1H, $^3J_{HH}$ = 4.3 Hz, OH).

^{13}C NMR (125 MHz, $CDCl_3$, 298 K) δ/ppm: 160.2 (s, 1C, **C**^{iV}$_{Ph}$(OCH_3)), 156.5 (ddd, 1C, $^1J_{CF}$ = 261.1 Hz, $^2J_{CF}$ = 31.8 Hz, $^2J_{CF}$ = 31.5 Hz, **C**F=CH_2), 128.9 (s, 2C, *m*-CH$_{Ph}$), 127.0 (s, 1C, **C**^{iV}$_{Ph}$) 115.8 (td, 1C, $^1J_{CF}$ = 247.6 Hz, $^2J_{CF}$ = 37.3 Hz, **C**F$_2$), 113.8 (s, 2C, *o*-**C**H$_{Ph}$), 95.8 (dt, 1C, $^2J_{CF}$ = 13.1 Hz, $^3J_{CF}$ = 4.1 Hz, CF=**C**H$_2$), 73.1 (t, 1C, $^2J_{CF}$ = 27.4 Hz, **C**H(OH)), 55.3 (s, 1C, O**C**H$_3$).

^{19}F NMR (376 MHz, $CDCl_3$, 298 K) δ/ppm: −114.37 (dt, 1F, $^2J_{FF}$ = 263.5, $^3J_{FF}$ = $^4J_{HF}$ = 12.9 Hz, C**F**$_2$), −116.59 (ddd, 1F, $^2J_{FF}$ = 263.5, $^3J_{FF}$ = $^4J_{HF}$ = 11.6 Hz, C**F**$_2$), −117.34 (m, 1F, C**F**=CH_2).

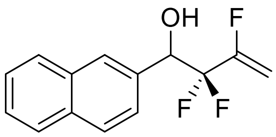

Synthesis of 2,2,3-trifluoro-1-(naphthalen-2-yl)but-3-en-1-ol, 5b: Isolated as a colourless solid (42.0 mg, 0.167 mmol, 65%) using general procedure A. Purified by silica column chromatography eluted with 20% ethyl acetate in *n*-hexane. **5b** was also prepared using general procedure C, 1,2-difluorobenzene was added as a standard and the product was observed *in situ* in 82% yield.

NMR data consistent with literature.[9]

^{1}H NMR (400 MHz, $CDCl_3$, 298 K) δ/ppm: 7.93 (s, 1H, C**H**$_{Ar}$), 7.89 – 7.79 (overlapping, 3H, C**H**$_{Ar}$), 7.57 (d, 1H, $^{3}J_{HH}$ = 8.6 Hz, C**H**$_{Ar}$), 7.51 (m, 2H, C**H**$_{Ar}$), 5.26 (td, 1H, $^{3}J_{HF}$ = 10.3 Hz, $^{3}J_{HH}$ = 4.4 Hz, C**H**(OH)), 5.01 (m, 1H, CF=C**H**(*E*)), 4.95 (dd, 1H, $^{3}J_{HF}$ = 40.6 Hz, $^{2}J_{HH}$ = 4.7 Hz, CF=C**H**(*Z*)), 2.58 (d, 1H, $^{3}J_{HH}$ = 4.4 Hz, CH(O**H**)).

^{13}C NMR (125 MHz, $CDCl_3$, 298 K) δ/ppm: 156.2 (dt, 1C, $^{1}J_{CF}$ = 260.5, $^{2}J_{CF}$ = 32.7 Hz, **C**F=CH_2), 133.6 (s, 1C, **C**$^{iV}_{Ar}$), 132.9 (s, 1C, **C**$^{iV}_{Ar}$), 132.4 (s, 1C, **C**$^{iV}_{Ar}$), 128.2 (s, 1C, **C**H$_{Ar}$), 128.1 (s, 1C, **C**H$_{Ar}$), 127.7 (s, 1C, **C**H$_{Ar}$), 127.4 (s, 1C, **C**H$_{Ar}$), 126.6 (s, 1C, **C**H$_{Ar}$), 126.3 (s, 1C, **C**H$_{Ar}$), 124.8 (s, 1C, **C**H$_{Ar}$), 115.7 (td, 1C, $^{1}J_{CF}$ = 247.8, $^{2}J_{CF}$ = 37.1 Hz, **C**F_2), 96.0 (dt, 1C, $^{2}J_{CF}$ = 13.1, $^{3}J_{CF}$ = 4.0 Hz, CF=**C**H_2)), 73.6 (t, 1C, $^{2}J_{CF}$ = 27.5 Hz, **C**H(OH)).

^{19}F NMR (376 MHz, $CDCl_3$, 298 K) δ/ppm: −113.82 (dt, $^{2}J_{FF}$ = 264.3 Hz, $^{3}J_{FF}$ = $^{3}J_{HF}$ = 11.7 Hz, C**F**$_2$), −116.41 (dt, $^{2}J_{FF}$ = 264.3 Hz, $^{3}J_{FF}$ = $^{3}J_{HF}$ = 11.6 Hz, C**F**$_2$), −117.30 (ddt, 1F, $^{3}J_{HF}$ = 46.5 Hz, $^{3}J_{HF}$ = 14.9 Hz, $^{3}J_{FF}$ = 13.9 Hz, C**F**=CH_2).

HRMS (ESI-TOF) m/z: $[M - H]^-$ Calcd for $C_{14}H_{10}F_3O$ 251.0684; found: 251.0689.

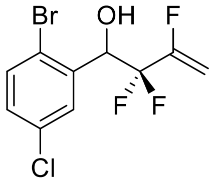

Synthesis of 1-(2-bromo-5-chlorophenyl)-2,2,3-trifluorobut-3-en-1-ol, 5c: Isolated as a colourless solid (41.5 mg, 0.132 mmol, 58%) using general procedure A. Purified by silica column chromatography eluted with 20% ethyl acetate in *n*-hexane.

^{1}H NMR (400 MHz, $CDCl_3$, 298 K) δ/ppm: 7.65 (s, 1H, *o*-C**H**$_{Ph}$), 7.51 (d, 1H, $^{3}J_{HH}$ = 8.5 Hz, *p*-C**H**$_{Ar}$), 7.22 (d, 1H, $^{3}J_{HH}$ = 8.5 Hz, *m*-C**H**$_{Ph}$), 5.62 (m, 1H, C**H**(OH)), 5.12 (m, 1H, CF=C**H**(*Z*)), 5.05 (dd, 1H, $^{3}J_{HF}$ = 46.1 Hz, $^{2}J_{HH}$ = 4.1 Hz, CF=C**H**(*E*)), 2.56 (d, 1H, $^{3}J_{HH}$ = 3.6 Hz, CH(O**H**)).

^{13}C NMR (125 MHz, $CDCl_3$, 298 K) δ/ppm: 155.9 (ddd, 1C, $^{1}J_{CF}$ = 261.1 Hz, $^{2}J_{CF}$ = 29.6 Hz, $^{2}J_{CF}$ = 28.9 Hz, **C**F=CH_2), 136.4 (s, 1C, **C**^{iV}$_{Ph}$), 133.8 (s, 1C, **C**^{iV}$_{Ph}$), 133.7 (s, 1C, *m*-**C**H$_{Ph}$), 130.60 (s, 1C, *o*-**C**H$_{Ph}$), 130.07 (s, 1C, *p*-**C**H$_{Ph}$), 121.9 (s, 1C, **C**^{iV}(Br)), 115.2 (td, 1C, $^{1}J_{CF}$ = 249.7 Hz, $^{2}J_{CF}$ = 34.7 Hz, **C**F_2), 96.3 (dt, 1C, $^{2}J_{CF}$ = 13.1 Hz, $^{3}J_{CF}$ = 4.3 Hz, CF=**C**H_2), 71.3 (dd, 1C, $^{2}J_{CF}$ = 30.8 Hz, $^{2}J_{CF}$ = 25.4 Hz, **C**H(OH)).

^{19}F NMR (376 MHz, $CDCl_3$, 298 K) δ/ppm: −111.48 (ddd, 1F, $^{2}J_{FF}$ = 266.4 Hz, $^{3}J_{FF}$ = 16.3 Hz, $^{3}J_{HF}$ = 7.7 Hz, C**F**$_2$), −117.10 (dddd, 1F, $^{3}J_{HF}$ = 46.1 Hz, $^{3}J_{HF}$ = 16.8 Hz, $^{3}J_{FF}$ = 16.3 Hz, $^{3}J_{FF}$ = 14.0 Hz, C**F**=CH_2), −118.83 (ddd, 1F, $^{2}J_{FF}$ = 266.4 Hz, $^{3}J_{FF}$ = 14.0 Hz, $^{3}J_{HF}$ = 8.3 Hz, C**F**$_2$).

HRMS (ESI-TOF) m/z: $[M - H]^-$ Calcd for $C_{10}H_6BrClF_3O$ 312.9243; found: 312.9251.

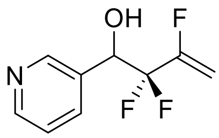

Synthesis of 2,2,3-trifluoro-1-(pyridin-3-yl)but-3-en-1-ol, 5d: Isolated as a colourless solid (48.3 mg, 0.238 mmol, 83%) using general procedure A and purified by silica column chromatography eluted with 50% ethyl acetate in *n*-hexane.

^{1}H NMR (400 MHz, $CDCl_3$, 298 K) δ/ppm: 8.63 (s, 1H, *o*-**C**H$_{Ar}$), 8.59 (d, 1H, $^{3}J_{HH}$ = 4.9 Hz, *p*-C**H**$_{Ar}$), 7.84 (d, 1H, $^{3}J_{HH}$ = 8.0 Hz, *o*-CH$_{Ar}$), 7.34 (dd, $^{3}J_{HH}$ = 8.0 Hz, $^{3}J_{HH}$ = 4.9 Hz, *m*-CH$_{Ar}$), 5.18 – 5.08 (overlapping, 2H, CF=C**H**(*Z*), C**H**(OH)), 5.00 (dd, 1H, $^{3}J_{HF}$ = 46.2 Hz, $^{2}J_{HH}$ = 4.2 Hz, CF=C**H**(*E*)), 3.27 (d, 1H, $^{3}J_{HH}$ = 4.4 Hz, CH(O**H**)).

^{13}C NMR (100 MHz, $CDCl_3$, 298 K) δ/ppm: 155.7 (ddd, 1C, $^{1}J_{CF}$ = 260.8 Hz, $^{2}J_{CF}$ = 31.2 Hz, $^{2}J_{CF}$ = 30.5 Hz, **C**F=CH_2), 149.7 (s, 1C, *o*-CH$_{Ar}$), 148.8 (s, 1C, *p*-**C**H$_{Ar}$), 135.8 (s, 1C, *o*-**C**H$_{Ar}$), 131.7 (s, 1C, **C**$^{iV}_{Ar}$), 123.4 (s, 1C, *m*-**C**H$_{Ar}$), 115.5 (td, 1C, $^{1}J_{CF}$ = 246.8 Hz, $^{2}J_{CF}$ = 37.5 Hz, **C**F_2), 96.3 (dt, 1C, $^{2}J_{CF}$ = 13.1 Hz, $^{3}J_{CF}$ = 4.2 Hz, CF=**C**H_2), 71.1 (dd, 1C, $^{2}J_{CF}$ = 30.0 Hz, $^{2}J_{CF}$ = 26.1 Hz, **C**H(OH)).

^{19}F NMR (376 MHz, $CDCl_3$, 298 K) δ/ppm: −113.47 (ddd, 1F, $^{2}J_{FF}$ = 265.7 Hz, $^{3}J_{FF}$ = $^{3}J_{HF}$ = 10.7 Hz, C**F**$_2$), −117.32 (dt, 1F, $^{2}J_{FF}$ = 265.7 Hz, $^{3}J_{FF}$ = $^{3}J_{HF}$ = 11.1 Hz, C**F**$_2$), −117.45 (ddt, 1F, $^{3}J_{HF}$ = 44.4 Hz, $^{3}J_{HF}$ = 15.3 Hz, $^{3}J_{FF}$ = 14.2 Hz, C**F**=CH_2).

HRMS (ESI-TOF) m/z: $[M-H]^-$ Calcd for $C_9H_7F_3NO$ 202.0480; found: 202.0480.

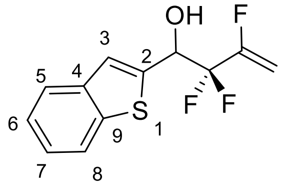

Synthesis of 1-(benzo[b]thiophen-2-yl)-2,2,3-trifluorobut-3-en-1-ol, 5e: Isolated as an off-white solid (51.7 mg, 0.200 mmol, 80%) using general procedure A. Purified by silica column chromatography eluted with 20% ethyl acetate in *n*-hexane

^{1}H NMR (400 MHz, CDCl$_3$, 298 K) δ/ppm: 7.90 – 7.75 (m, 2H, 6,7-C**H**$_{Ar}$), 7.43 (s, 1H, 3-C**H**$_{Ar}$), 7.39 (dd, 2H, $^{3}J_{HH}$ = 6.3, $^{5}J_{HH}$ = 2.7 Hz, 5,8-C**H**$_{Ar}$), 5.45 (td, 1H, $^{3}J_{HF}$ = $^{3}J_{HF}$ = 10.9 Hz, $^{3}J_{HH}$ = 5.3 Hz, C**H**(OH)), 5.19 – 5.04 (m, 2H, CF=C**H**$_2$), 2.67 (d, 1H, $^{3}J_{HH}$ = 5.3 Hz, CH(O**H**)).

^{13}C NMR (125 MHz, CDCl$_3$, 298 K) δ/ppm: 155.7 (dt, 1C, $^{1}J_{CF}$ = 260.4 Hz, $^{2}J_{CF}$ = $^{2}J_{CF}$ = 31.1 Hz, **C**F=CH$_2$), 139.9 (s, 1C, **C**$^{iV}{}_{Ar}$), 138.9 (s, 1C, **C**$^{iV}{}_{Ar}$), 137.9 (s, 1C, **C**$^{iV}{}_{Ar}$), 124.9 (s, 1C, **C**H$_{Ar}$), 124.5 (s, 1C, **C**H$_{Ar}$), 124.2 (s, 1C, **C**H$_{Ar}$), 123.9 (s, 1C, **C**H$_{Ar}$), 122.4 (s, 1C, 3-**C**H$_{Ar}$), 115.1 (td, 1C, $^{1}J_{CF}$ = 248.4 Hz, $^{2}J_{CF}$ = 37.3 Hz, **C**F$_2$), 96.4 (dt, 1C, $^{2}J_{CF}$ = 13.1 Hz, $^{3}J_{CF}$ = 3.9 Hz, CF=**C**H$_2$), 70.5 (t, 1C, $^{3}J_{CF}$ = $^{3}J_{CF}$ = 29.6 Hz, **C**H(OH)).

^{19}F NMR (376 MHz, CDCl$_3$, 298 K) δ/ppm: −113.26 (ddd, 1F, $^{2}J_{FF}$ = 264.6 Hz, $^{3}J_{FF}$ = 14.4 Hz, $^{3}J_{HF}$ = 9.2 Hz, C**F**$_2$), −116.81 (dt, 1F, $^{2}J_{FF}$ = 264.6 Hz, $^{3}J_{FF}$ = $^{3}J_{HF}$ = 11.6 Hz, C**F**$_2$), −118.14 (dm, $^{3}J_{HF}$ = 46.9 Hz, C**F**=CH$_2$).

HRMS (ESI-TOF) m/z: [M − H]$^{-}$ Calcd for $C_{12}H_8F_3OS$ 257.0248; found: 257.0253.

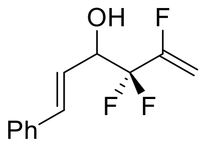

Synthesis of (E)-4,4,5-trifluoro-1-phenylhexa-1,5-dien-3-ol, 5f: Isolated as a pale yellow oil (36.3 mg, 0.159 mmol, 59%) using general procedure A with 1.2 equiv. dimethyl(phenyl)(2,3,3-trifluoroallyl)silane. Purified by silica column chromatography eluted with 20% ethyl acetate in *n*-hexane.

^{1}H NMR (400 MHz, CDCl$_3$, 298 K) δ/ppm: δ 7.45 (d, 2H, $^3J_{HH}$ = 6.6 Hz, *o*-C**H**$_{Ph}$), 7.37 (t, 2H, $^3J_{HH}$ = $^3J_{HH}$ = 7.4 Hz, *m*-C**H**$_{Ph}$), 7.33 (m, 1H, *p*-C**H**$_{Ph}$), 6.84 (d, 1H, $^3J_{HH}$ = 15.9 Hz, CH=C**H**(Ph)), 6.27 (d, 1H, $^3J_{HH}$ = 15.9 Hz, C**H**=CH(Ph)), 5.21 – 5.08 (m, 2H, CF=C**H**$_2$), 4.70 (td, 1H, $^3J_{HF}$ = $^3J_{HF}$ = 10.6 Hz, $^3J_{HH}$ = 5.4 Hz, C**H**(OH)), 2.31 (d, 1H, $^3J_{HH}$ = 5.4 Hz, CH(O**H**)).

^{13}C NMR (125 MHz, CDCl$_3$, 298 K) δ/ppm: 156.3 (dt, 1C, $^1J_{CF}$ = 260.8 Hz, $^2J_{CF}$ = 32.4 Hz, **C**F=CH$_2$), 135.7 (s, 1C, C$^{iV}{}_{Ph}$), 135.5 (s, 1C, CH=**C**H(Ph)), 128.7, (s, 2C, *m*-**C**H$_{Ph}$) 128.5 (s, 1C, *p*-**C**H$_{Ph}$), 126.8 (s, 2C, *o*-**C**H$_{Ph}$), 121.9 (s, 1C, **C**H=CH(Ph)), 115.7 (td, 1C, $^1J_{CF}$ = 247.0 Hz, $^2J_{CF}$ = 36.5 Hz, **C**F$_2$), 95.95 (dt, 1C, $^2J_{CF}$ = 13.3 Hz, $^3J_{CF}$ = 4.1 Hz, CF=**C**H$_2$), 72.46 (t, 1C, $^2J_{CF}$ = 28.2 Hz, **C**H(OH)).

^{19}F NMR (376 MHz, CDCl$_3$, 298 K) δ/ppm: −114.10 (ddd, 1F, $^2J_{FF}$ = 265.8 Hz, $^3J_{FF}$ = 14.6 Hz, $^3J_{HF}$ = 9.9 Hz, C**F**$_2$), −117.38 (dt, 1F, $^2J_{FF}$ = 265.8 Hz, $^3J_{FF}$ = $^3J_{HF}$ = 11.8 Hz, C**F**$_2$), −117.61 (dm, 1F, $^3J_{HF}$ = 45.0 Hz, C**F**=CH$_2$).

HRMS (ESI-TOF) m/z: [M − H]$^-$ Calcd for $C_{12}H_{10}F_3O$ 227.0684; found: 227.0687.

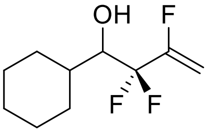

Synthesis of 1-cyclohexyl-2,2,3-trifluorobut-3-en-1-ol, 5g: Pale yellow oil isolated (43.5 mg, 0.209 mmol, 66%) using general procedure A and purified by silica column chromatography eluted with 10% ethyl acetate in *n*-hexane.

^{1}H NMR (400 MHz, CDCl$_3$, 298 K) δ/ppm: 5.08 (m, 2H, CF=C**H**$_2$), 3.75 (m, 1H, C**H**(OH)), 1.93 (d, 1H, $^3J_{HH}$ = 7.4 Hz, CH(O**H**)), 1.88 (d, 2H, $^3J_{HH}$ = 12.7 Hz, C**H**$_2$), 1.82 – 1.59 (overlapping, 5H, C**H**/C**H**$_2$), 1.40 – 1.08 (overlapping, 4H, C**H**$_2$).

^{13}C NMR (100 MHz, CDCl$_3$, 298 K) δ/ppm: 157.3 (dt, 1C, $^1J_{CF}$ = 260.9 Hz, $^2J_{CF}$ = 34.5 Hz, **C**F=CH$_2$), 117.1 (td, 1C, $^1J_{CF}$ = $^1J_{CF}$ = 247.5 Hz, $^2J_{CF}$ = 37.0 Hz, **C**F$_2$), 95.3 (dt, 1C, $^2J_{CF}$ = 13.1 Hz, $^3J_{CF}$ = $^3J_{CF}$ = 4.1 Hz, CF=**C**H$_2$), 74.8 (t, 1C, $^2J_{CF}$ = 26.0 Hz, **C**H(OH)), 38.3 (s, 1C, **C**H), 30.4 (s, 1C, **C**H$_2$), 26.7 (s, 1C, **C**H$_2$), 26.4 (s, 1C, **C**H$_2$), 26.2 (s, 1C, **C**H$_2$), 26.0 (s, 1C, **C**H$_2$).

^{19}F NMR (376 MHz, CDCl$_3$, 298 K) δ/ppm: −111.67 (dt, 1F, $^2J_{FF}$ = 266.7 Hz, $^3J_{FF}$ = $^3J_{HF}$ = 12.3 Hz, C**F**$_2$), −114.51 (ddd, 1F, $^2J_{FF}$ = 266.7 Hz, $^3J_{FF}$ = 15.9 Hz, $^3J_{HF}$ = 10.4 Hz, C**F**$_2$), −117.57 (dm, 1F, $^3J_{HF}$ = 47.1 Hz, C**F**=CH$_2$).

HRMS (ESI-TOF) m/z: [M − H]$^-$ Calcd for $C_{10}H_{14}F_3O$ 207.0997; found: 207.0998.

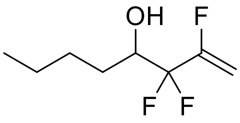

Synthesis of 2,3,3-trifluorooct-1-en-4-ol, 5h: Isolated as a pale yellow oil (51.6 mg, 0.283 mmol, 69%) using procedure A and purified by silica column chromatography eluted with 20% diethyl ether in *n*-pentane.

^{1}H NMR (400 MHz, CDCl$_3$, 298 K) δ/ppm: 5.09 (m, 2H, CF=C**H**$_2$), 3.93 (td, 1H, $^{3}J_{HF}$ = $^{3}J_{HF}$ = 12.1 Hz, $^{3}J_{HH}$ = 6.5 Hz, C**H**(OH)), 1.92 (d, 1H, $^{3}J_{HH}$ = 6.5 Hz, CH(O**H**)), 1.74 – 1.47 (overlapping, 3H, C**H**$_2$), 1.44 – 1.30 (overlapping, 3H, C**H**$_2$), 0.92 (t, 3H $^{3}J_{HH}$ = 7.0 Hz, C**H**$_3$).

^{13}C NMR (125 MHz, CDCl$_3$, 298 K) δ/ppm: 156.8 (dt, 1C, $^{1}J_{CF}$ = 259.9 Hz, $^{2}J_{CF}$ = 32.8 Hz, **C**F=CH$_2$), 116.6 (td, 1C, $^{1}J_{CF}$ = 246.0 Hz, $^{2}J_{CF}$ = $^{2}J_{CF}$ = 37.1 Hz, **C**F$_2$), 95.7 (dt, 1C, $^{2}J_{CF}$ = 12.9 Hz, $^{3}J_{CF}$ = 4.1 Hz, CF=**C**H$_2$), 71.5 (t, 1C, $^{2}J_{CF}$ = 27.2 Hz, **C**H(OH)), 29.4 (s, 1C, CH$_2$), 27.7 (s, 1C, CH$_2$), 22.6 (s, 1C, CH$_2$), 14.0 (s, 1C, **C**H$_3$).

^{19}F NMR (376 MHz, CDCl$_3$, 298 K) δ/ppm: −115.41 (dt, 1F, $^{2}J_{FF}$ = 264.7 Hz, $^{3}J_{FF}$ = $^{3}J_{HF}$ = 11.4 Hz, C**F**$_2$), −117.42 (dm, 1F, $^{3}J_{HF}$ = 47.1 Hz, C**F**=CH$_2$), −118.14 (dt, 1F, $^{2}J_{FF}$ = 264.7 Hz, $^{3}J_{FF}$ = $^{3}J_{HF}$ = 11.8 Hz, C**F**$_2$).

HRMS (ESI-TOF) m/z: [M − H]$^-$ Calcd for $C_8H_{12}F_3O$ 181.0840; found: 181.0838.

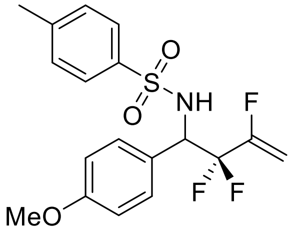

Synthesis of 4-methyl-N-(2,2,3-trifluoro-1-(4-methoxyphenyl)but-3-en-1-yl)benzenesulfonamide, 5i: Isolated as a colourless solid (46.3 mg, 0.120 mmol, 58%) using general procedure B, purified by silica column chromatography eluted with 25% ethyl acetate in *n*-hexane.

^{1}H NMR (400 MHz, CDCl$_3$, 298 K) δ/ppm: 7.54 (d, 2H, $^{3}J_{HH}$ = 8.6 Hz, *o*-C**H**$_{Ph}$), 7.12 (d, 2H, $^{3}J_{HH}$ = 8.0 Hz, *o*-C**H**$_{Ph}$), 7.03 (d, 2H, $^{3}J_{HH}$ = 8.0 Hz, *m*-C**H**$_{Ph}$), 6.71 (d, 2H, $^{3}J_{HH}$ = 8.6 Hz, *m*-C**H**$_{Ph}$), 5.38 (d, 1H, $^{3}J_{HH}$ = 8.1 Hz, N**H**), 4.98 – 4.77 (overlapping, 3H, CF=C**H**$_2$, C**H**(NH)), 3.76 (s, 3H, OC**H**$_3$), 2.35 (s, 3H, C**H**$_3$).

^{13}C NMR (125 MHz, CDCl$_3$, 298 K) δ/ppm: 160.1 (s,1C, **C**$^{iV}_{Ph}$(OCH$_3$), 155.7 (dt, 1C, $^{1}J_{CF}$ = 260.4 Hz, $^{2}J_{CF}$ = 32.6 Hz, **C**F=CH$_2$), 143.6 (s, 1C, **C**$^{iV}_{Ph}$), 137.3 (s, 1C, **C**$^{iV}_{Ph}$), 129.5 (s, 4C, *o*-**C**H$_{Ph}$), 127.2 (s, 2C, *m*-**C**H$_{Ph}$), 124.9 (s, 1C, **C**$^{iV}_{Ph}$), 116.6 (td, 1C, $^{1}J_{CF}$ = 248.8 Hz, $^{2}J_{CF}$ = 38.5 Hz, **C**F$_2$), 114.0 (s, 2C, *m*-**C**H$_{Ph}$), 96.3 (dt, 1C, $^{2}J_{CF}$ = 13.1, $^{3}J_{CF}$ = 3.8 Hz, CF=**C**H$_2$), 59.0 (t, 1C, $^{2}J_{CF}$ = 26.3 Hz, **C**H(NH)), 55.4 (s, 1C, O**C**H$_3$), 21.6 (s, 1C, **C**H$_3$).

^{19}F NMR (376 MHz, CDCl$_3$, 298 K) δ/ppm: −111.71 (dt, 1F, $^{2}J_{FF}$ = 263.0 Hz, $^{3}J_{FF}$ = $^{3}J_{HF}$ = 13.5 Hz, C**F**$_2$), −112.48 (dt, 1F, $^{2}J_{FF}$ = 263.0 Hz, $^{3}J_{FF}$ = $^{3}J_{HF}$ = 13.3 Hz, C**F**$_2$), −117.60 (ddt, 1F, $^{3}J_{HF}$ = 48.0 Hz, $^{3}J_{HF}$ = 13.9 Hz, $^{3}J_{FF}$ = 13.4 Hz, C**F**=CH$_2$).

HRMS (ESI-TOF) m/z: [M − H]$^-$ Calcd for $C_{18}H_{17}F_3NO_3S$ 384.0881; found: 384.0885.

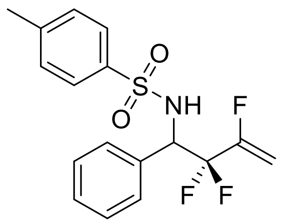

Synthesis of 4-methyl-N-(2,2,3-trifluoro-1-phenylbut-3-en-1-yl)benzenesulfonamide, 5j: Isolated as a colourless solid (43.9 mg, 0.124 mmol, 65%) using general procedure B. Purified by silica column chromatography eluted with 20% ethyl acetate in *n*-hexane.

^{1}H NMR (400 MHz, $CDCl_3$, 298 K) δ/ppm: 7.54 (d, 2H, $^3J_{HH}$ = 8.1 Hz, *o*-C**H**$_{Ph}$), 7.26 – 7.17 (overlapping, 3H, *o,p*-C**H**$_{Ph}$), 7.14 – 7.08 (overlapping, 4H, *m*-C**H**$_{Ph}$), 5.34 (d, 1H, $^3J_{HF}$ = 9.4 Hz, N**H**), 4.98 – 4.87 (overlapping, 2H, C**H**(NH),CF=C**H**(*Z*)), 4.87 (dd, 1H, $^3J_{HF}$ = 47.5 Hz, $^3J_{HH}$ = 4.0 Hz, CF=C**H**(*E*)), 2.34 (s, 3H, C**H**$_3$).

^{13}C NMR (125 MHz, $CDCl_3$, 298 K) δ/ppm: 155.4 (dt, 1C, $^1J_{CF}$ = 260.5 Hz, $^2J_{CF}$ = 32.4 Hz**,** **C**F=CH$_2$), 143.5 (s, 1C, **C**^{iV}(SO$_2$)), 137.1 (s, 1C, **C**$^{iV}{}_{Ph}$), 132.8 (s, 1C, **C**$^{iV}{}_{Ph}$(CH$_3$)), 129.3 (s, 2C, **C**H$_{Ph}$), 128.8 (s, 1C, **C**H$_{Ph}$), 128.5 (s, 2C, **C**H$_{Ph}$), 128.1 (s, 2C, **C**H$_{Ph}$), 127.0 (s, 2C, **C**H$_{Ph}$), 115.4 (td, 1C, $^1J_{CF}$ = $^1J_{CF}$ = 249.4 Hz, $^2J_{CF}$ = 39.0 Hz, **C**F$_2$), 96.3 (dt, 1C, $^2J_{CF}$ = 13.0 Hz, $^3J_{CF}$ = $^3J_{CF}$ = 3.8 Hz, CF=**C**H$_2$), 59.4 (t, 1C, $^2J_{CF}$ = 26.6 Hz, **C**H(NH)), 21.5 (s, 1C, CH$_3$).

^{19}F NMR (376 MHz, $CDCl_3$, 298 K) δ/ppm: −111.56 (dt, 1F, $^2J_{FF}$ = 262.9 Hz, $^3J_{FF}$ = 13.0 Hz, C**F**$_2$), −112.43 (dt, 1F, $^2J_{FF}$ = 262.9 Hz, $^3J_{FF}$ = 12.9 Hz, C**F**$_2$), −117.57 (ddt, 1F, $^3J_{HF}$ = 47.5 Hz, $^3J_{HF}$ = 14.1 Hz, $^3J_{FF}$ = 13.0 Hz, C**F**=CH$_2$).

HRMS (ESI-TOF) m/z: $[M + H]^+$ Calcd for $C_{17}H_{17}F_3NO_2S$ 356.0932; found: 356.0929.

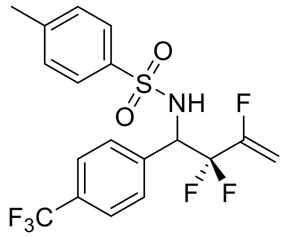

Synthesis of 4-methyl-N-(2,2,3-trifluoro-1-(4-(trifluoromethyl)phenyl)but-3-en-1-yl)benzenesulfonamide, 5k: Isolated as a colourless solid (42.0 mg, 0.0992 mmol, 58%) using general procedure B with 1.2 equiv. dimethyl(phenyl)(2,3,3-trifluoroallyl)silane. Purified by silica column chromatography eluted with 20% ethyl acetate in *n*-hexane.

^{1}H NMR (400 MHz, $CDCl_3$, 298 K) δ/ppm: 7.52 (d, 2H, $^{3}J_{HH}$ = 8.3 Hz, *o*-C**H**$_{Ph}$), 7.44 (d, 2H $^{3}J_{HH}$ = 8.1 Hz, *m*-C**H**$_{Ph}$), 7.26 (m, 2H, *o*-C**H**$_{Ph}$), 7.09 (d, 2H, $^{3}J_{HH}$ = 8.3 Hz, *m*-C**H**$_{Ph}$), 5.82 (d, 1H, $^{3}J_{HH}$ = 9.2 Hz, N**H**), 5.06 – 4.88 (overlapping, 3H, C**H**(NH), CF=C**H**$_2$), 2.35 (s, 3H, C**H**$_3$).

^{13}C NMR (125 MHz, $CDCl_3$, 298 K) δ/ppm: 155.1 (dt, 1C, $^{1}J_{CF}$ = 260.8 Hz, $^{2}J_{CF}$ = 32.7 Hz, **C**F=CH$_2$), 144.1 (s, 1C, **C**$^{iV}_{Ph}$(SO$_2$)), 136.8 (s, 1C, **C**$^{iV}_{Ph}$), 136.6 (s, 1C, **C**$^{iV}_{Ph}$), 131.1 (q, 1C, $^{2}J_{CF}$ = 32.7 Hz, **C**$^{iV}_{Ph}$(CF$_3$)), 129.5 (s, 2C, C**H**$_{Ph}$), 128.9 (s, 2C, C**H**$_{Ph}$), 127.2 (s, 2C, C**H**$_{Ph}$), 125.5 (q, 2C, $^{3}J_{CF}$ = 3.5 Hz, *m*-**C**H$_{Ph}$), 123.9 (q, 1C, $^{1}J_{CF}$ = 270.7 Hz, **C**F$_3$), 115.2 (td, 1C, $^{1}J_{CF}$ = 249.2 Hz, $^{2}J_{CF}$ = 39.1 Hz, **C**F$_2$), 96.9 (dt, $^{2}J_{CF}$ =12.9 Hz, $^{3}J_{CF}$ = 3.2 Hz, CF=**C**H$_2$) 59.1 (dd, 1C, $^{2}J_{CF}$ = 29.2, $^{2}J_{CF}$ = 24.9 Hz, **C**H(NH)), 21.4 (s, 1C, **C**H$_3$).

^{19}F NMR (376 MHz, $CDCl_3$, 298 K) δ/ppm: −62.90 (s, 3F, C**F**$_3$), −110.50 (dt, 1F, $^{2}J_{FF}$ = 264.8 Hz, $^{3}J_{FF}$ = $^{3}J_{HF}$ = 12.6 Hz), −113.32 (dt, 1F, $^{2}J_{FF}$ = 264.8 Hz, $^{3}J_{FF}$ = $^{3}J_{HF}$ = 13.0 Hz), −117.91 (ddt, 1F, $^{3}J_{HF}$ = 47.9 Hz, $^{3}J_{HF}$ = 13.6 Hz, $^{3}J_{FF}$ = 12.9 Hz, C**F**=CH$_2$).

HRMS (ESI-TOF) m/z: $[M - H]^-$ Calcd for $C_{18}H_{14}F_6NO_2S$ 422.0649; found: 422.0655.

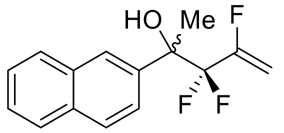

Synthesis of 3,3,4-trifluoro-2-(naphthalen-2-yl)pent-4-en-2-ol, 5l: Isolated as an orange oil isolated containing 15% starting ketone (38.0 mg, 0.121 mmol, 47%) using general procedure A with 4x5 mol% TBAF added in portions over 4 h and purified by silica column chromatography eluted with 13% ethyl acetate in *n*-hexane.

^{1}H NMR (400 MHz, $CDCl_3$, 298 K) δ/ppm: 8.05 (s, 1H, 1-C**H**$_{Ar}$), 7.89 – 7.81 (overlapping, 3H, C**H**$_{Ar}$), 7.65 (m, 2H, C**H**$_{Ar}$), 7.50 (m, 2H, C**H**$_{Ar}$), 4.93 (dd, 1H, $^3J_{HF}$ = 16.6 Hz, $^3J_{HH}$ = 4.0 Hz, CF=C**H**(*Z*)), 4.77 (dd, 1H, $^3J_{HF}$ = 46.2 Hz, $^2J_{HH}$ = 4.0 Hz, CF=C**H**(*E*)), 2.52 (s, 1H, C(O**H**)), 1.87 (s, 3H, C**H**$_3$).

^{13}C NMR (100 MHz, $CDCl_3$, 298 K) δ/ppm: 156.5 (dt, 1C, $^1J_{CF}$ = 263.6 Hz, $^2J_{CF}$ = 32.7 Hz, **C**F=CH_2), 128.6 (s, 1C, **C**H$_{Ar}$), 128.5 (s, 1C, **C**^{iv}$_{Ar}$), 127.7 (s, 1C, **C**H$_{Ar}$), 127.6(s, 1C, **C**H$_{Ar}$), 126.9 (s, 1C, **C**^{iv}$_{Ar}$), 126.5 (s, 1C, **C**H$_{Ar}$), 126.3 (s, 1C, **C**H$_{Ar}$), 125.7 (s, 1C, **C**H$_{Ar}$), 124.2 (s, 1C, **C**H$_{Ar}$), 124.0 (s, 1C, **C**^{iv}$_{Ar}$), 117.1 (td, 1C, $^1J_{CF}$ = 252.3 Hz, $^2J_{CF}$ = 31.7 Hz, **C**F$_2$), 96.8 (dt, 1C, $^2J_{CF}$ = 13.7, $^3J_{CF}$ = 4.3 Hz, CF=**C**H$_2$), 76.3 (d, 1C, $^2J_{CF}$ = 26.9 Hz, **C**(OH)), 24.2 (s, 1C, **C**H$_3$).

^{19}F NMR (376 MHz, $CDCl_3$, 298 K) δ/ppm: −111.73 (ddt, 1F, $^3J_{HF}$ = 46.2 Hz, $^3J_{HF}$ = 16.6 Hz, $^3J_{FF}$ = $^3J_{FF}$ = 13.0 Hz, C**F**=CH_2), −112.64 (dd, 1F, $^2J_{FF}$ = 263.8 Hz, $^3J_{FF}$ = 12.8 Hz, C**F**$_2$), −114.57 (dd, 1F, $^2J_{FF}$ = 263.8 Hz, $^3J_{FF}$ = 13.4 Hz, C**F**$_2$).

HRMS (ESI-TOF) m/z: $[M-H]^-$ Calcd for $C_{15}H_{12}F_3O$ 265.0840; found: 265.0846.

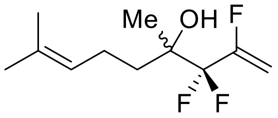

Synthesis of 2,3,3-trifluoro-4,8-dimethylnona-1,7-dien-4-ol, 5m: Isolated as a yellow oil, 36.6 mg, 0.165 mmol, 59%) using general procedure A with 3x5 mol% TBAF added in portions over 3 h and purified by silica column chromatography eluted with 10% ethyl acetate in *n*-hexane.

NMR data consistent with literature.[9]

^{1}H NMR (400 MHz, CDCl$_3$, 298 K) δ/ppm: 5.15 – 5.05 (overlapping, 2H, C=C**H**, CF=C**H**$_2$(*Z*)), 5.02 (dd, 1H, $^{3}J_{HF}$ = 46.3 Hz, $^{2}J_{HH}$ = 3.8 Hz, CF=C**H**(*E*)), 2.14 (m, 2H, C**H**$_2$), 1.91 (s, 1H, C(O**H**)), 1.69 (s, 3H, C**H**$_3$C=CH), 1.66 (m, 2H, C**H**$_2$) 1.63 (s, 3H, C**H**$_3$C=CH), 1.33 (s, 3H, C**H**$_3$C(OH)).

^{13}C NMR (125 MHz, CDCl$_3$, 298 K) δ/ppm: 156.9 (dt, 1C, $^{1}J_{CF}$ = 263.1 Hz, $^{2}J_{CF}$ = $^{2}J_{CF}$ = 33.6 Hz, **C**F=CH$_2$), 132.5 (s, 1C, **C**=CH), 123.6 (s, 1C, C=**C**H), 117.6 (td, 1C, $^{1}J_{CF}$ = $^{1}J_{CF}$ = 251.0 Hz, $^{2}J_{CF}$ = 33.0 Hz, **C**F$_2$), 96.3 (dt, $^{2}J_{CF}$ = 14.0 Hz, $^{3}J_{CF}$ = 4.3 Hz, CF=**C**H$_2$), 74.8 (t, $^{2}J_{CF}$ = $^{2}J_{CF}$ = 32.8 Hz, **C**CH$_3$(OH)), 35.2 (s, 1C, CH$_3$), 25.7 (s, 1C, CH$_3$), 21.5 (s, 1C, CH$_2$), 20.1 (d, 1C, $^{3}J_{CF}$ = 3.2 Hz, **C**H$_3$C(OH)), 17.6 (s, 1C, CH$_2$).

^{19}F NMR (376 MHz, CDCl$_3$, 298 K) δ/ppm: −111.89 (ddt, 1F, $^{3}J_{HF}$ = 46.3 Hz, $^{3}J_{HF}$ = 15.4 Hz, $^{3}J_{FF}$ = 12.3 Hz, C**F**=CH$_2$), −116.55 (d, 2F, $^{3}J_{FF}$ = 12.3 Hz, C**F**$_2$).

7.1 X-ray data for compounds **5a**, and **5j**.

Table S8 provides a summary of the crystallographic data for the structures of **5a**, and **5j**. Data were collected using an Agilent Xcalibur PX Ultra A diffractometer, and the structures were solved and refined using the OLEX2[10], SHELXTL[11] and SHELX-2013[12] program systems.

Table S8: Crystal data, data collection and refinement parameters for the structures of **5a**, and **5j**.

Compound no.	**5a**	**5j**
CCDC no.	2380550	2380551
formula	$C_{11}H_{11}O_2F_3$	$C_{17}H_{16}NO_2F_3S$
formula weight	232.20	355.37
colour, habit	Colourless, block	Colourless, block
temperature/K	173(3)	172.9(3)
crystal system	triclinic	monoclinic
space group	P-1	$P2_1/c$
a / Å	8.8525(6)	9.1840(2)
b / Å	9.5403(5)	22.1275(5)
c / Å	14.4085(7)	8.4351(2)
α / deg	87.615(4)	90
β / deg	72.239(5)	107.751(3)
γ / deg	66.659(6)	90
V / $Å^3$	1059.66(12)	1632.56(7)
Z	4	4
D_c / g cm^{-3}	1.455	1.446
radiation used	Cu Kα (λ = 1.54184)	Cu Kα (λ = 1.54184)
μ / mm^{-1}	1.169	2.152
no. of unique reflns:		
measured (R_{int})	4210 (0.0465)	3230 (0.0397)
obs, $\|F_o\| > 4\sigma(\|F_o\|)$	3120	2683
completeness (%) [a]	100	99.8
no. of variables	293	221
R_1(obs), wR_2(all) [b]	0.0542, 0.1659	0.0406, 0.1068

Crystal structure of **5a**

Compound **5a** was crystallised from a racemic mixture and found to crystallise in the triclinic space group P-1. No enantiomeric enrichment was observed during crystallisation and due to the absence of a mirror plane there are two independent molecules (one R- and one S-enantiomer) per unit cell (Z’ = 2).

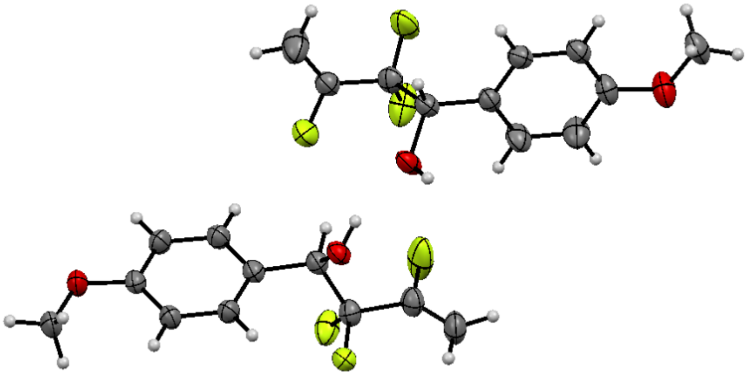

Figure S15: Crystal structure of **5a**, thermal ellipsoids are at the 50 % probability level.

Crystal structure of **5j**

Compound **5j** was crystallised from a racemic mixture and found to crystallise in the monoclinic space group P21/c. No enantiomeric enrichment was observed during crystallisation.

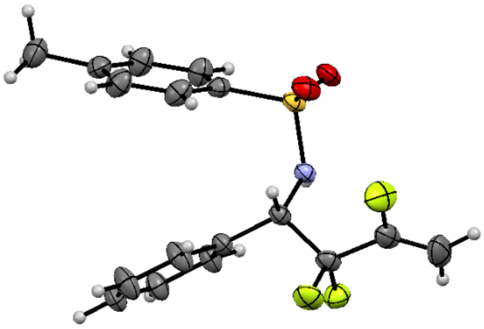

Figure S16: Crystal structure of **5j**, thermal ellipsoids are at the 50 % probability level.

8.1 NMR spectra

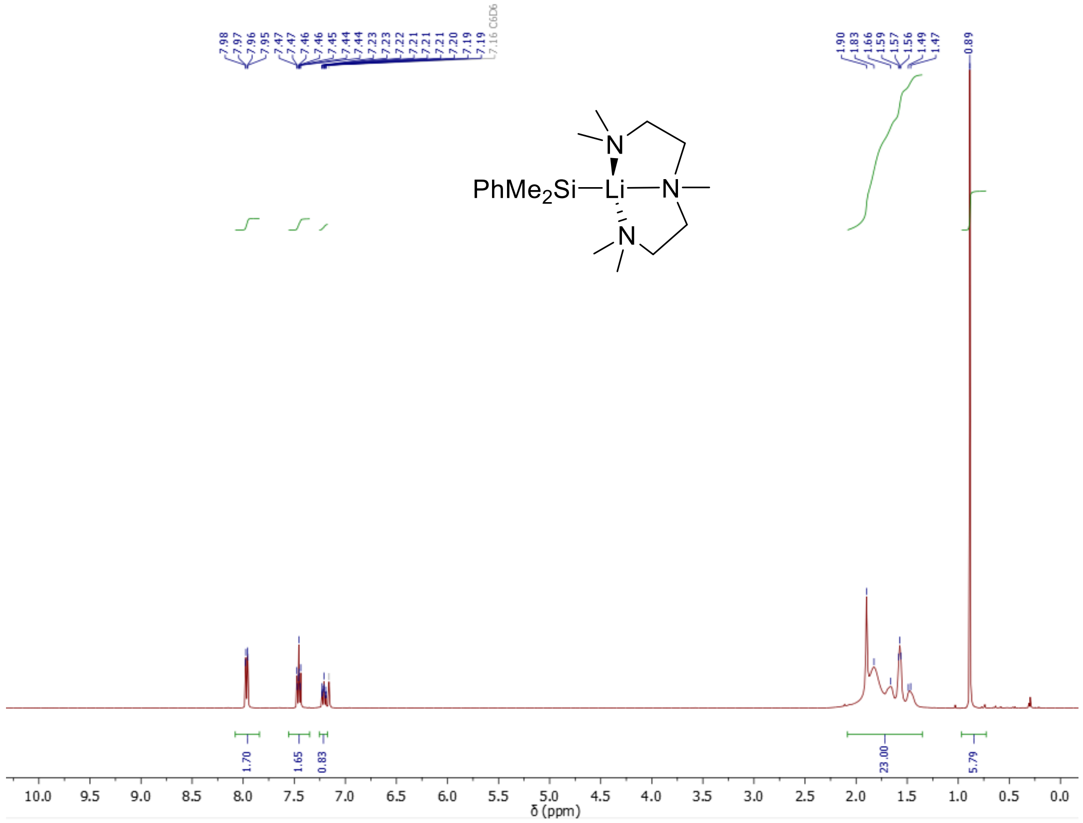

^{1}H NMR (400 MHz, C_6D_6) spectra of dimethylphenylsilyl lithium PMDETA complex, **1•PMDETA**

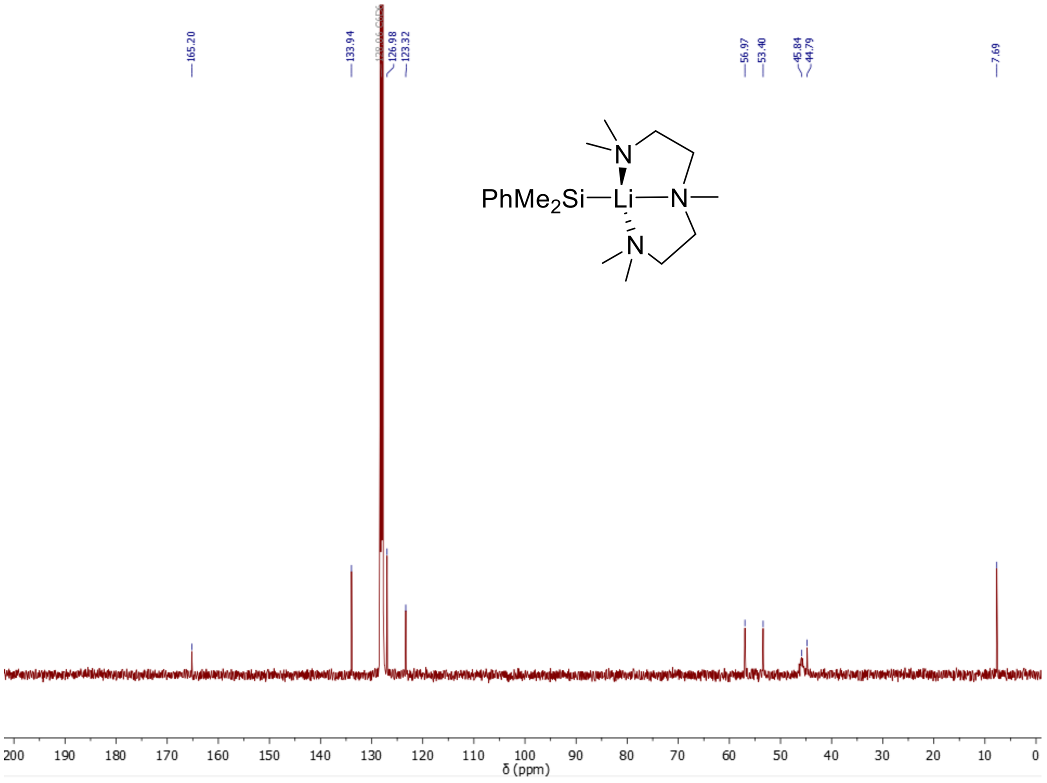

^{13}C NMR (100 MHz, C_6D_6) spectra of dimethylphenylsilyl lithium PMDETA complex, **1•PMDETA**

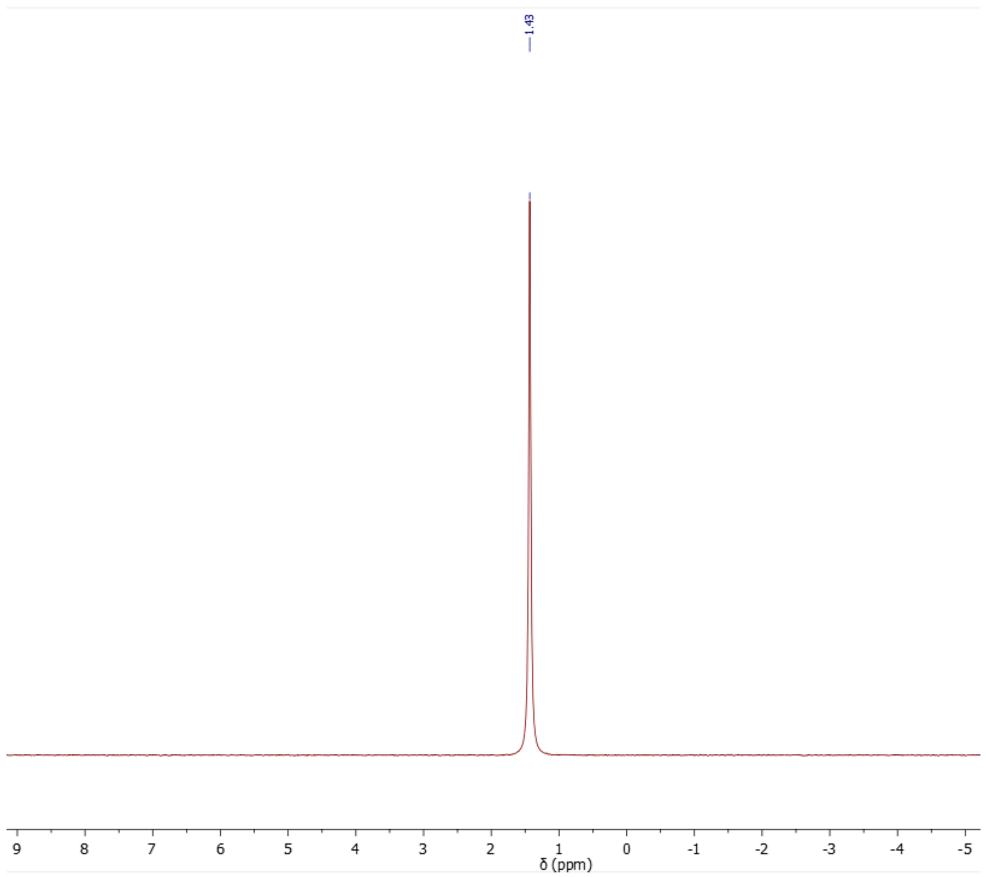

^{7}Li NMR (194 MHz, C_6D_6) spectra of dimethylphenylsilyl lithium PMDETA complex, **1•PMDETA**

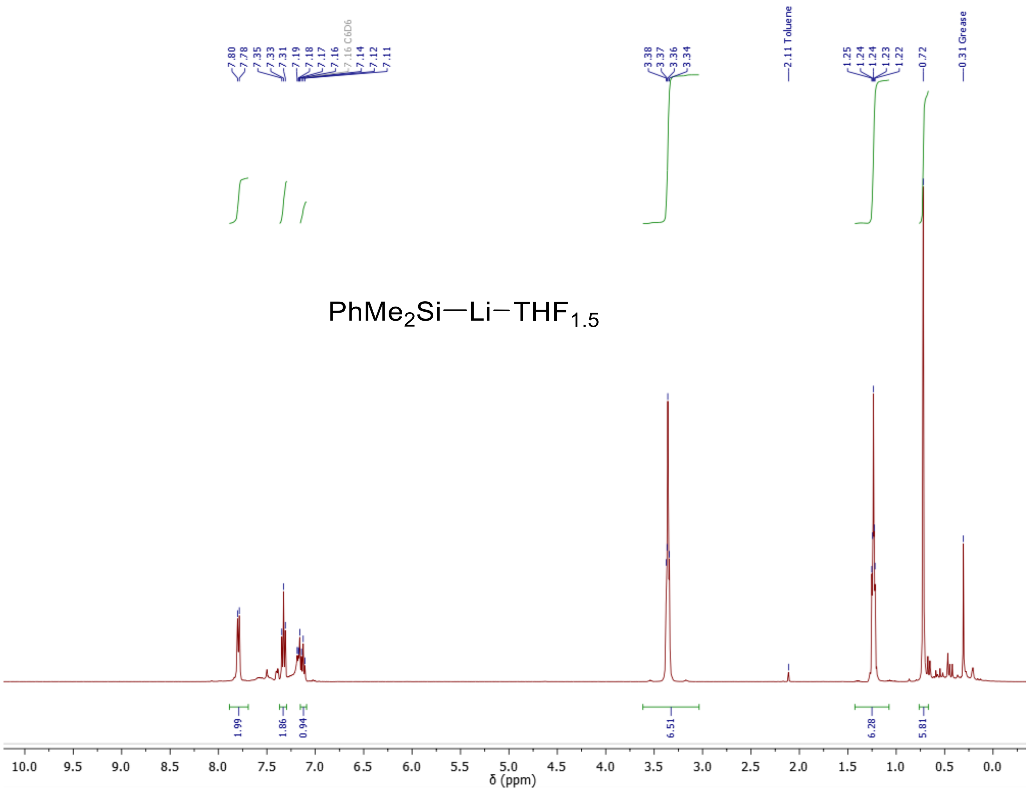

^{1}H NMR (400 MHz, C_6D_6) spectra of dimethylphenylsilyl lithium THF complex, **1•THF**

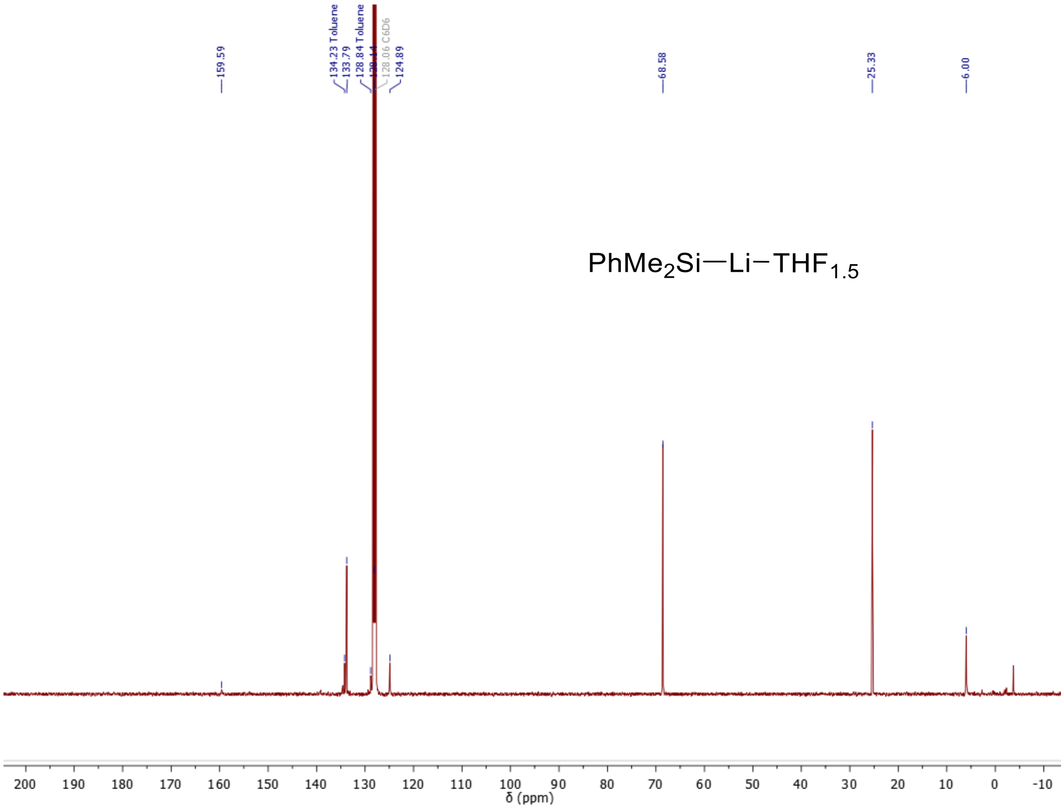

^{13}C NMR (100 MHz, C_6D_6) spectra of dimethylphenylsilyl lithium THF complex, **1•THF**

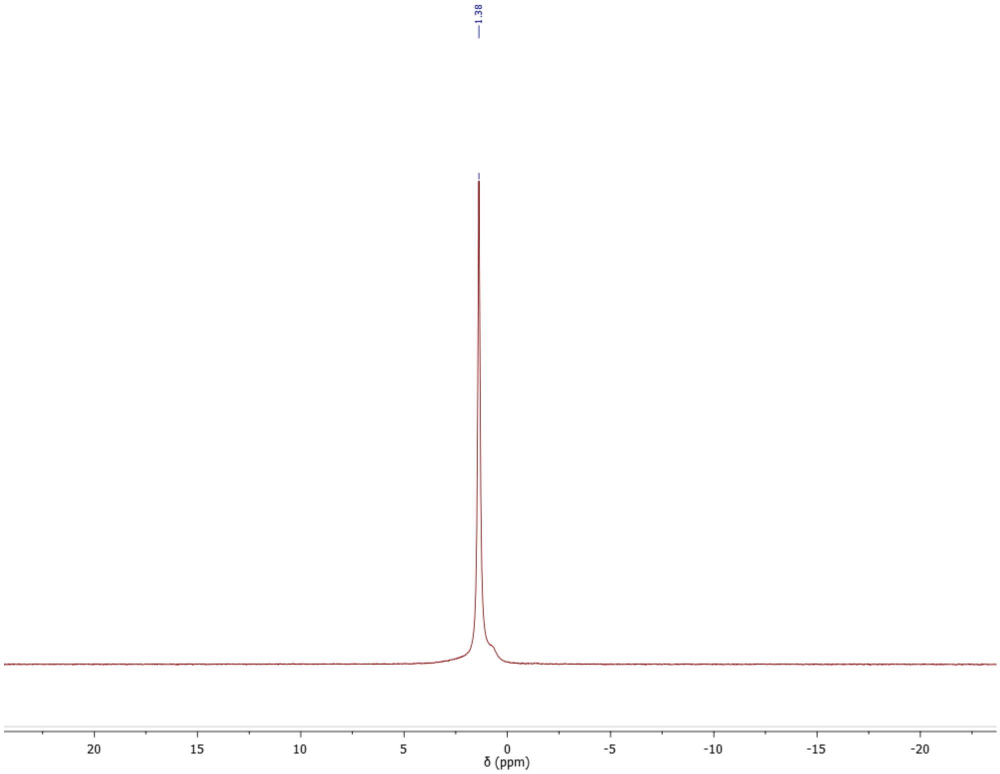

^{7}Li NMR (194 MHz, C_6D_6) spectra of dimethylphenylsilyl lithium THF complex, **1•THF**

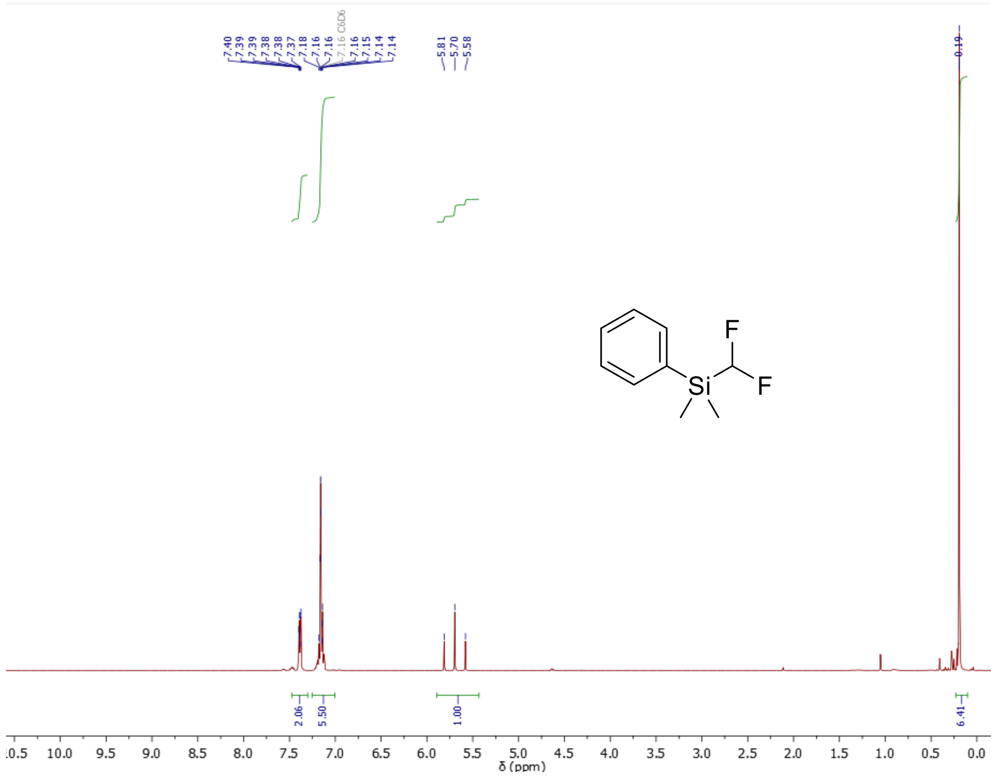

^{1}H NMR (400 MHz, C_6D_6) spectra of (difluoromethyl)dimethylphenylsilane, **2**

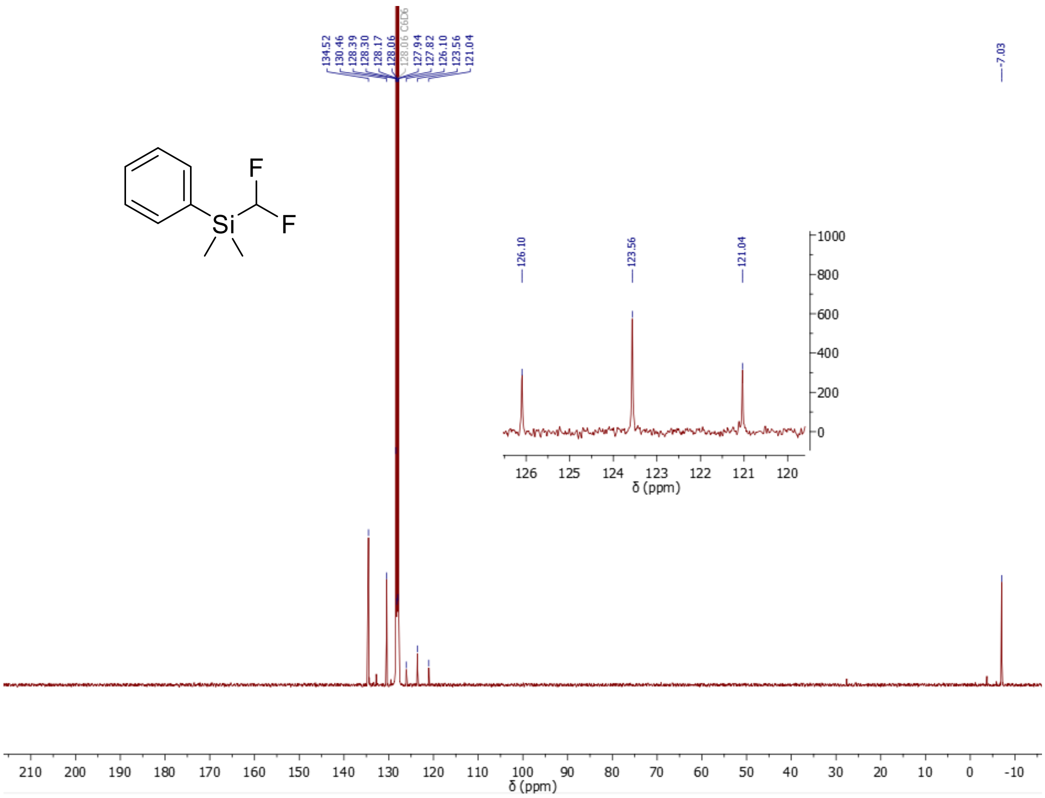

^{13}C NMR (100 MHz, C_6D_6) spectra of (difluoromethyl)dimethylphenylsilane, **2**

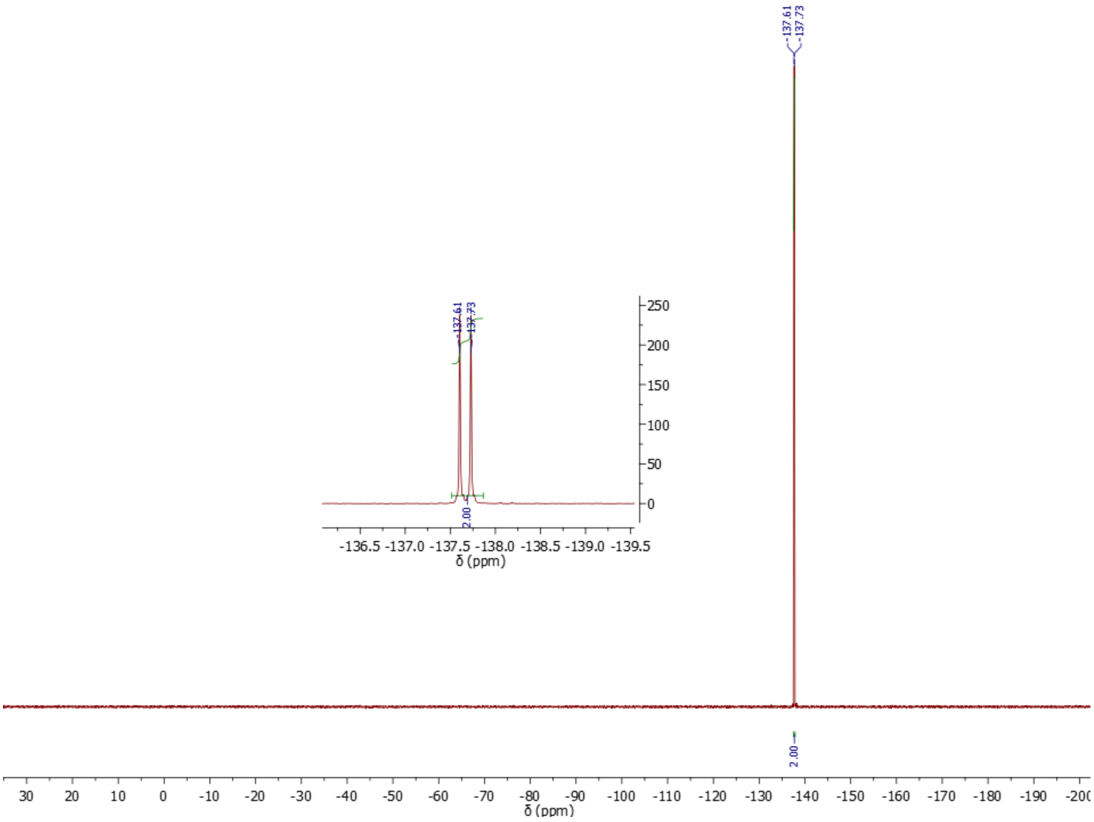

^{19}F NMR (376 MHz, C_6D_6) spectra of (difluoromethyl)dimethylphenylsilane, **2**

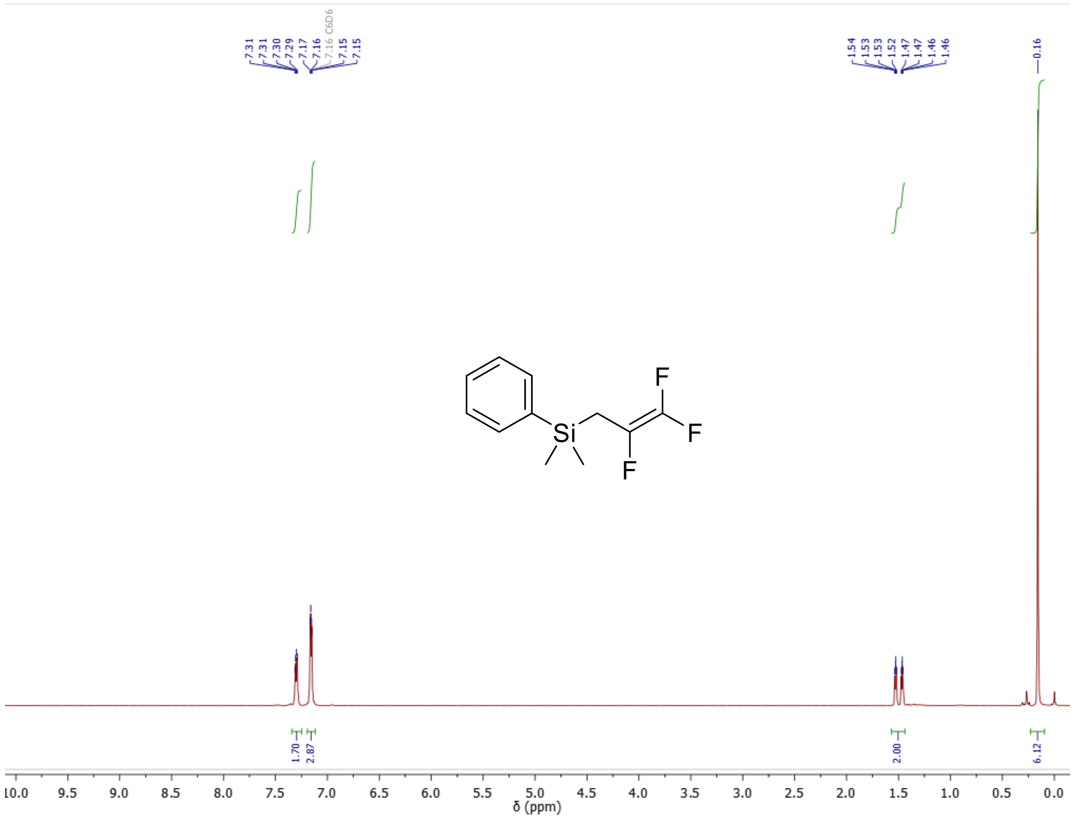

^{1}H NMR (400 MHz, C_6D_6) spectra of dimethyl(phenyl)(2,3,3-trifluoroallyl)silane, **3**

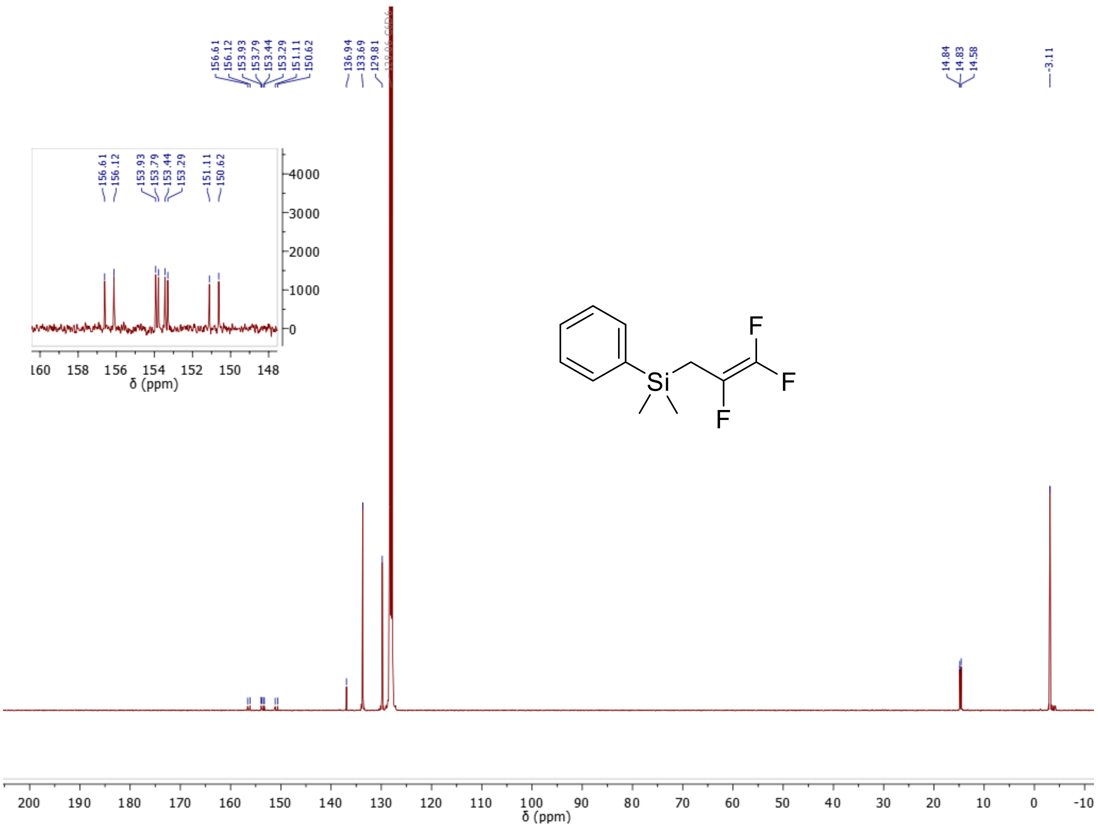

^{13}C NMR (100 MHz, C_6D_6) spectra of dimethyl(phenyl)(2,3,3-trifluoroallyl)silane, **3**

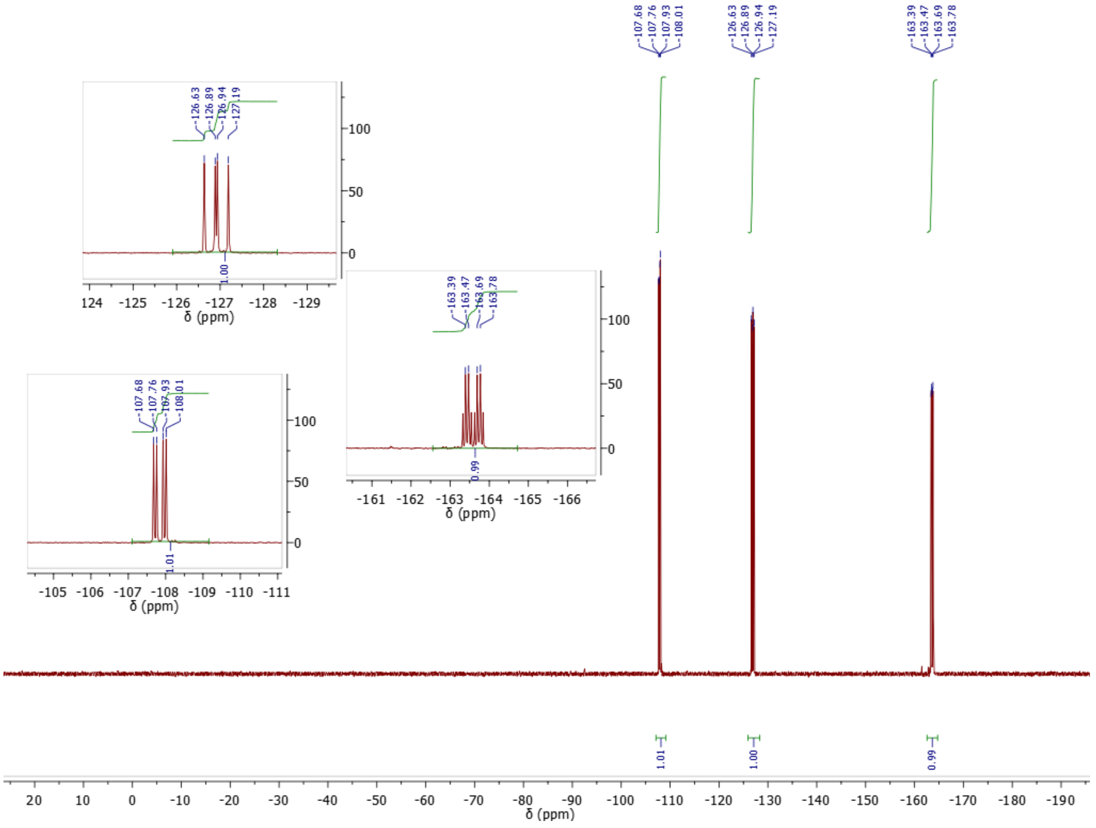

^{19}F NMR (376 MHz, C_6D_6) spectra of dimethyl(phenyl)(2,3,3-trifluoroallyl)silane, **3**

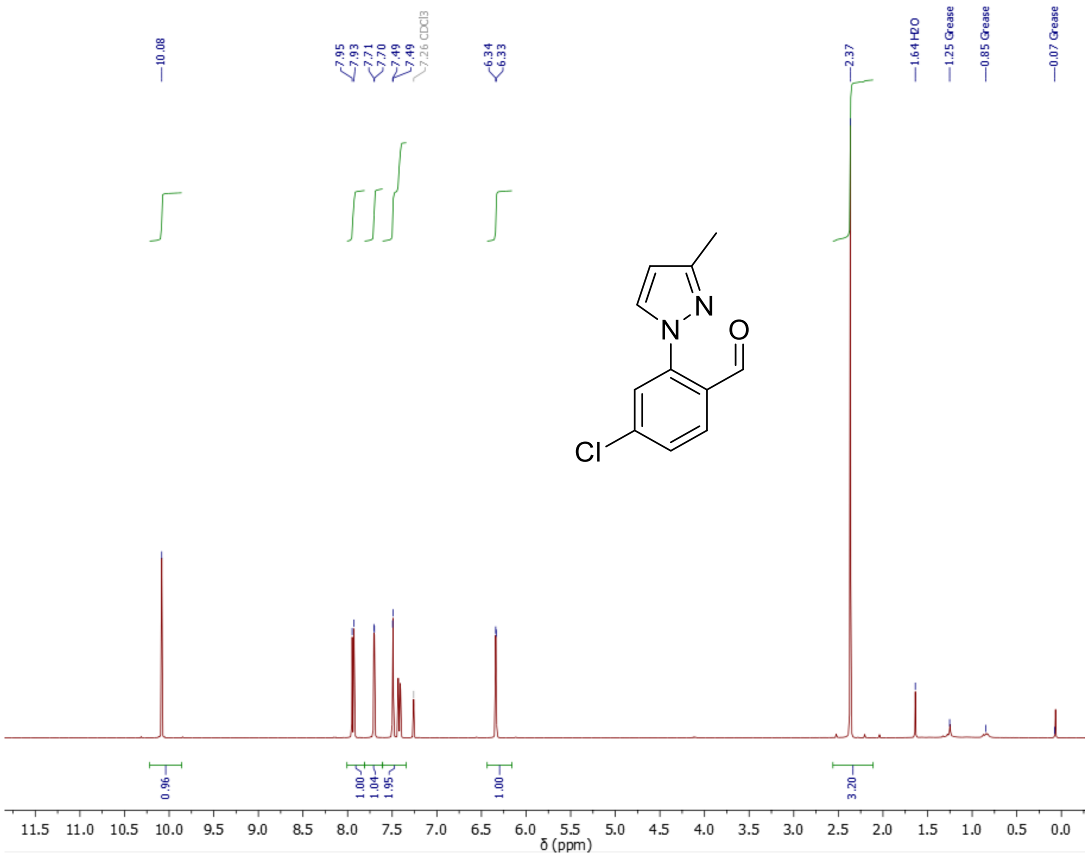

^{1}H NMR (400 MHz, $CDCl_3$) spectra of 4-chloro-2-(3-methyl-1H-pyrazol-1-yl)benzaldehyde

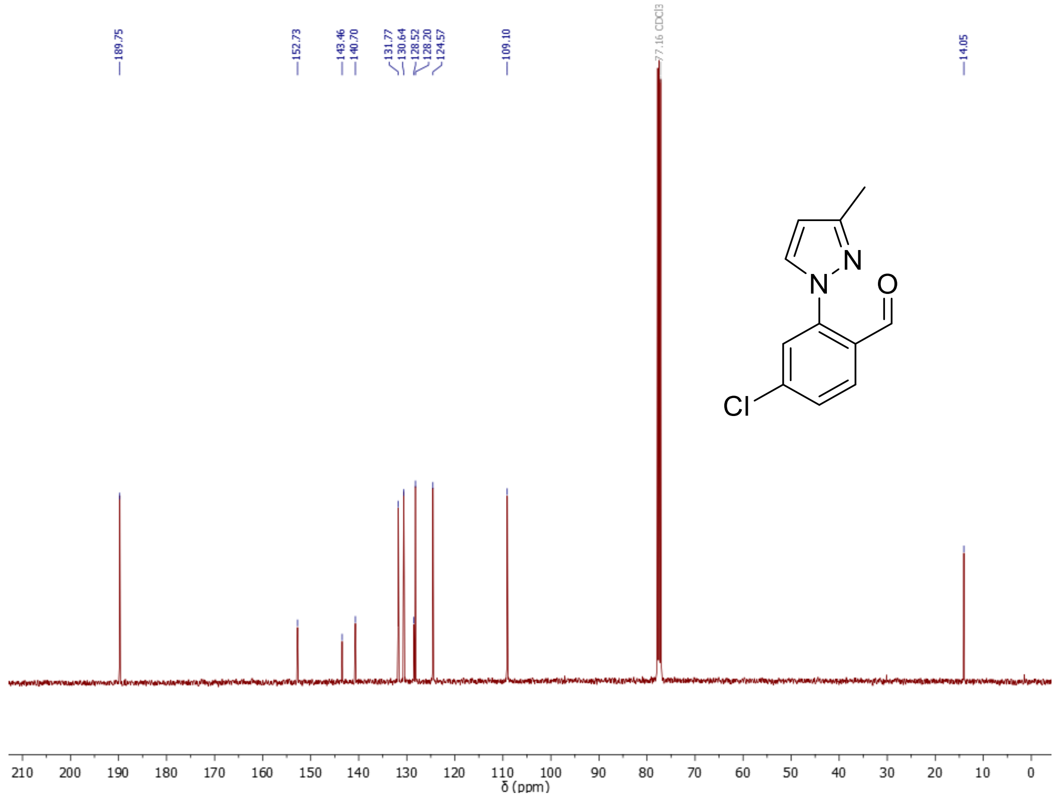

^{13}C NMR (100 MHz, $CDCl_3$) spectra of 4-chloro-2-(3-methyl-1H-pyrazol-1-yl)benzaldehyde

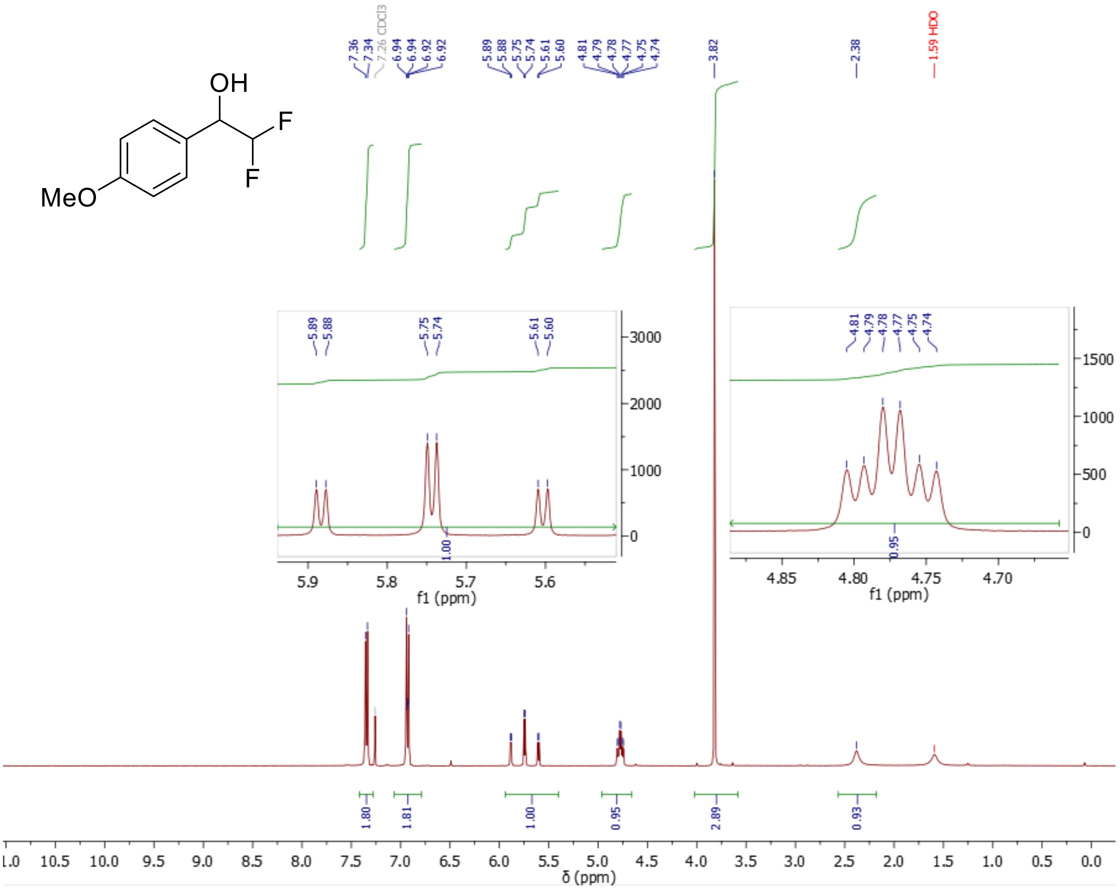

^{1}H NMR (400 MHz, CDCl₃) spectra of 2,2-difluoro-1-(4-methoxyphenyl)ethan-1-ol, **4a**

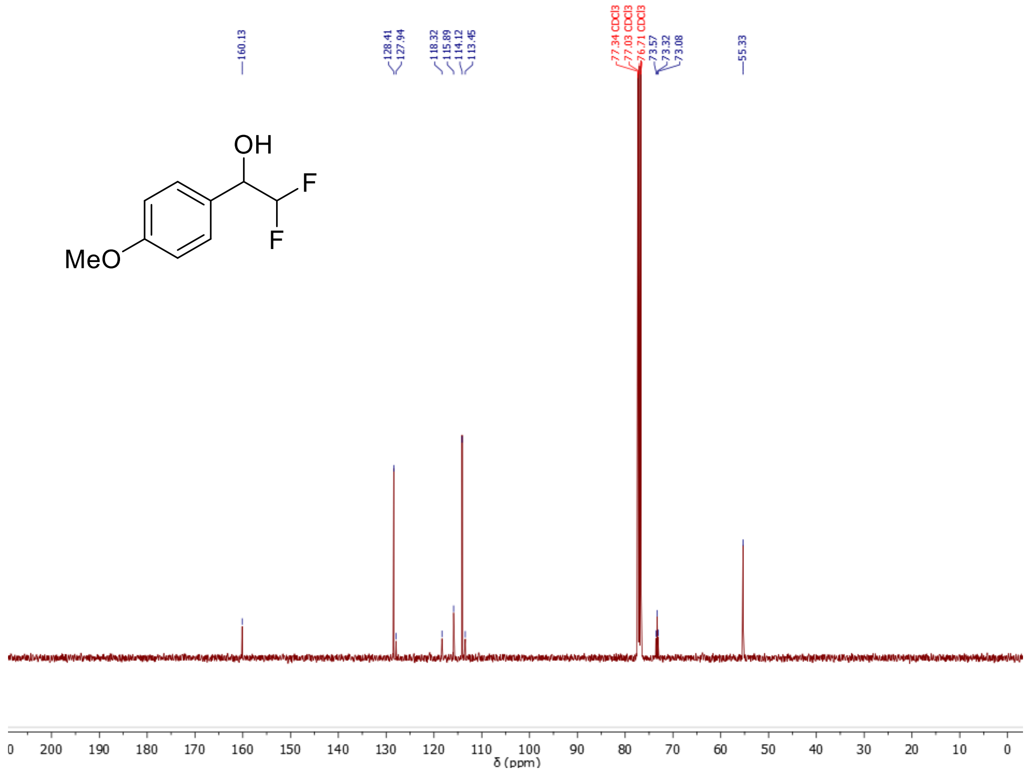

^{13}C NMR (100 MHz, CDCl₃) spectra of 2,2-difluoro-1-(4-methoxyphenyl)ethan-1-ol, **4a**

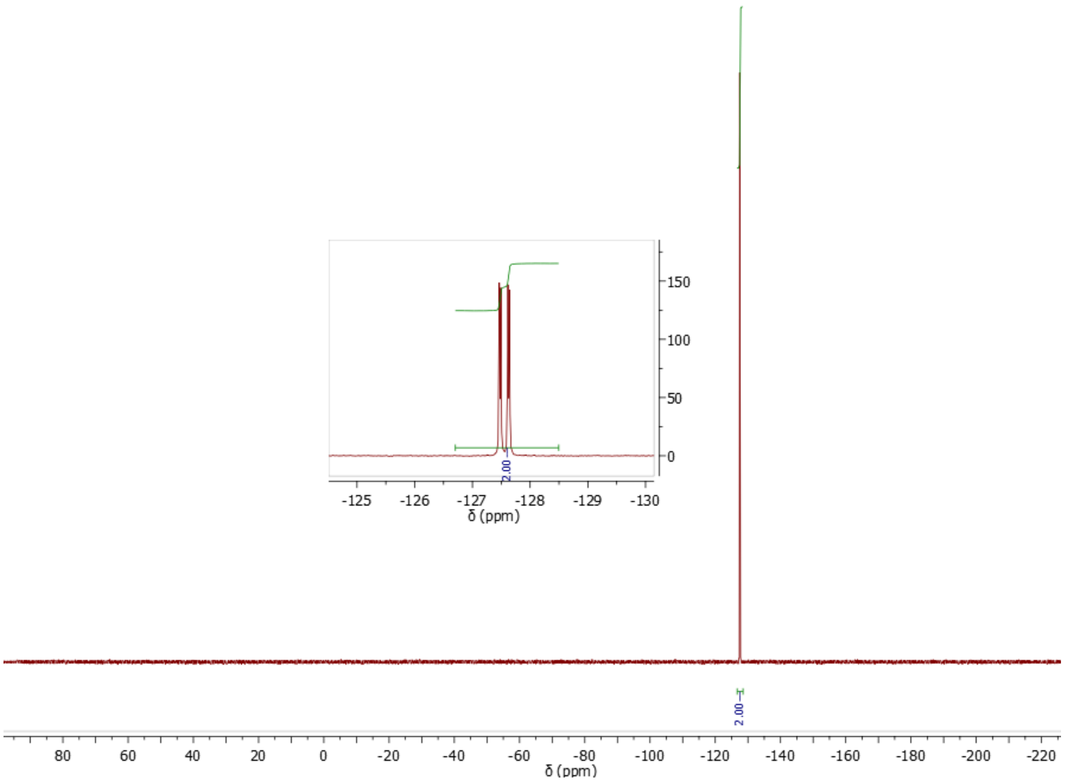

^{19}F NMR (376 MHz, $CDCl_3$) spectra of 2,2-difluoro-1-(4-methoxyphenyl)ethan-1-ol, **4a**

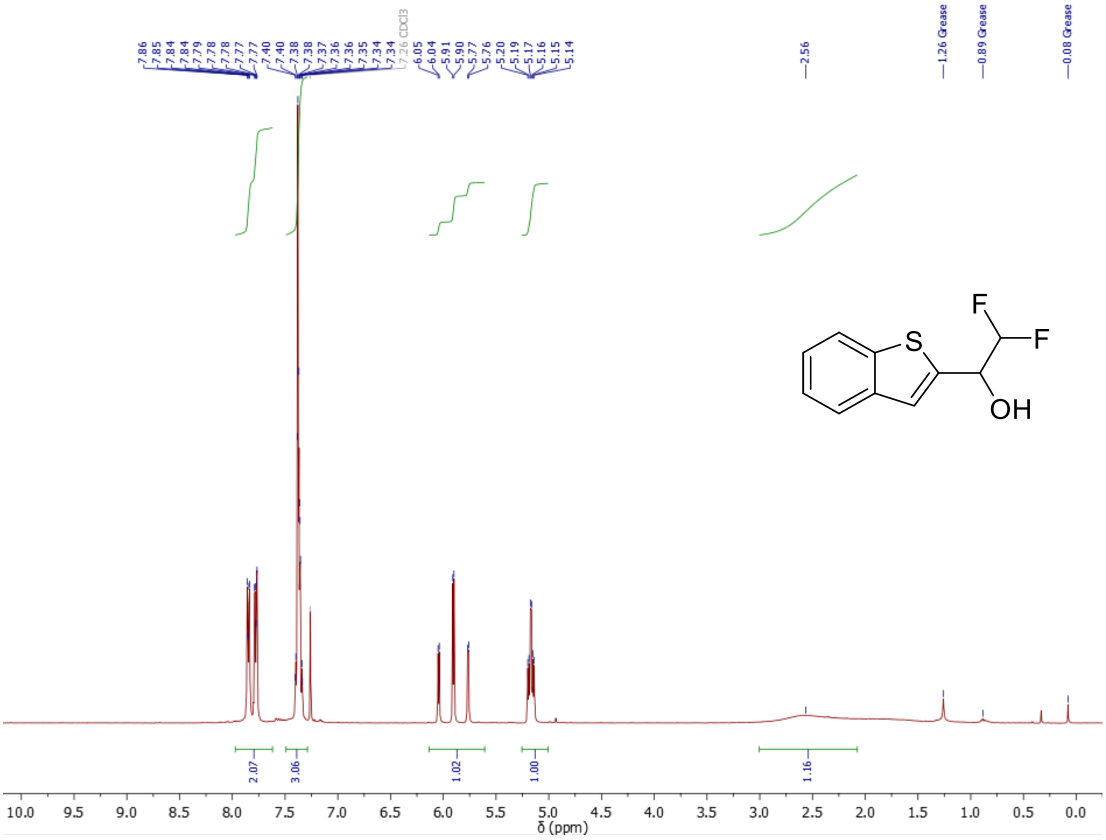

^{1}H NMR (400 MHz, $CDCl_3$) spectra of 1-(benzo[b]thiophen-2-yl)-2,2-difluoroethan-1-ol, 4**b**

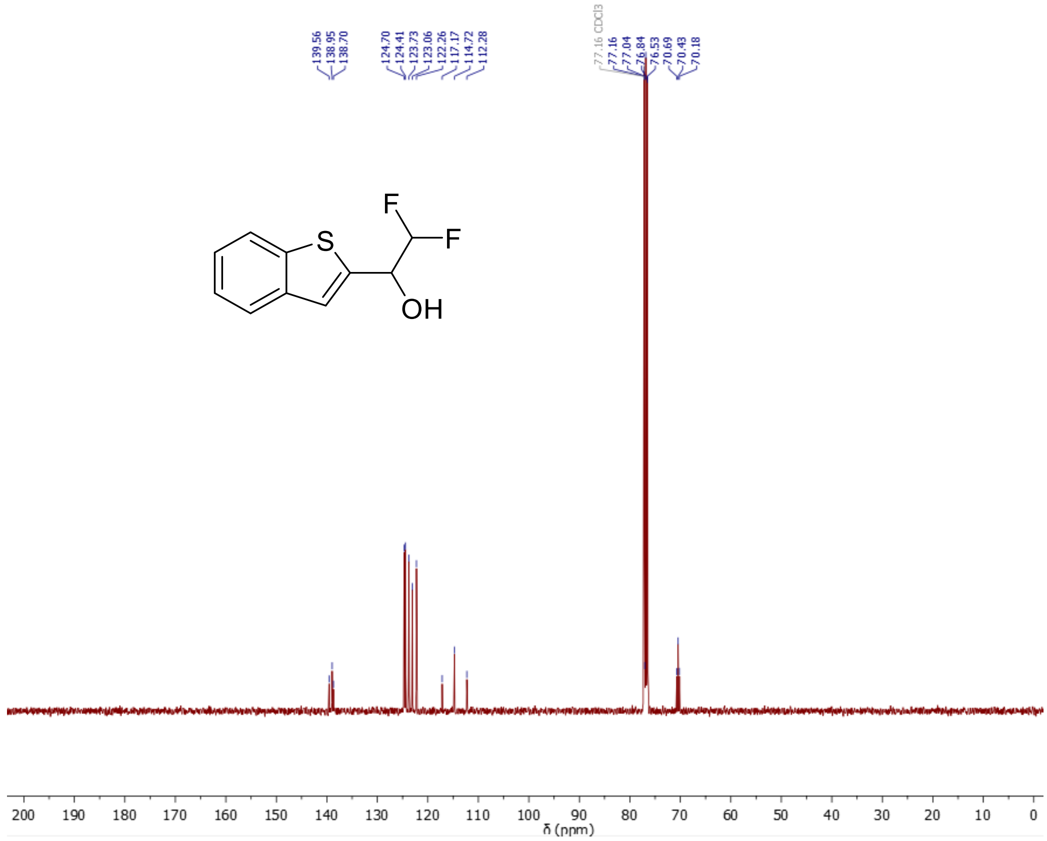

^{13}C NMR (100 MHz, $CDCl_3$) spectra of 1-(benzo[b]thiophen-2-yl)-2,2-difluoroethan-1-ol, **4b**

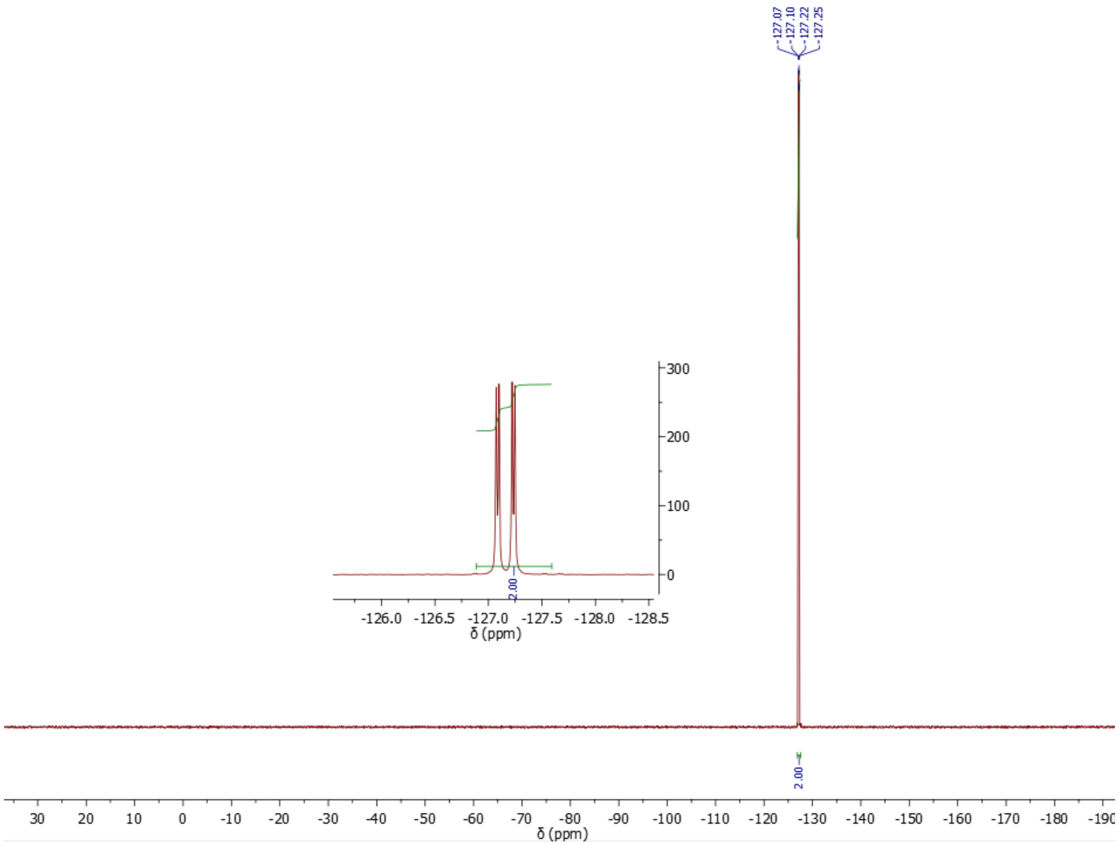

^{19}F NMR (376 MHz, $CDCl_3$) spectra of 1-(benzo[b]thiophen-2-yl)-2,2-difluoroethan-1-ol, **4b**

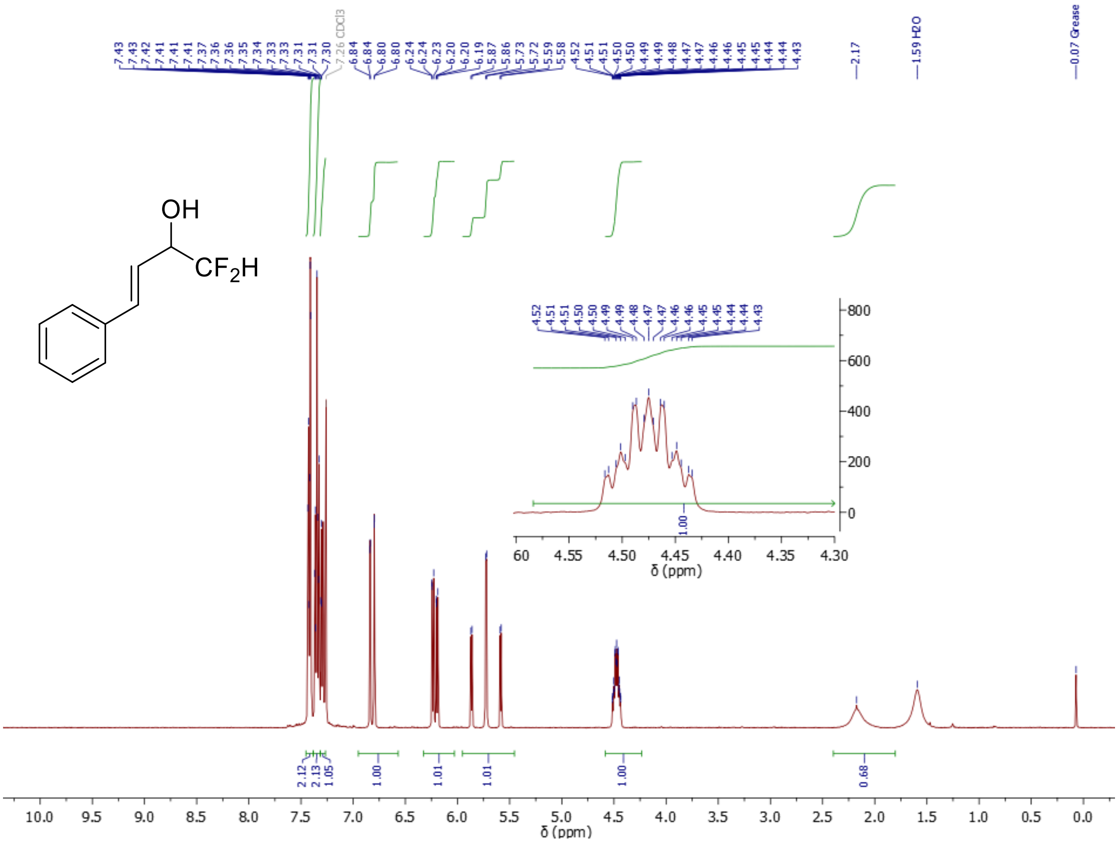

^{1}H NMR (400 MHz, $CDCl_3$) spectra of (E)-1,1-difluoro-4-phenylbut-3-en-2-ol, **4c**

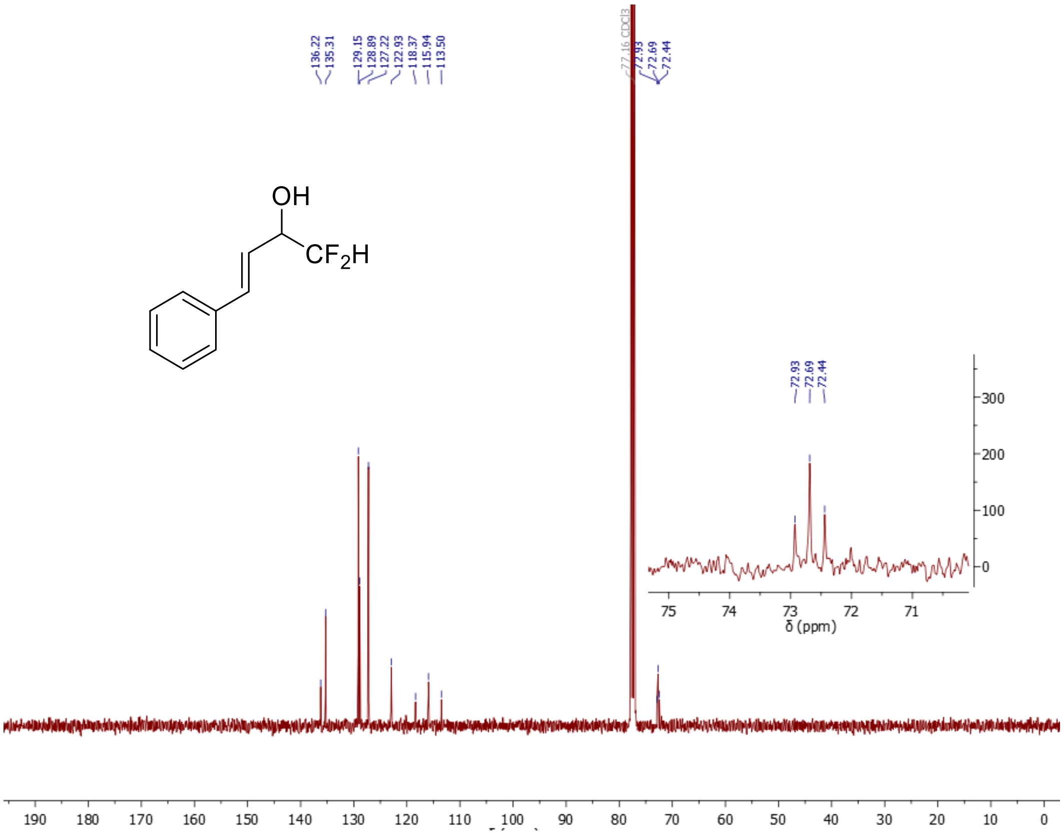

^{13}C NMR (100 MHz, $CDCl_3$) spectra of (E)-1,1-difluoro-4-phenylbut-3-en-2-ol, **4c**

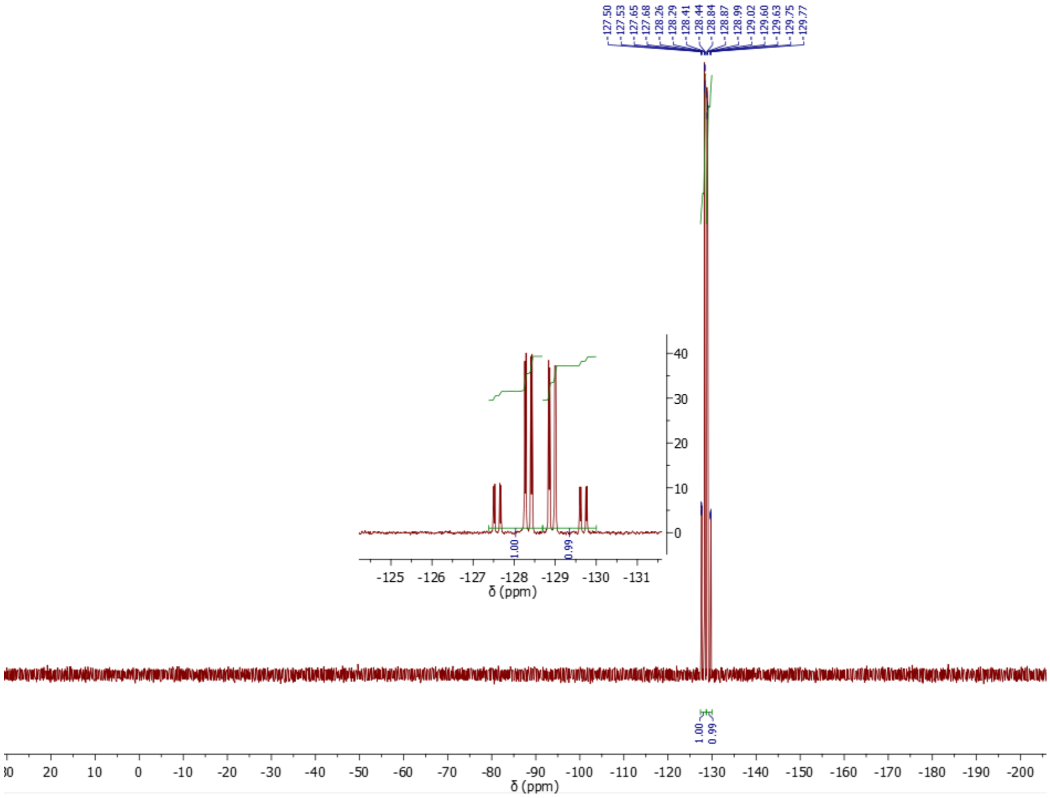

^{19}F NMR (376 MHz, $CDCl_3$) spectra of (E)-1,1-difluoro-4-phenylbut-3-en-2-ol, **4c**

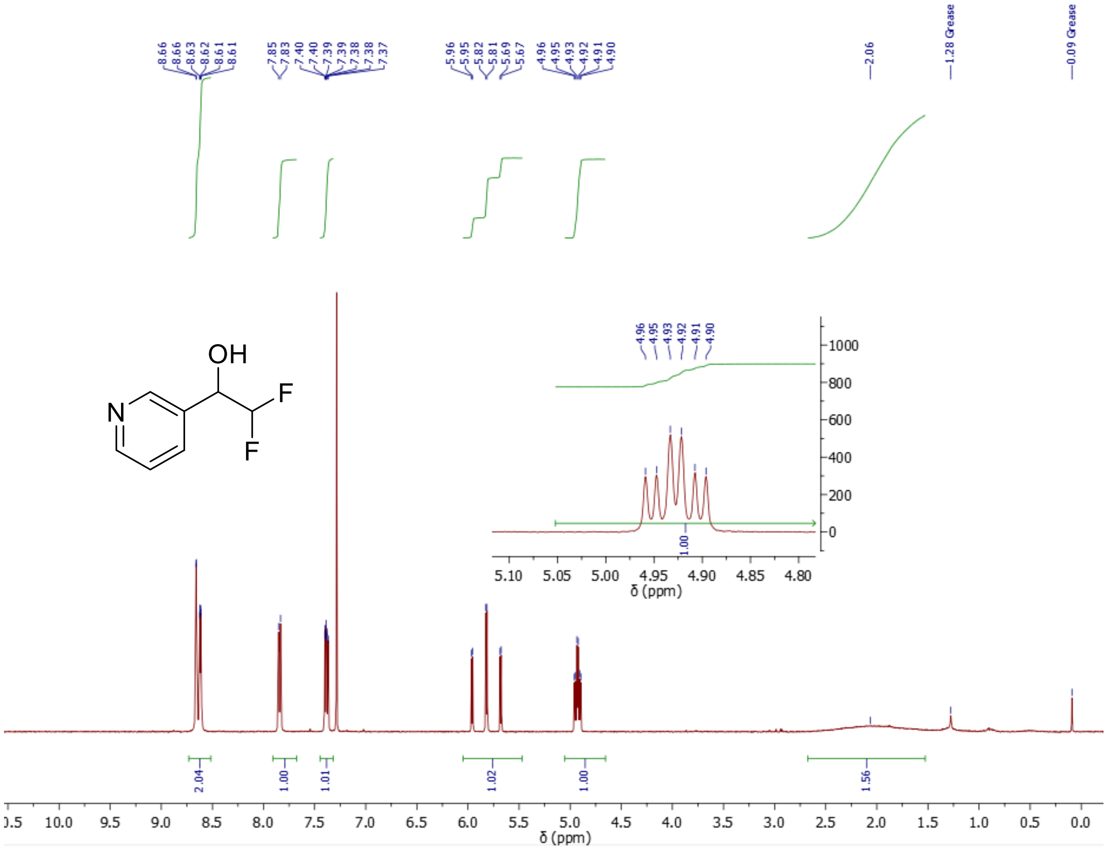

^{1}H NMR (400 MHz, $CDCl_3$) spectra of 2,2-difluoro-1-(pyridin-3-yl)ethan-1-ol, **4d**

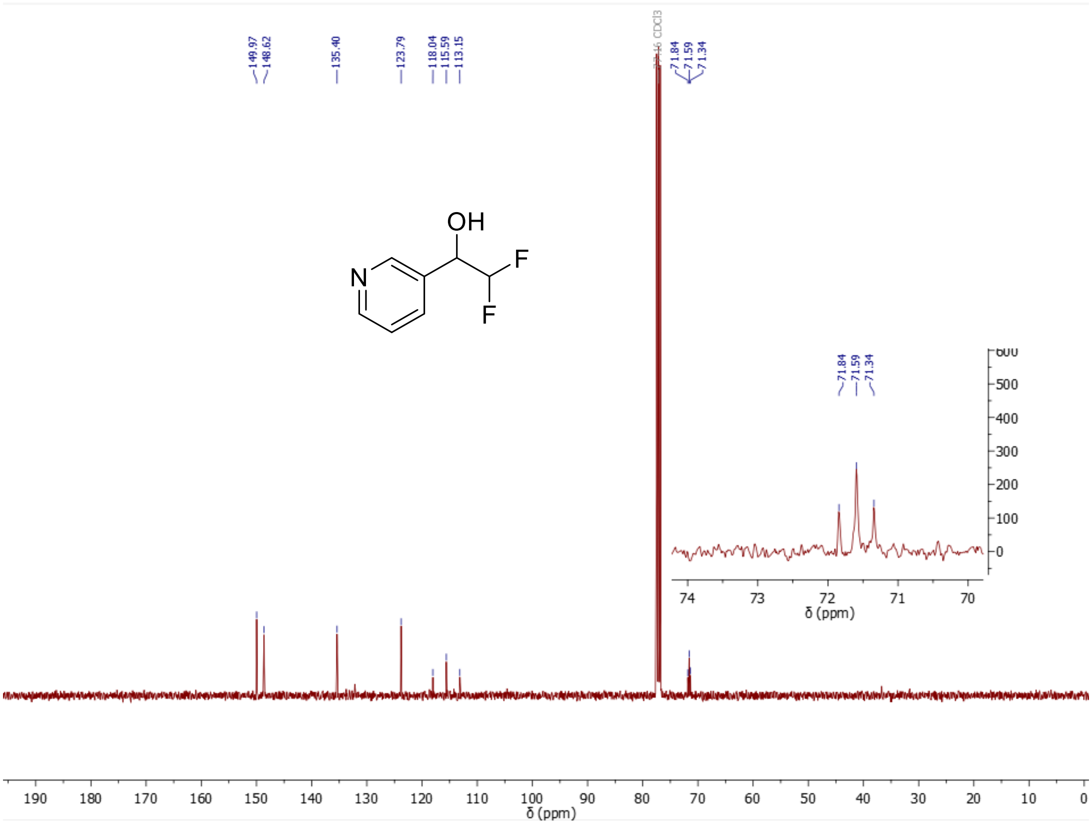

^{13}C NMR (100 MHz, $CDCl_3$) spectra of 2,2-difluoro-1-(pyridin-3-yl)ethan-1-ol, 4**d**

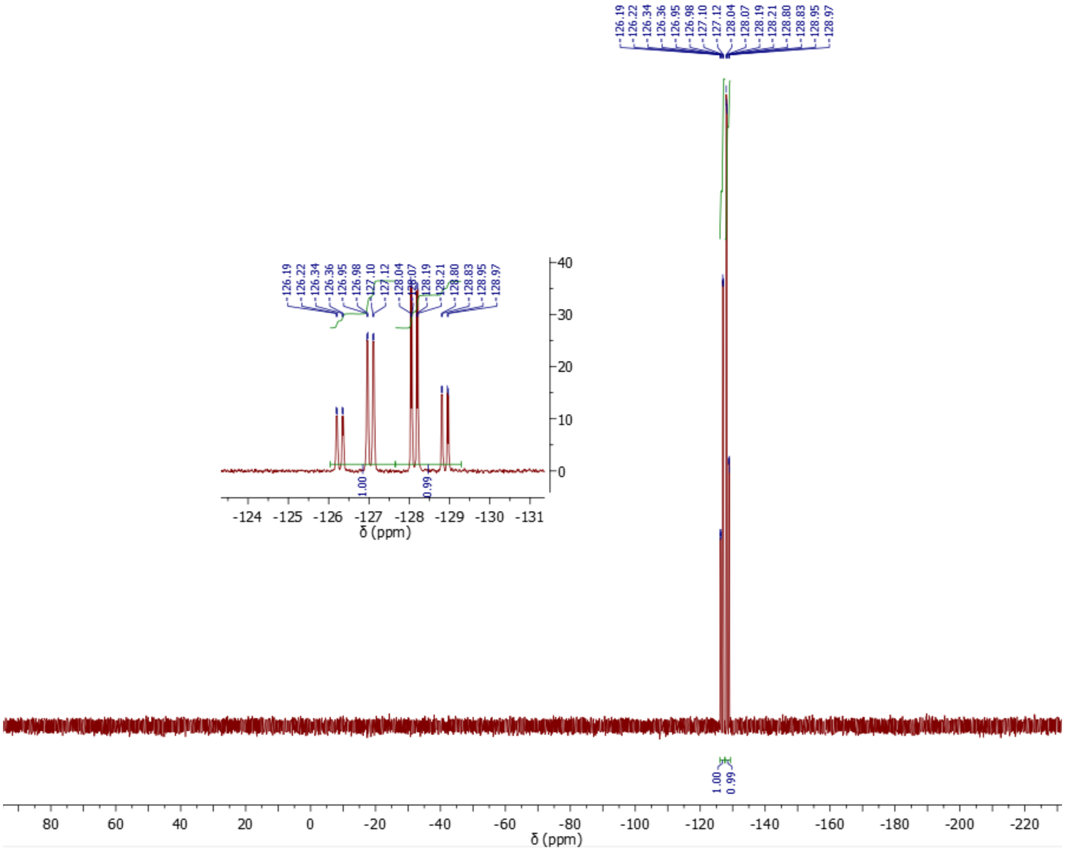

^{19}F NMR (376 MHz, $CDCl_3$) spectra of 2,2-difluoro-1-(pyridin-3-yl)ethan-1-ol, **4d**

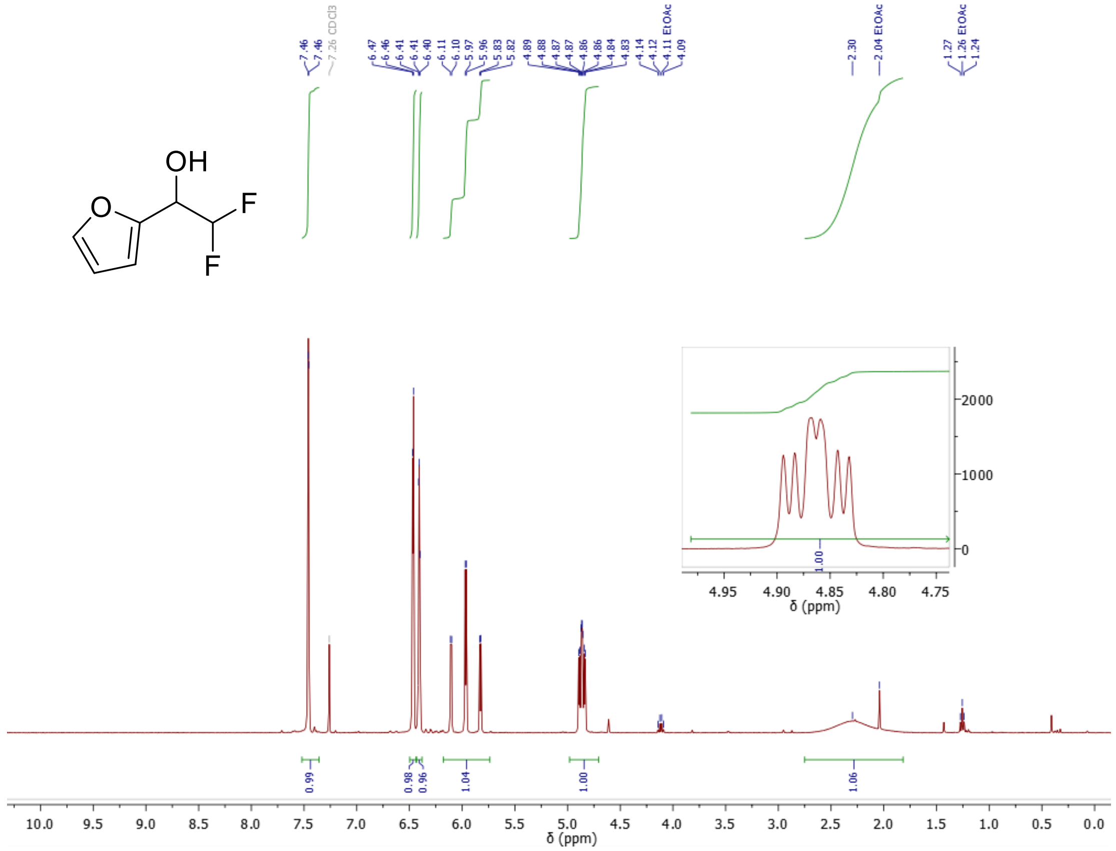

^{1}H NMR (400 MHz, $CDCl_3$) spectra of 2,2-difluoro-1-(furan-2-yl)ethan-1-ol, **4e**

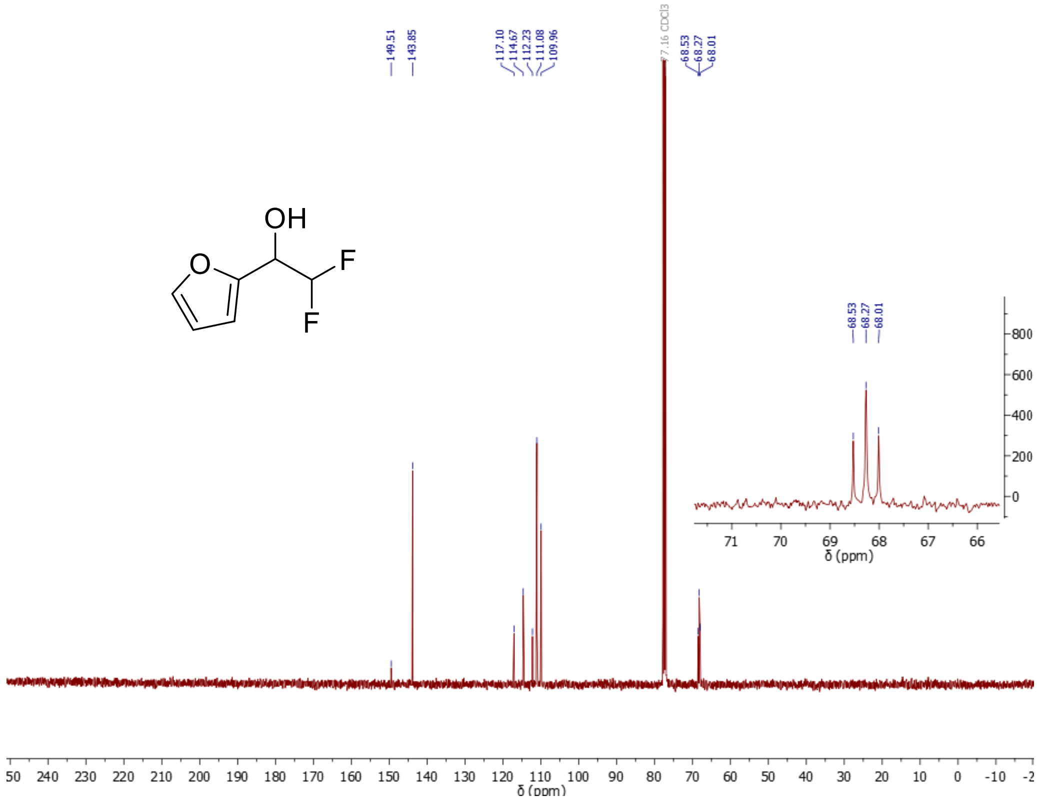

^{13}C NMR (100 MHz, $CDCl_3$) spectra of 2,2-difluoro-1-(furan-2-yl)ethan-1-ol, 4**e**

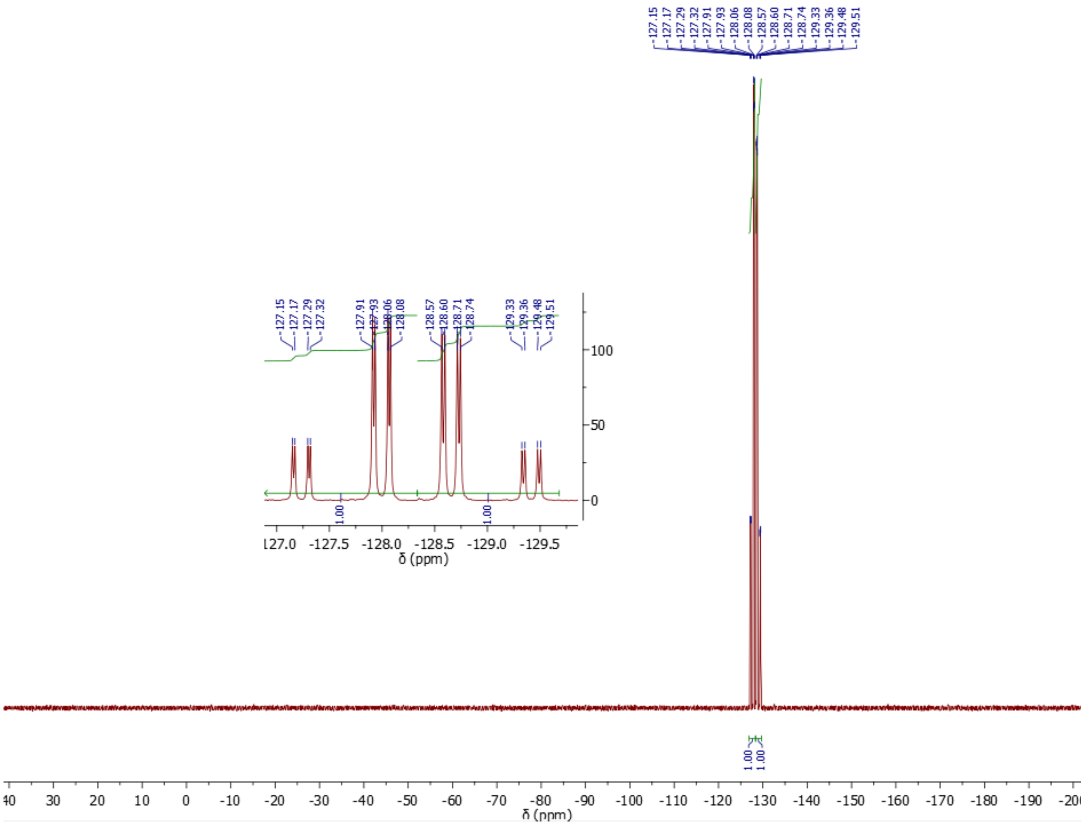

^{19}F NMR (376 MHz, $CDCl_3$) spectra of 2,2-difluoro-1-(furan-2-yl)ethan-1-ol, **4e**

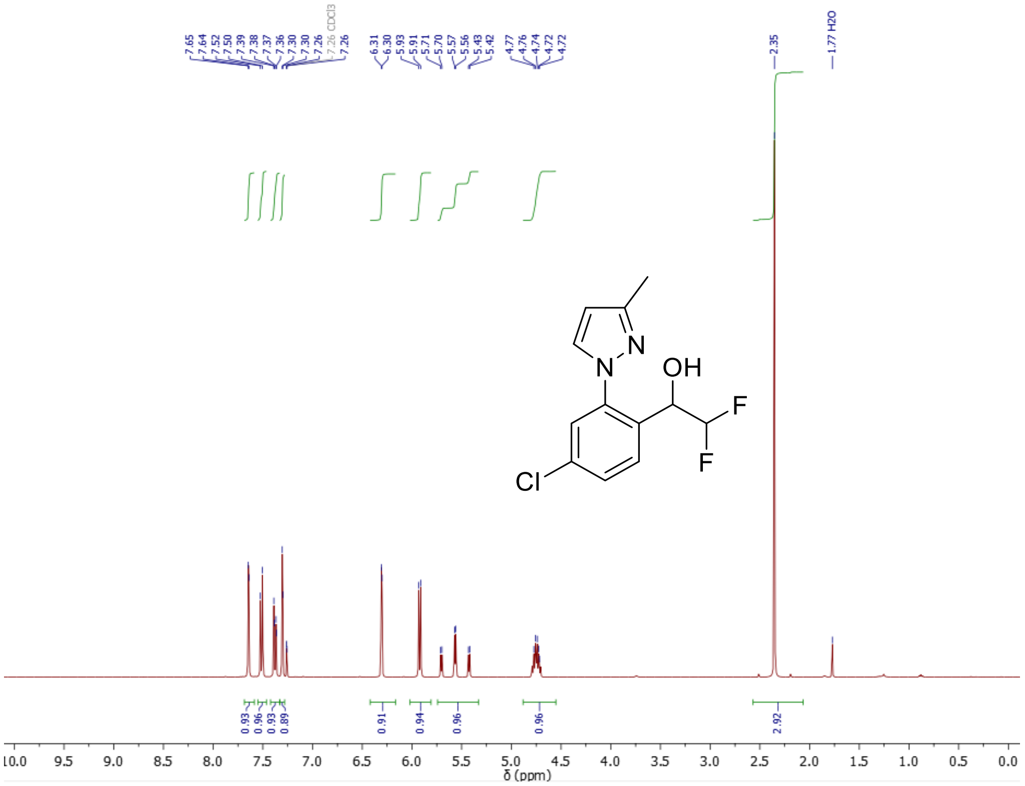

^{1}H NMR (400 MHz, $CDCl_3$) spectra of 1-(4-chloro-2-(3-methyl-1H-pyrazol-1-yl)phenyl)-2,2-difluoroethan-1-ol, **4f**

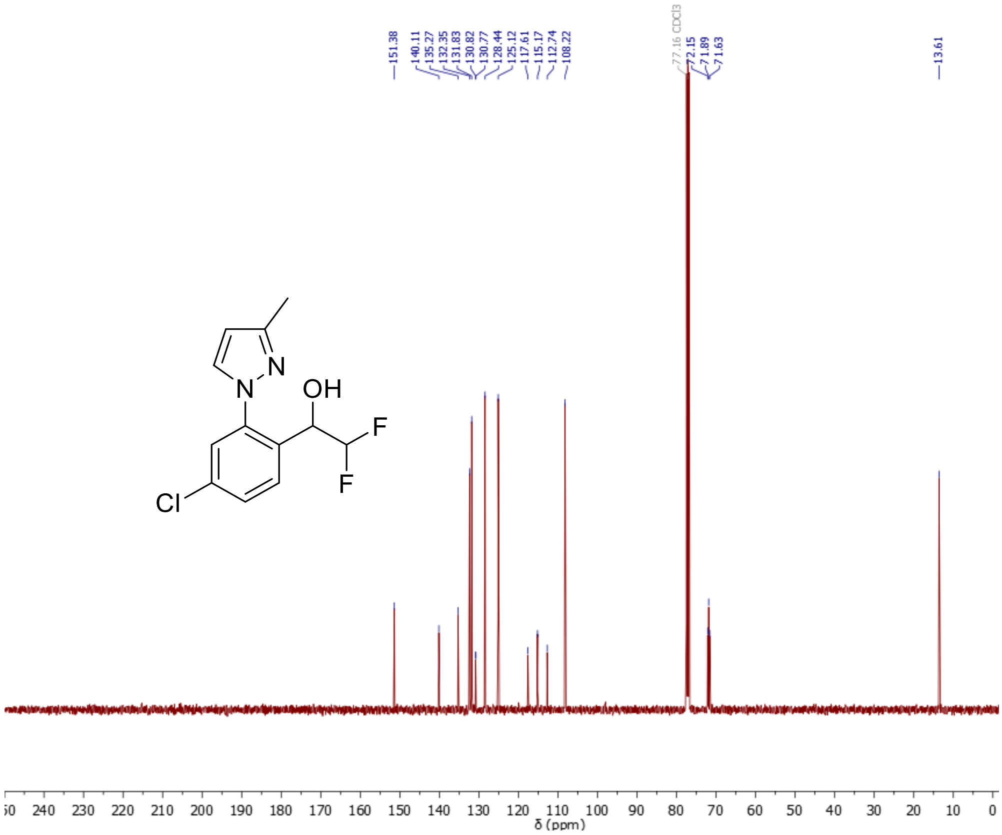

^{13}C NMR (100 MHz, $CDCl_3$) spectra of 1-(4-chloro-2-(3-methyl-1H-pyrazol-1-yl)phenyl)-2,2-difluoroethan-1-ol, 4**f**

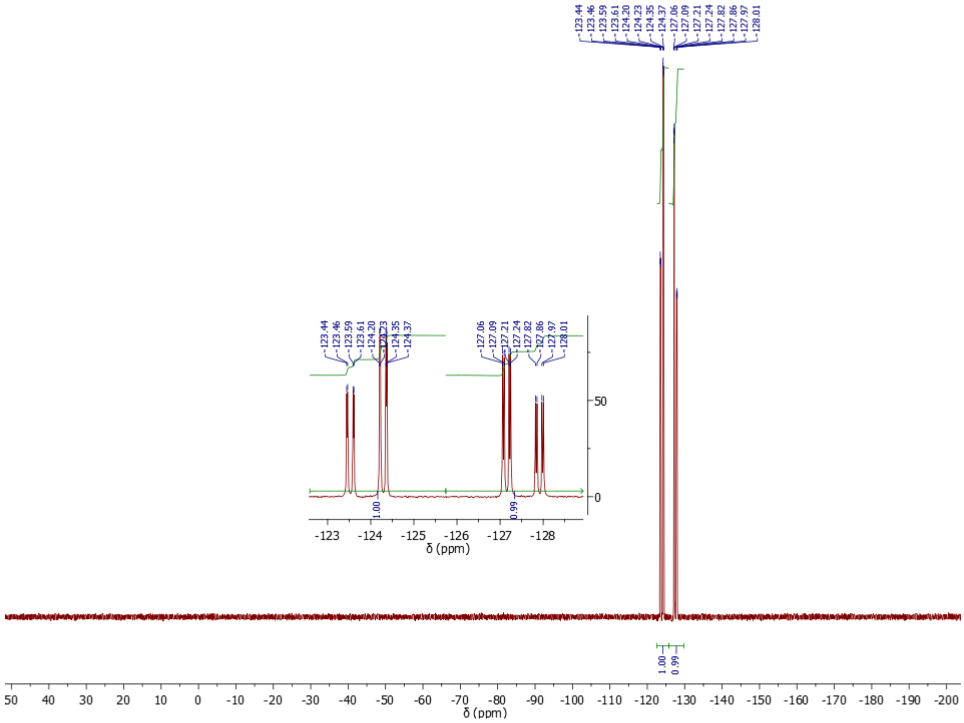

^{19}F NMR (376 MHz, $CDCl_3$) spectra of 1-(4-chloro-2-(3-methyl-1H-pyrazol-1-yl)phenyl)-2,2-difluoroethan-1-ol, **4f**

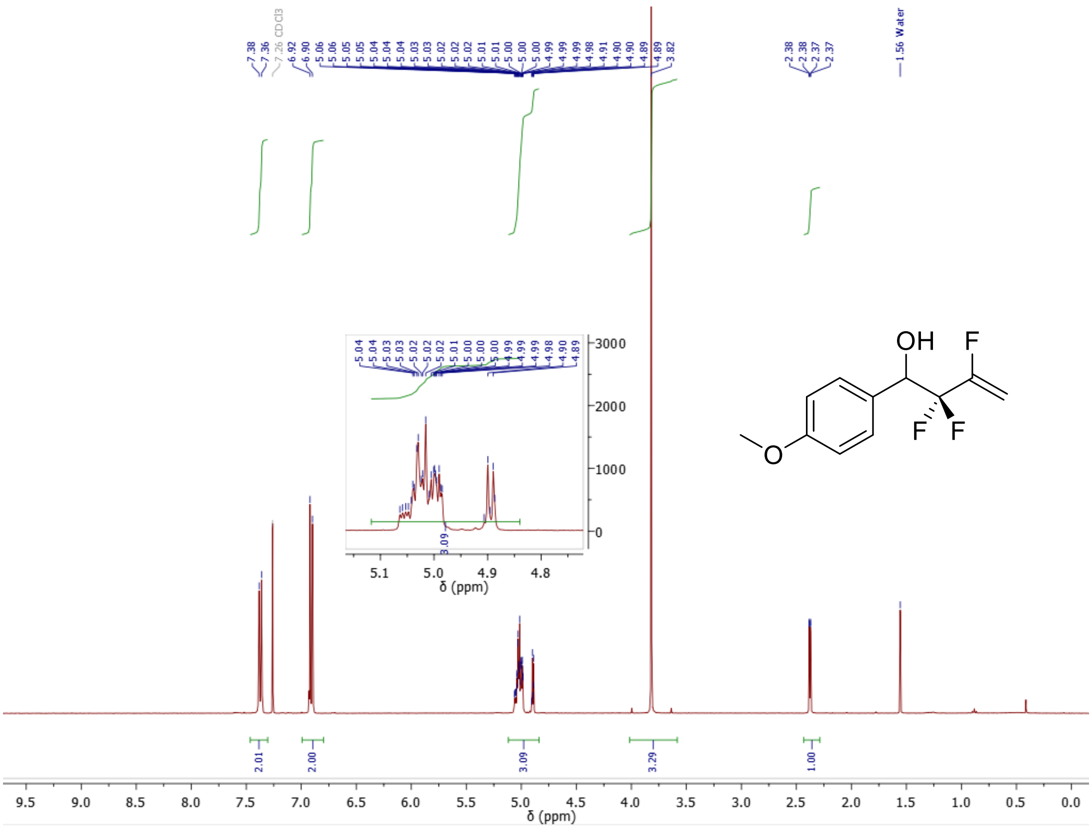

^{1}H NMR (400 MHz, CDCl₃) spectra of 2,2,3-trifluoro-1-(4-methoxyphenyl)but-3-en-1-ol, **5a**

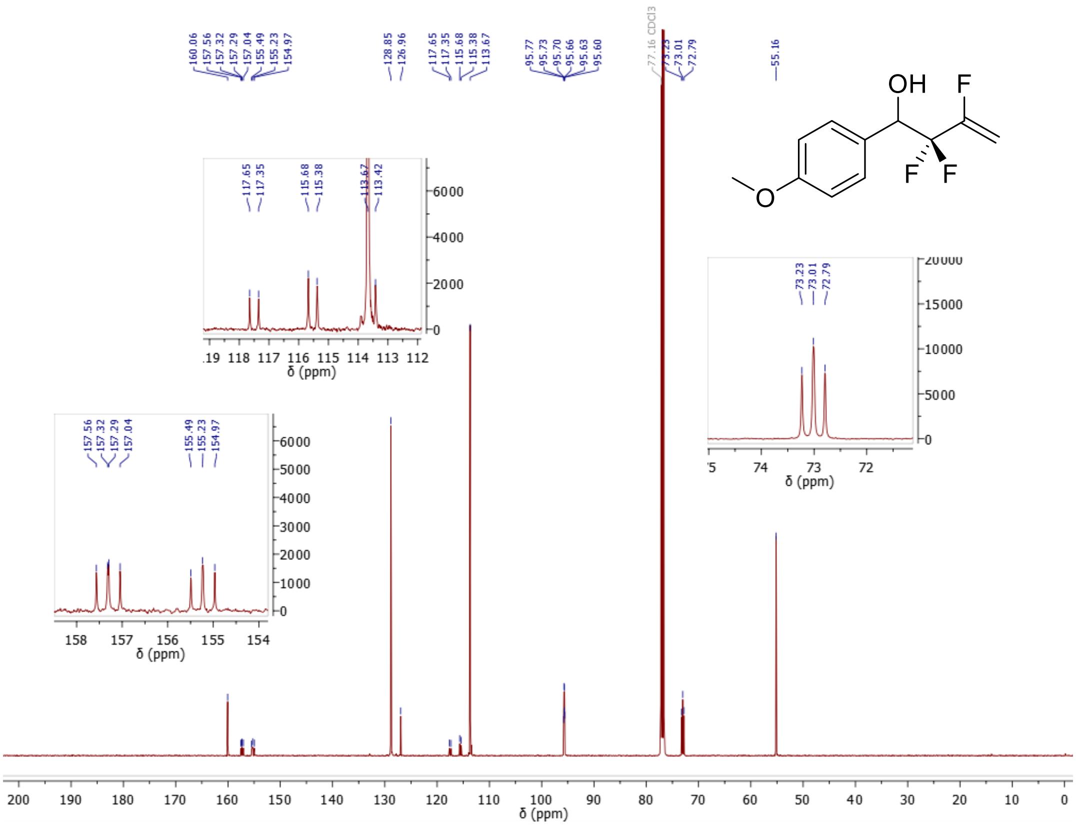

^{13}C NMR (125 MHz, CDCl₃) spectra of 2,2,3-trifluoro-1-(4-methoxyphenyl)but-3-en-1-ol, **5a**

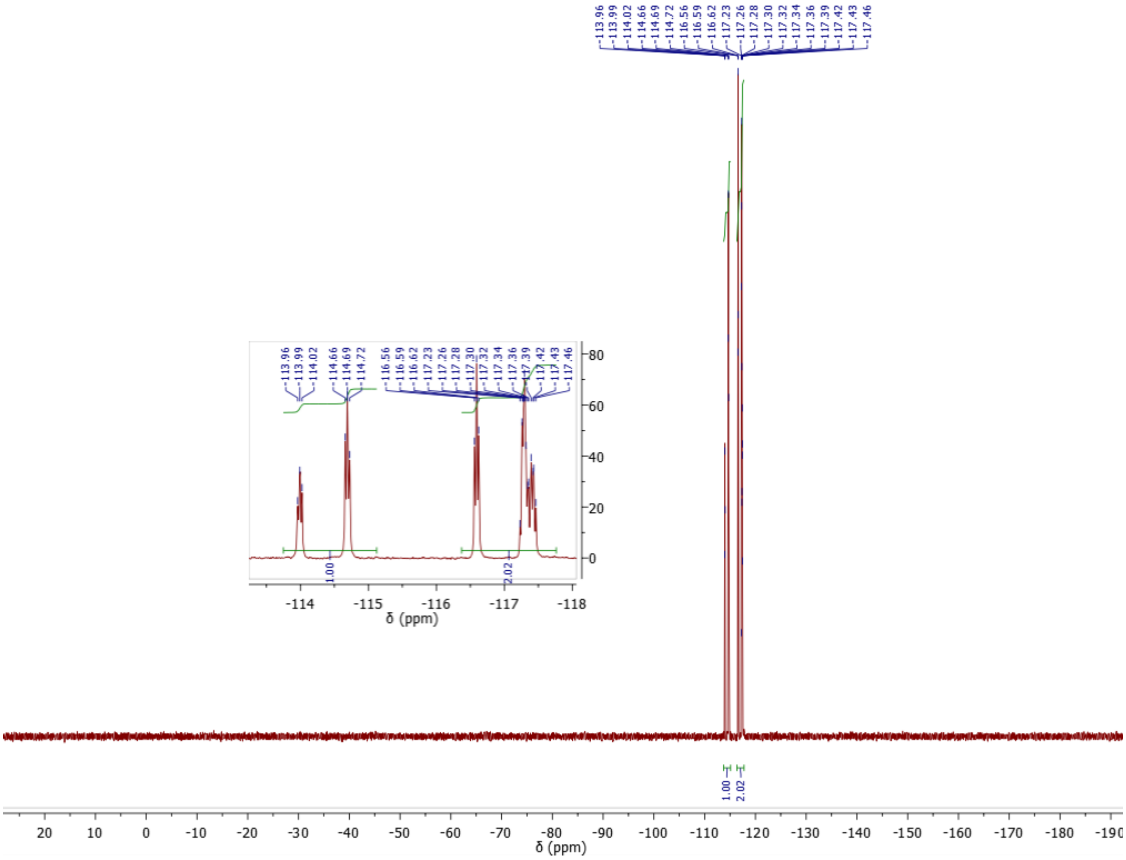

^{19}F NMR (376 MHz, $CDCl_3$) spectra of 2,2,3-trifluoro-1-(4-methoxyphenyl)but-3-en-1-ol, **5a**

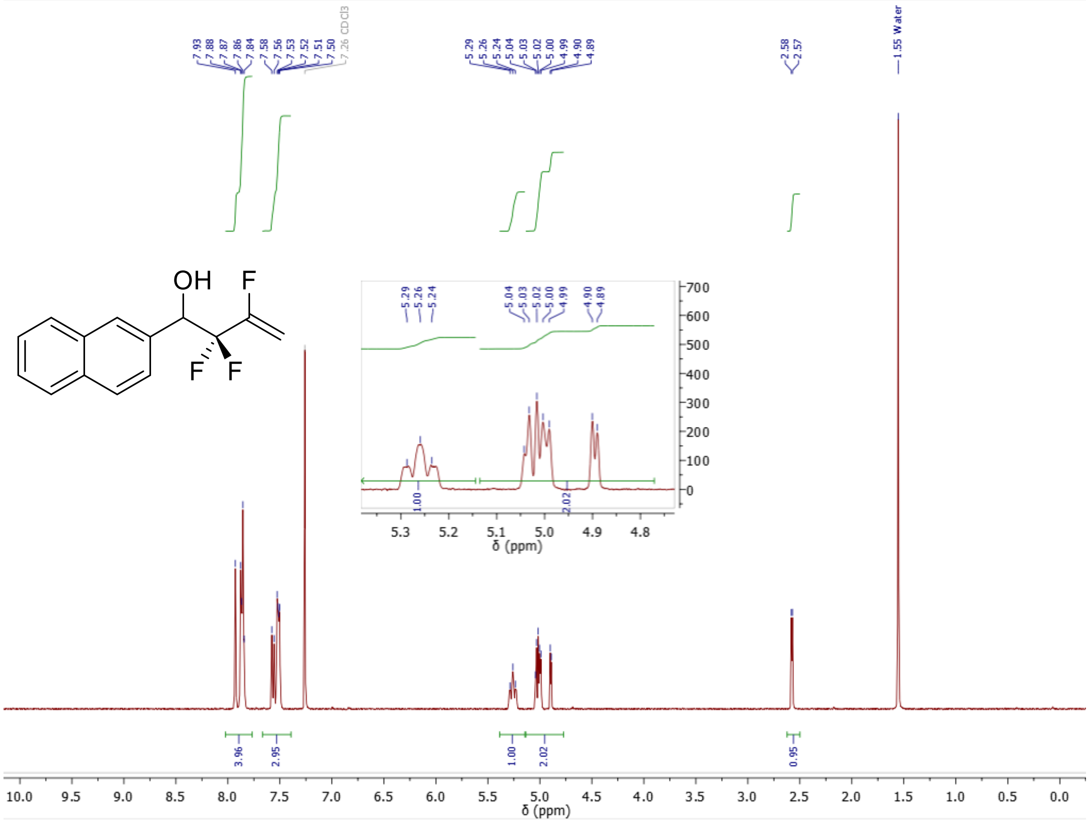

^{1}H NMR (400 MHz, $CDCl_3$) spectra of 2,2,3-trifluoro-1-(naphthalen-2-yl)but-3-en-1-ol, **5b**

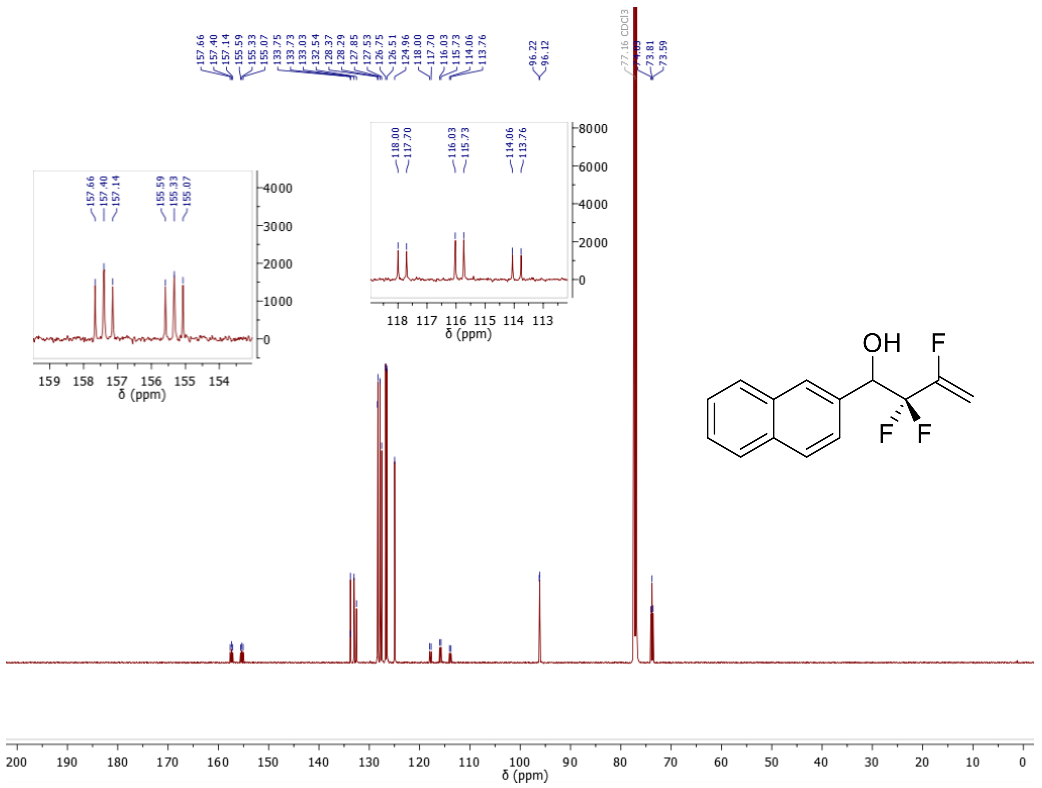

^{13}C NMR (125 MHz, $CDCl_3$) spectra of 2,2,3-trifluoro-1-(naphthalen-2-yl)but-3-en-1-ol, **5b**

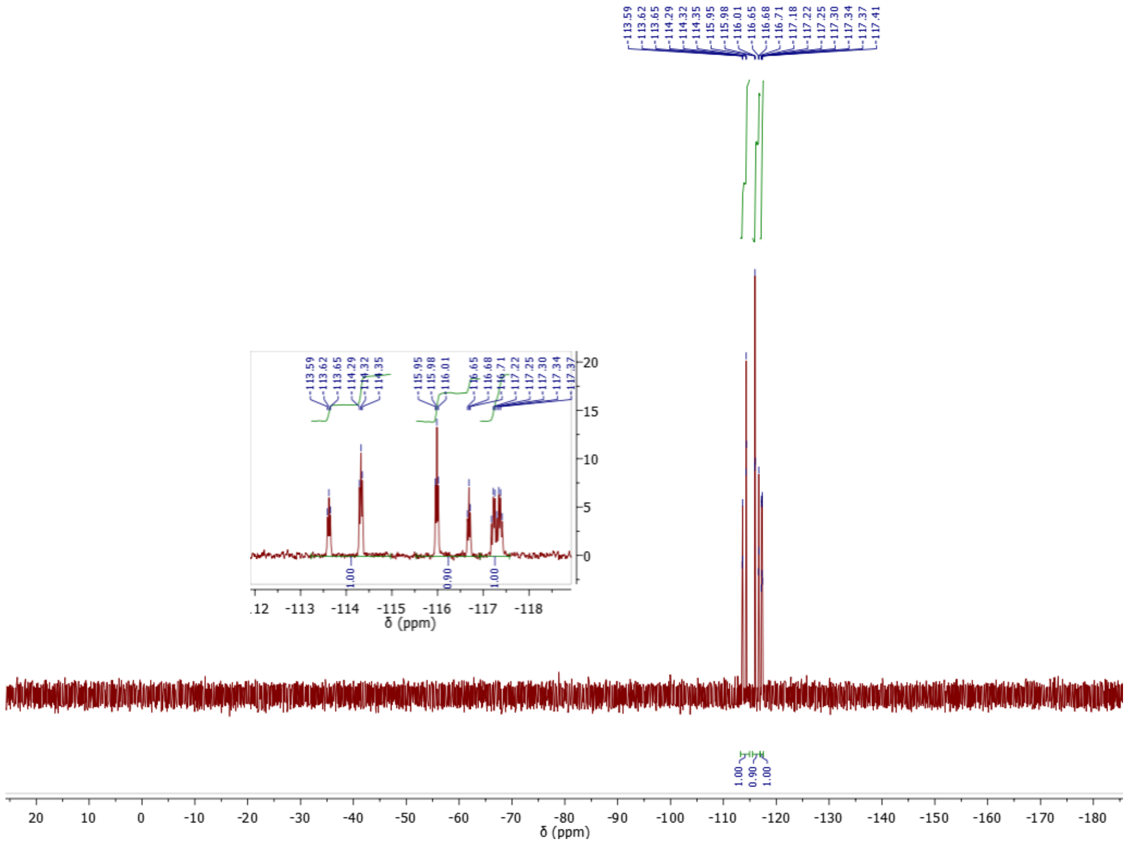

^{19}F NMR (376 MHz, $CDCl_3$) spectra of 2,2,3-trifluoro-1-(naphthalen-2-yl)but-3-en-1-ol, **5b**

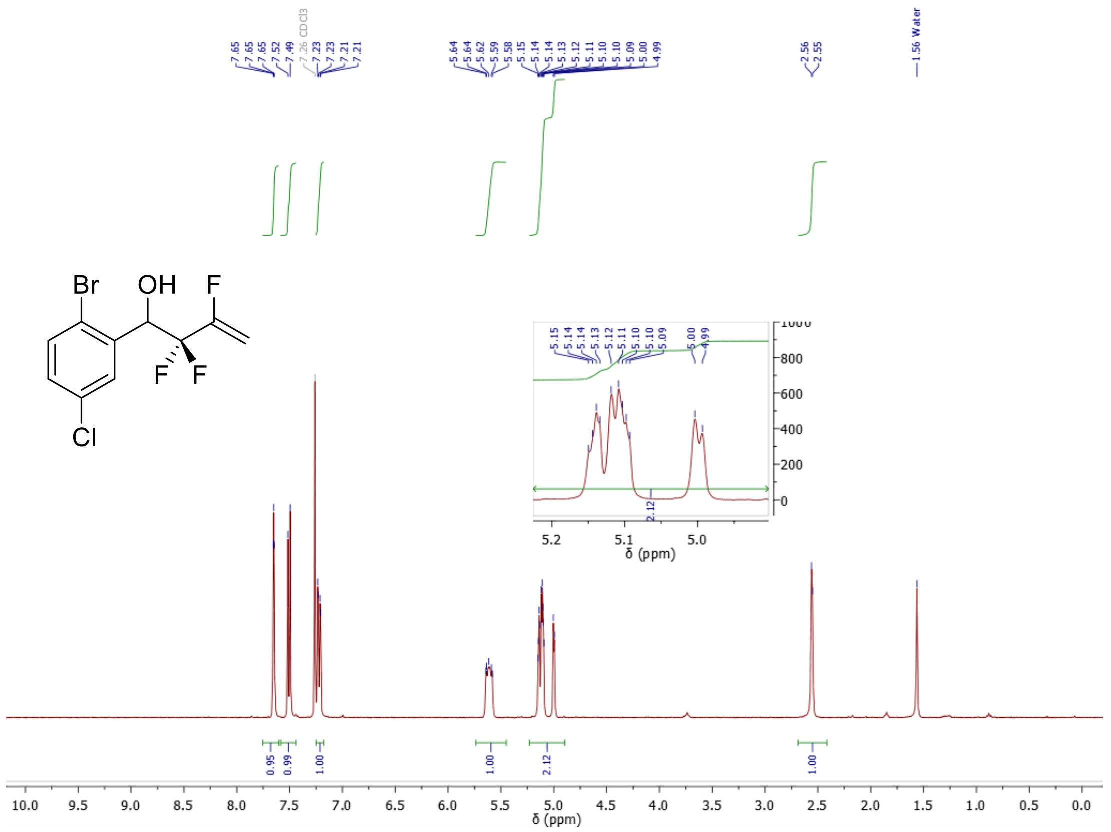

^{1}H NMR (400 MHz, $CDCl_3$) spectra of 1-(2-bromo-5-chlorophenyl)-2,2,3-trifluorobut-3-en-1-ol, **5c**

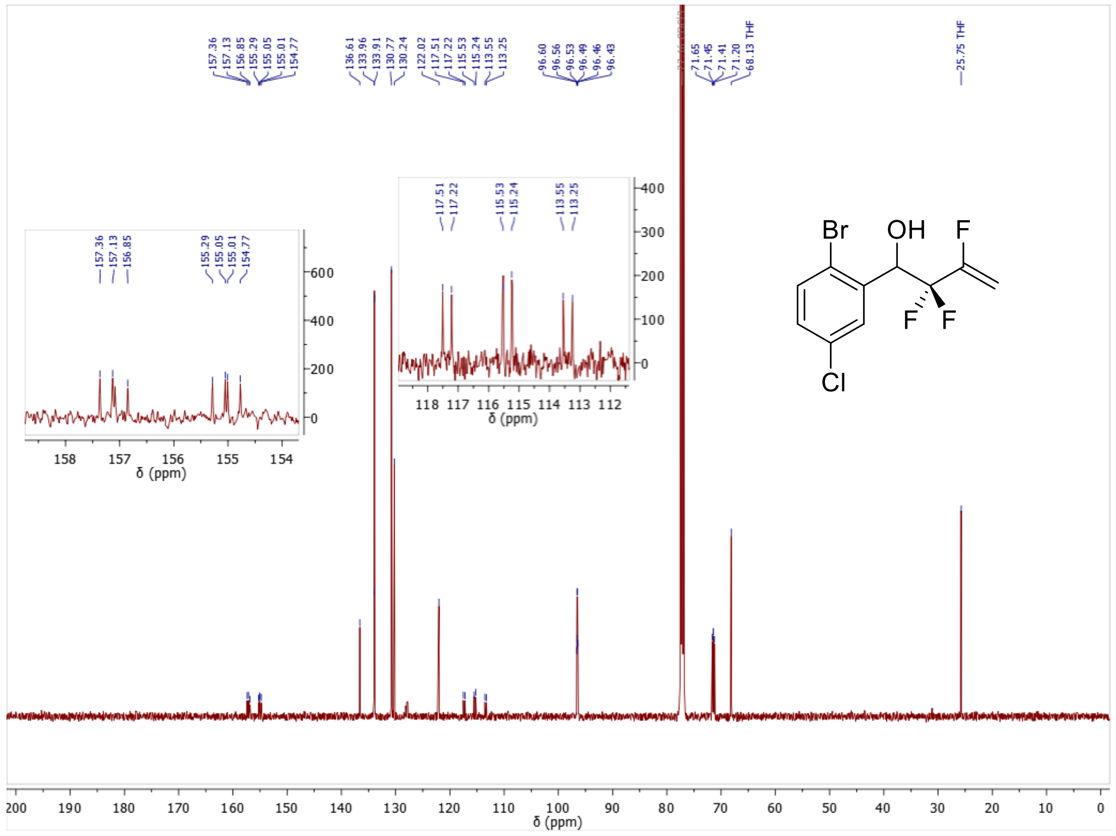

^{13}C NMR (125 MHz, $CDCl_3$) spectra of 1-(2-bromo-5-chlorophenyl)-2,2,3-trifluorobut-3-en-1-ol, **5c**

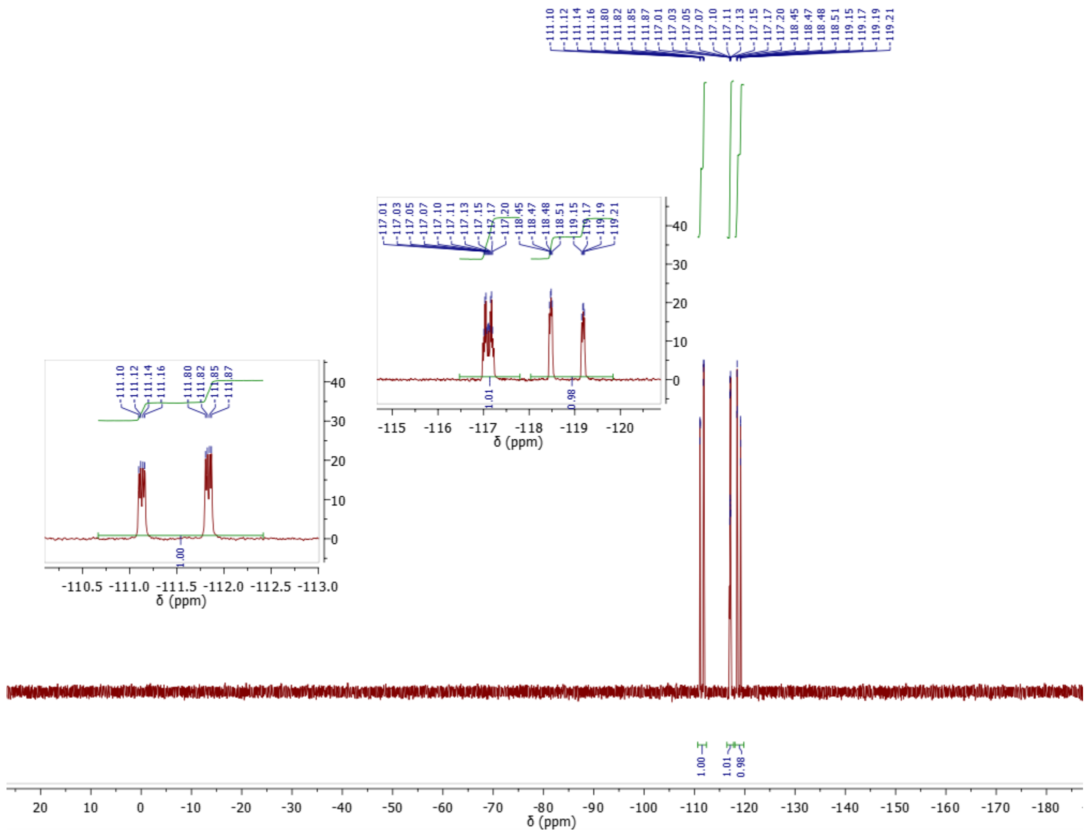

^{19}F NMR (376 MHz, $CDCl_3$) spectra of 1-(2-bromo-5-chlorophenyl)-2,2,3-trifluorobut-3-en-1-ol, **5c**

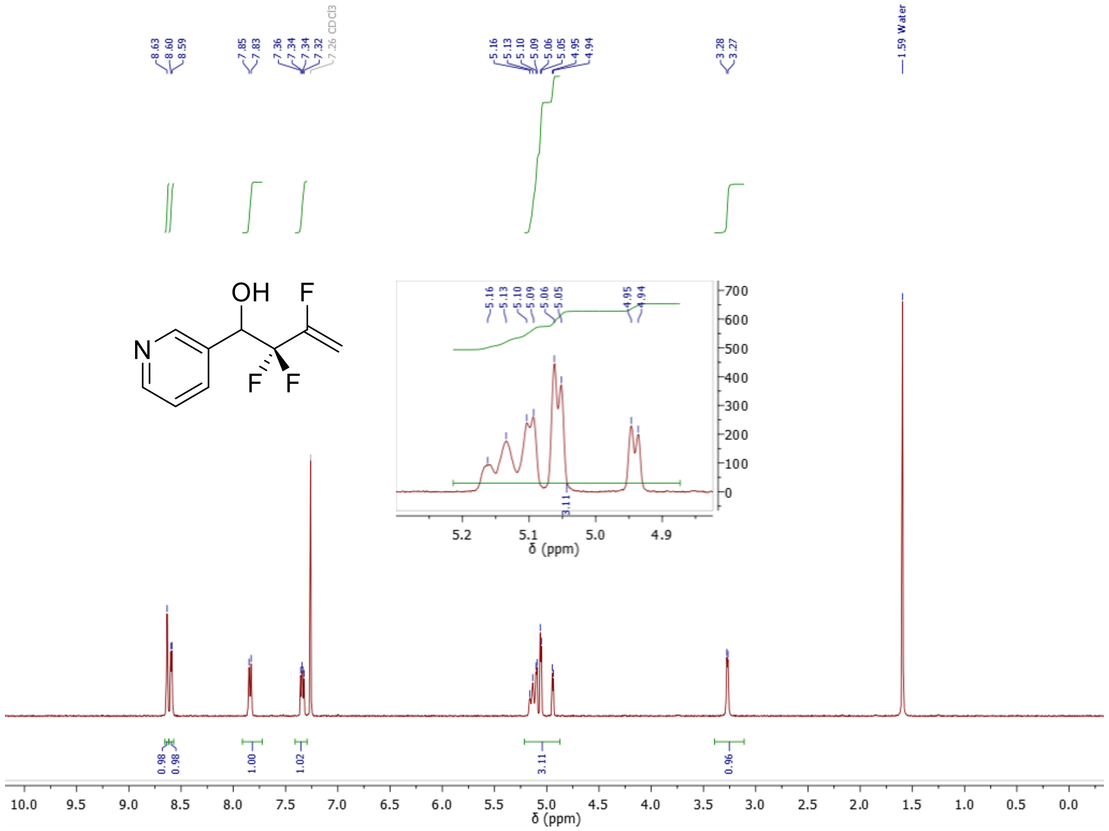

^{1}H NMR (400 MHz, $CDCl_3$) spectra of 2,2,3-trifluoro-1-(pyridin-3-yl)but-3-en-1-ol, **5d**

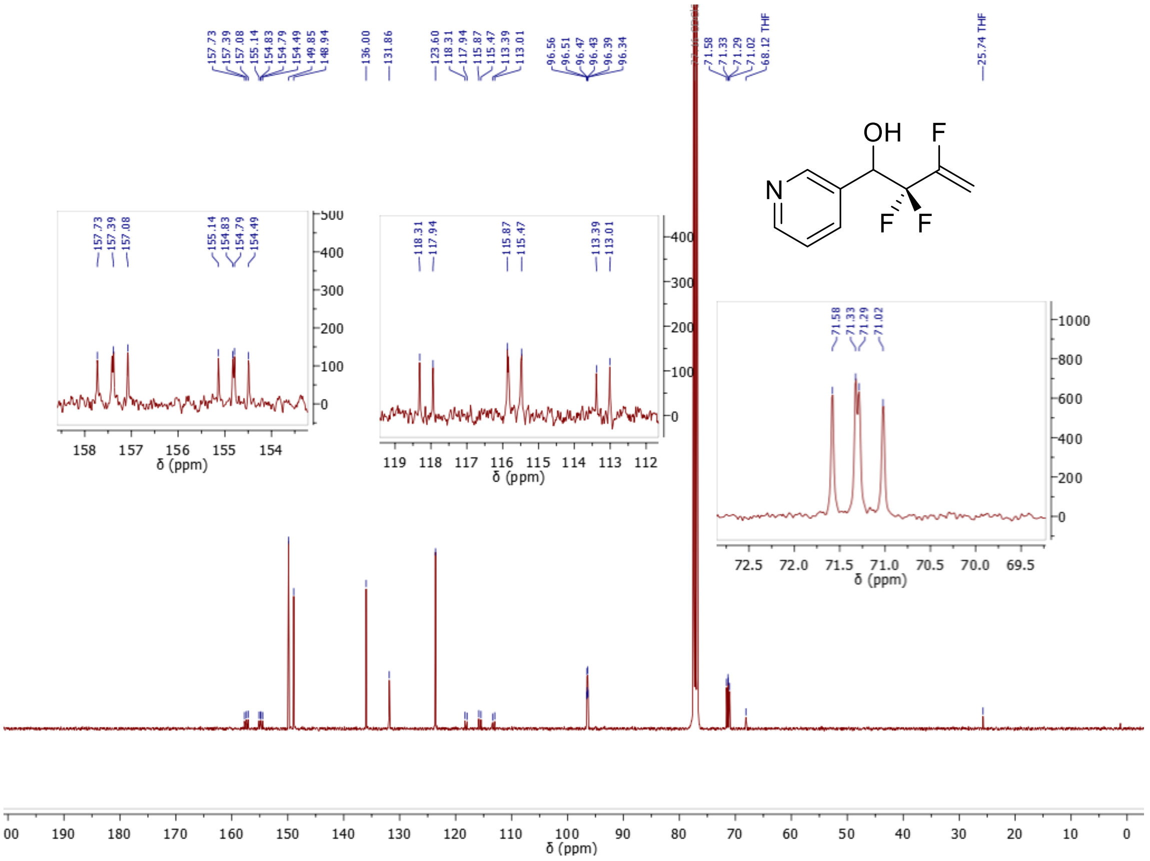

^{13}C NMR (100 MHz, $CDCl_3$) spectra of 2,2,3-trifluoro-1-(pyridin-3-yl)but-3-en-1-ol, **5d**

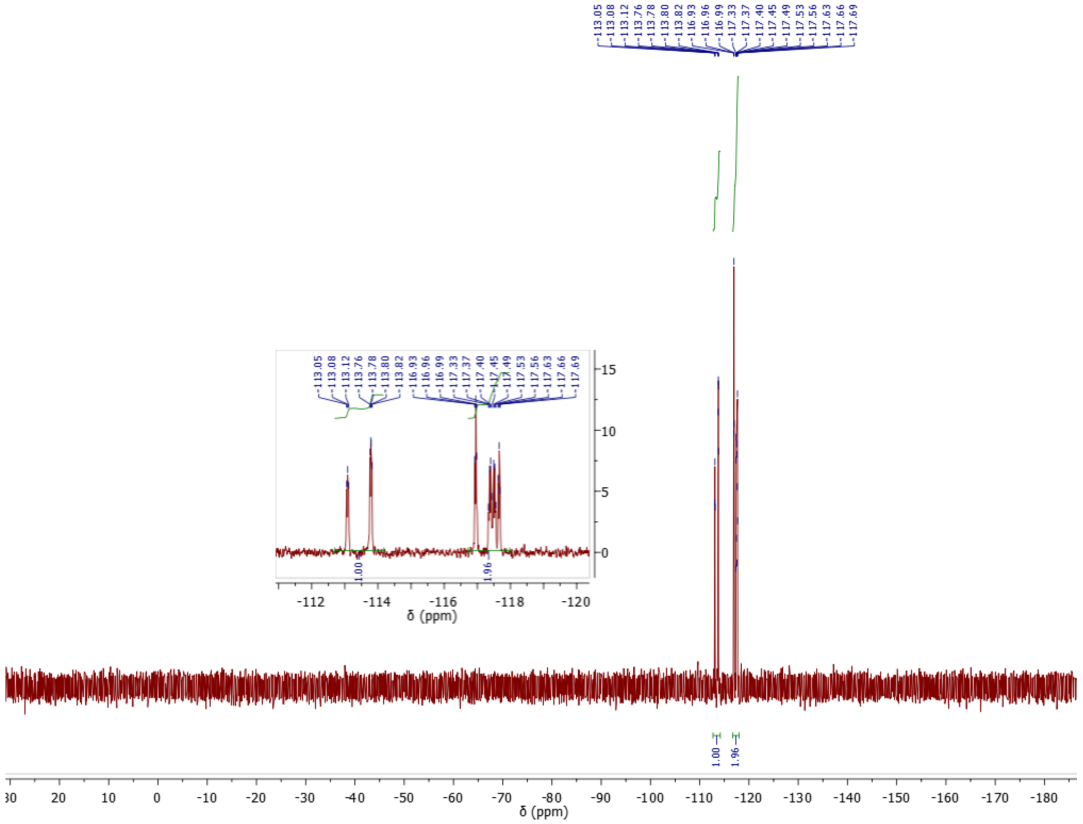

^{19}F NMR (376 MHz, $CDCl_3$) spectra of 2,2,3-trifluoro-1-(pyridin-3-yl)but-3-en-1-ol, **5d**

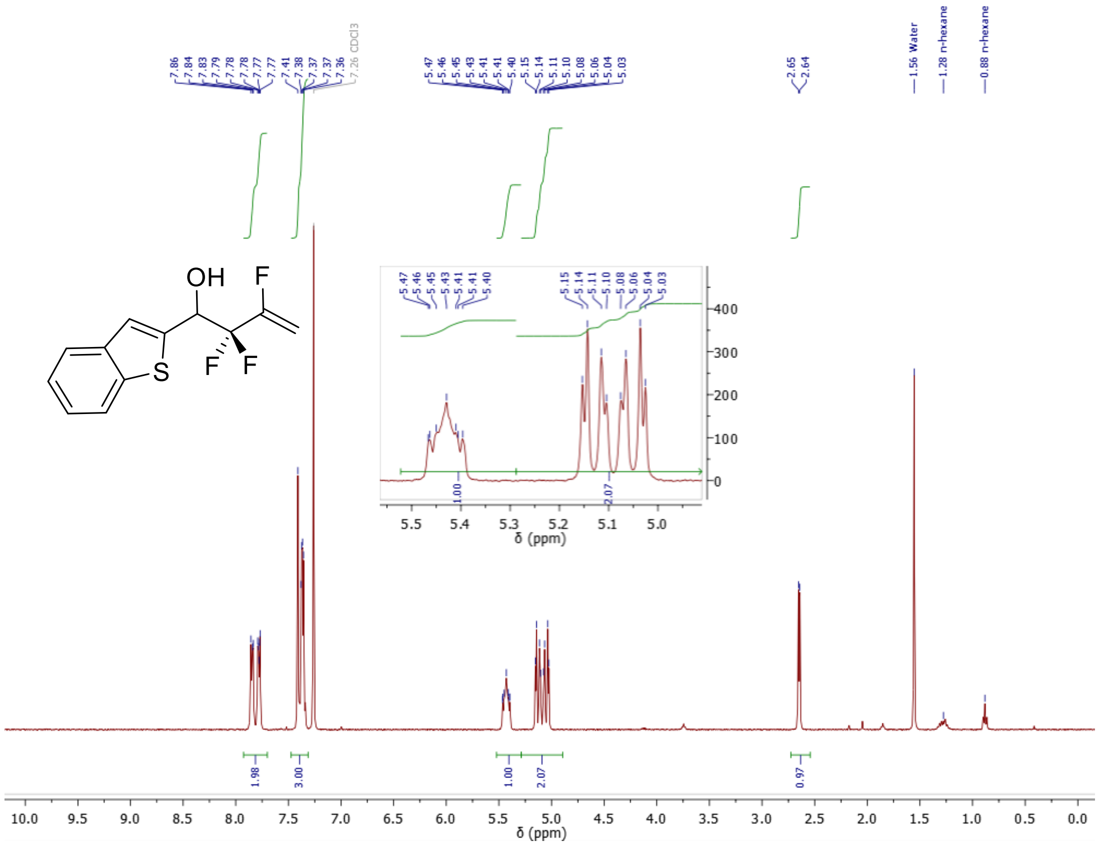

^{1}H NMR (400 MHz, $CDCl_3$) spectra of 1-(benzo[b]thiophen-2-yl)-2,2,3-trifluorobut-3-en-1-ol, **5e**

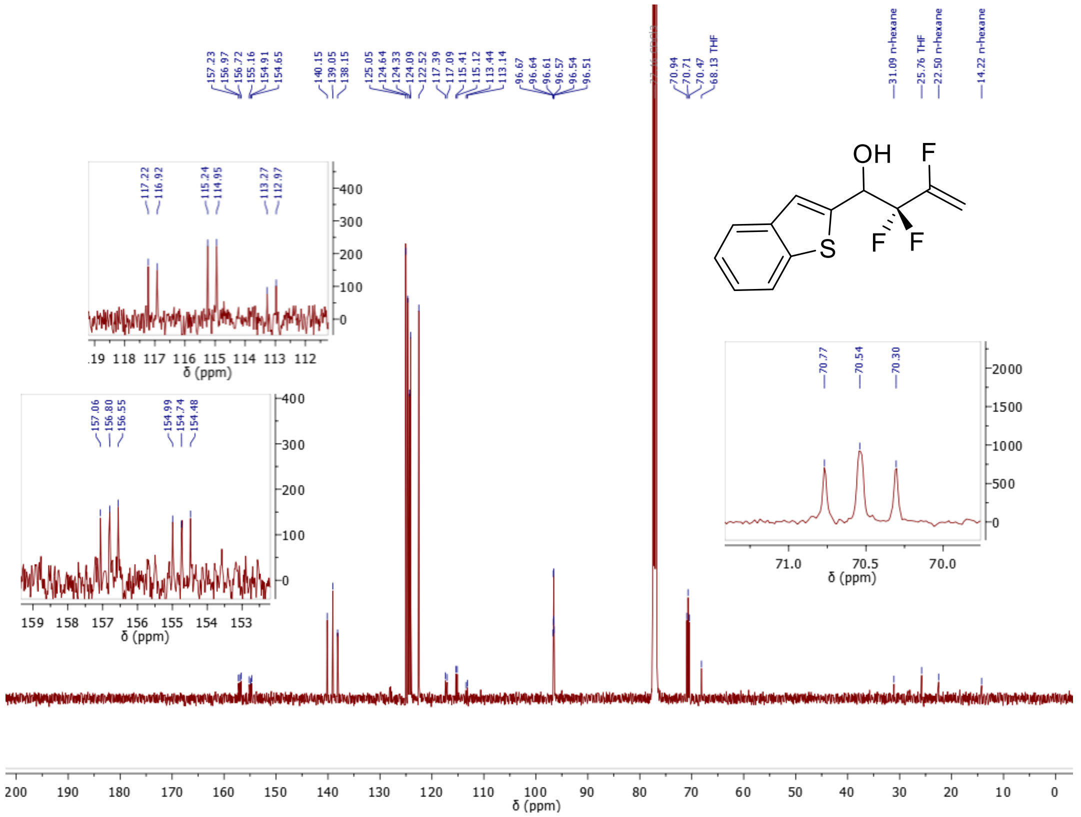

^{13}C NMR (125 MHz, $CDCl_3$) spectra of 1-(benzo[b]thiophen-2-yl)-2,2,3-trifluorobut-3-en-1-ol, **5e**

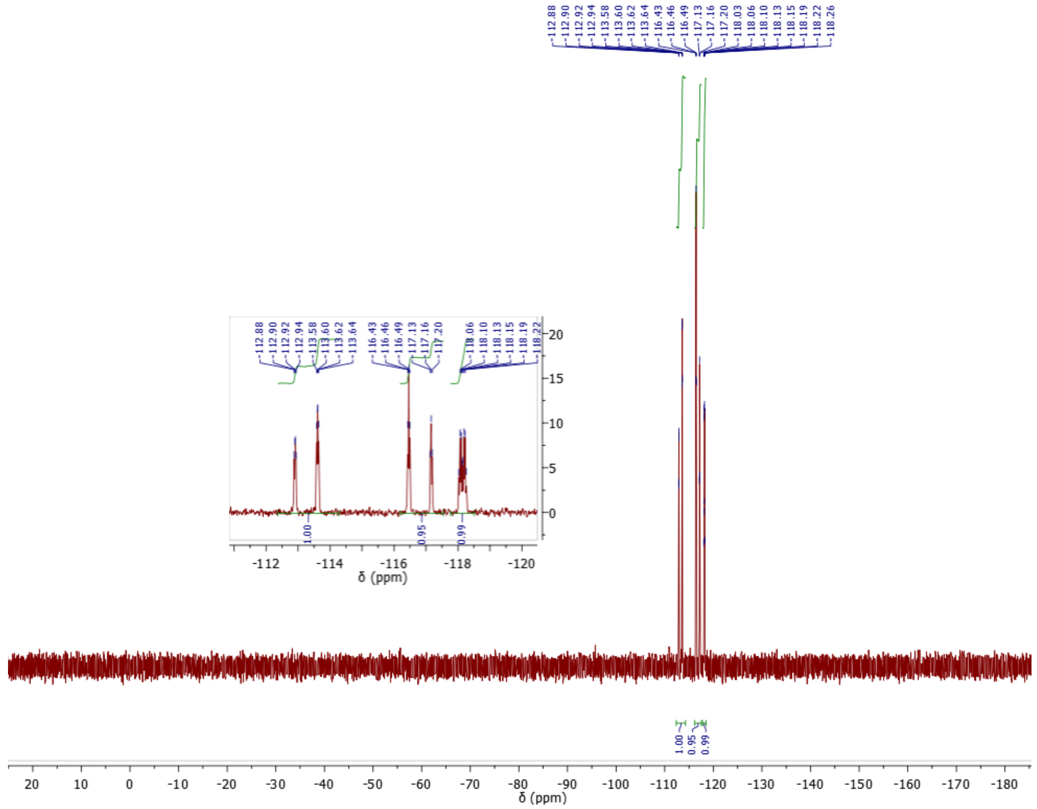

^{19}F NMR (376 MHz, $CDCl_3$) spectra of 1-(benzo[b]thiophen-2-yl)-2,2,3-trifluorobut-3-en-1-ol, **5e**

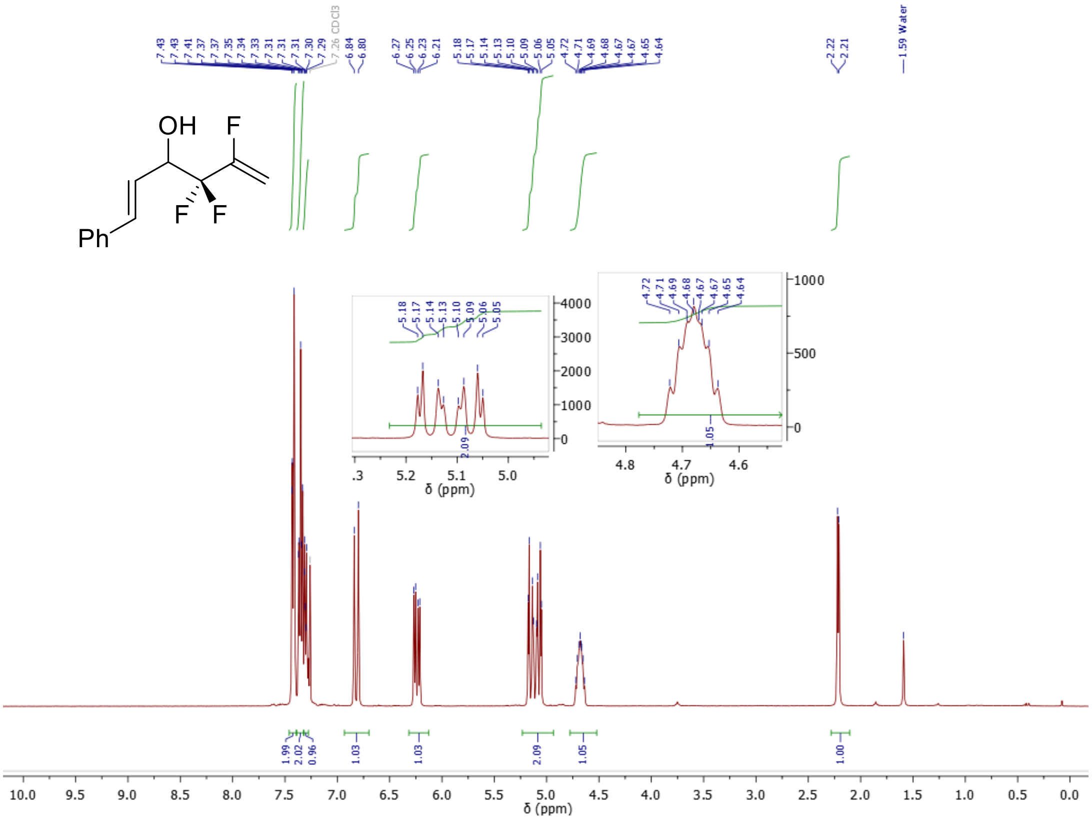

^{1}H NMR (400 MHz, $CDCl_3$) spectra of (E)-4,4,5-trifluoro-1-phenylhexa-1,5-dien-3-ol, **5f**

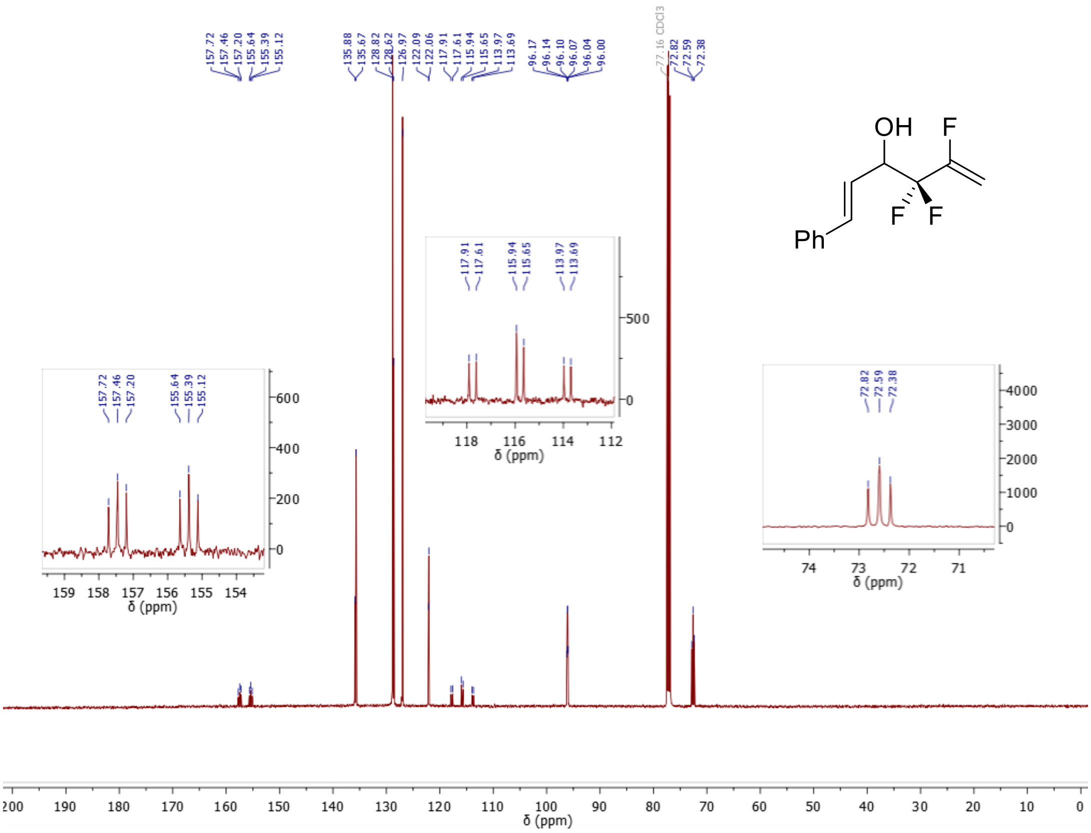

^{13}C NMR (125 MHz, $CDCl_3$) spectra of (E)-4,4,5-trifluoro-1-phenylhexa-1,5-dien-3-ol, **5f**

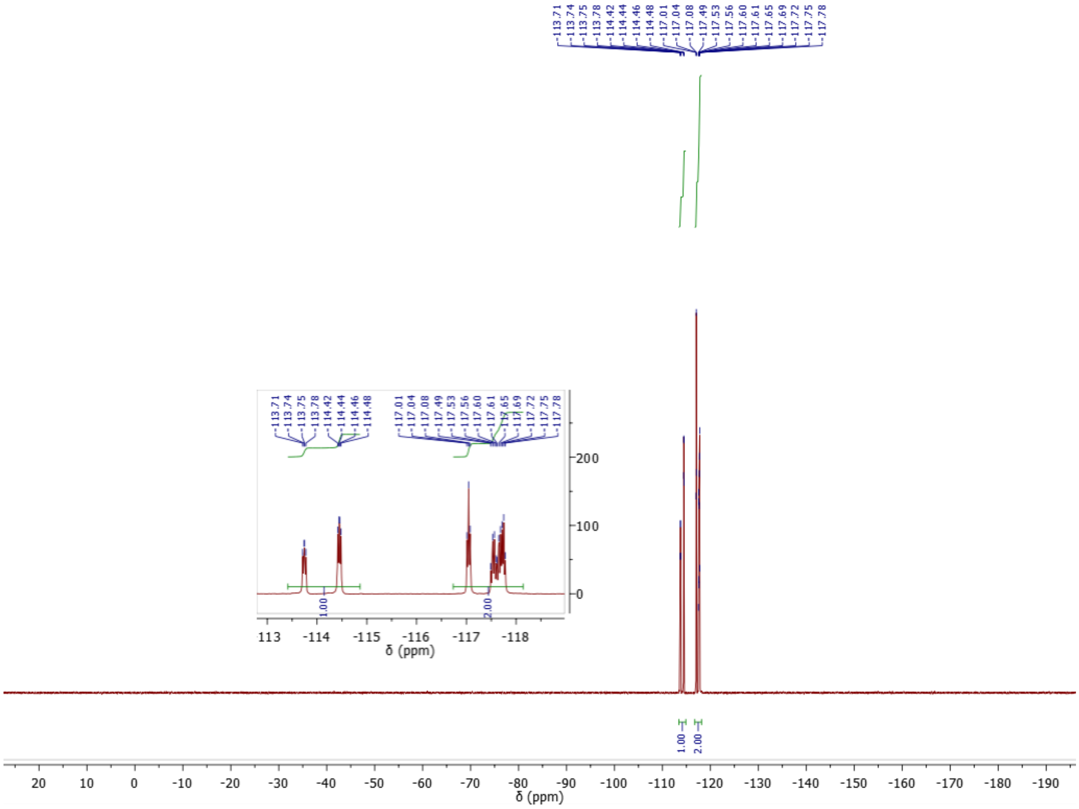

^{19}F NMR (376 MHz, $CDCl_3$) spectra of (E)-4,4,5-trifluoro-1-phenylhexa-1,5-dien-3-ol, **5f**

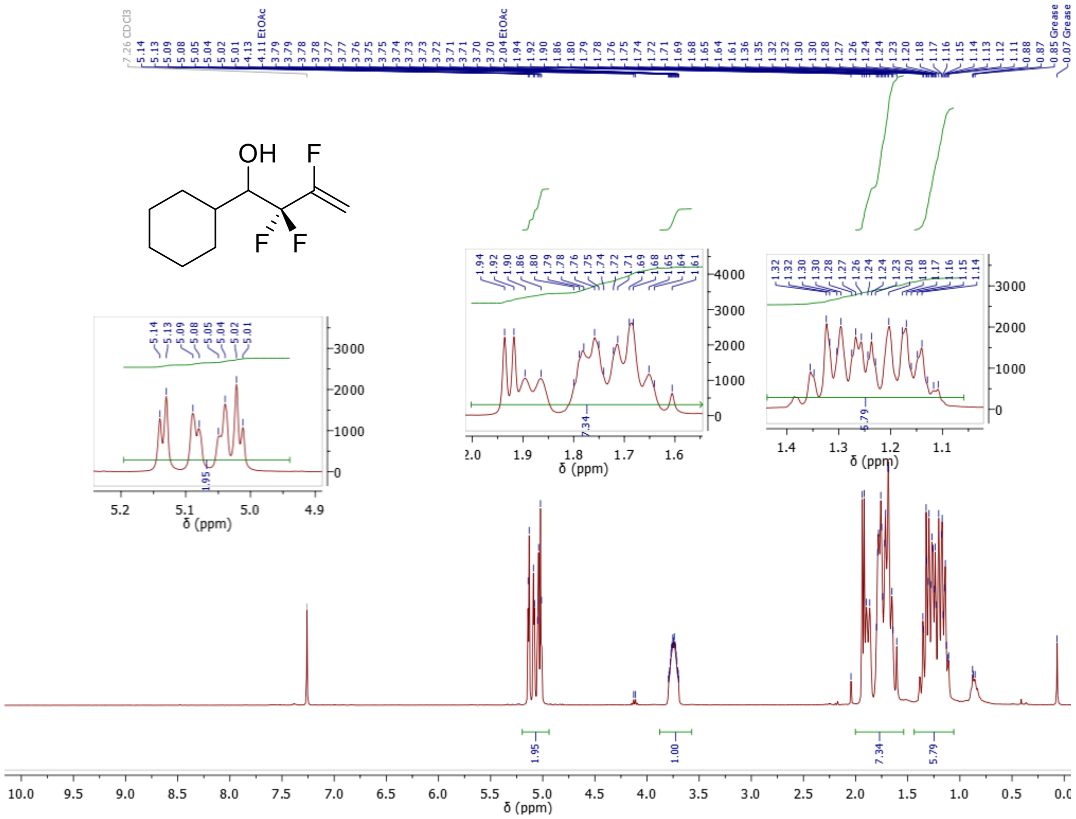

^{1}H NMR (400 MHz, $CDCl_3$) spectra of 1-cyclohexyl-2,2,3-trifluorobut-3-en-1-ol, **5g**

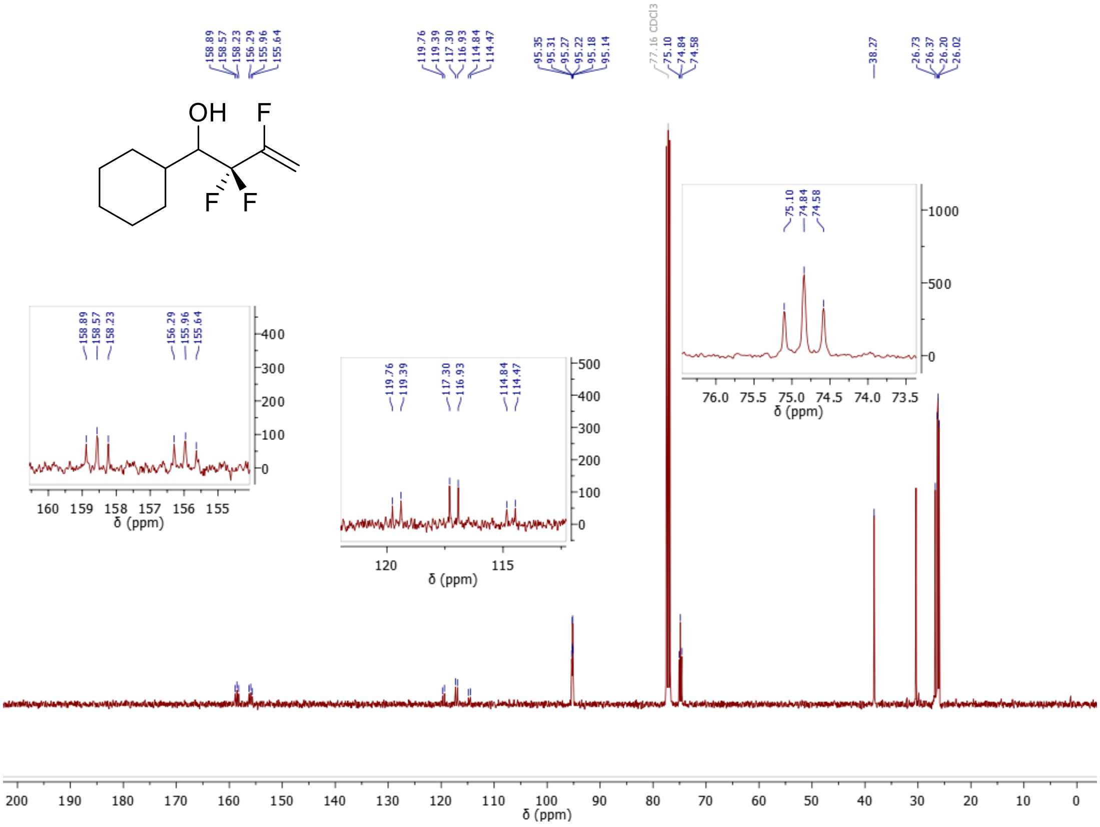

^{13}C NMR (100 MHz, $CDCl_3$) spectra of 1-cyclohexyl-2,2,3-trifluorobut-3-en-1-ol, **5g**

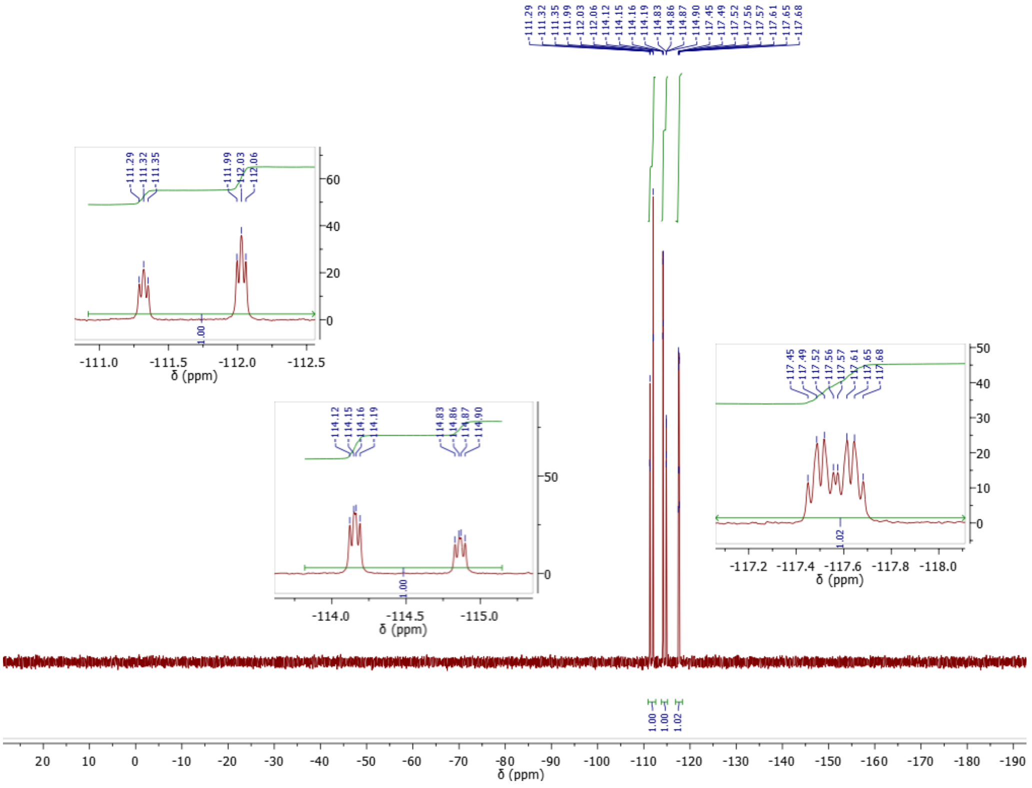

^{19}F NMR (376 MHz, $CDCl_3$) spectra of 1-cyclohexyl-2,2,3-trifluorobut-3-en-1-ol, **5g**

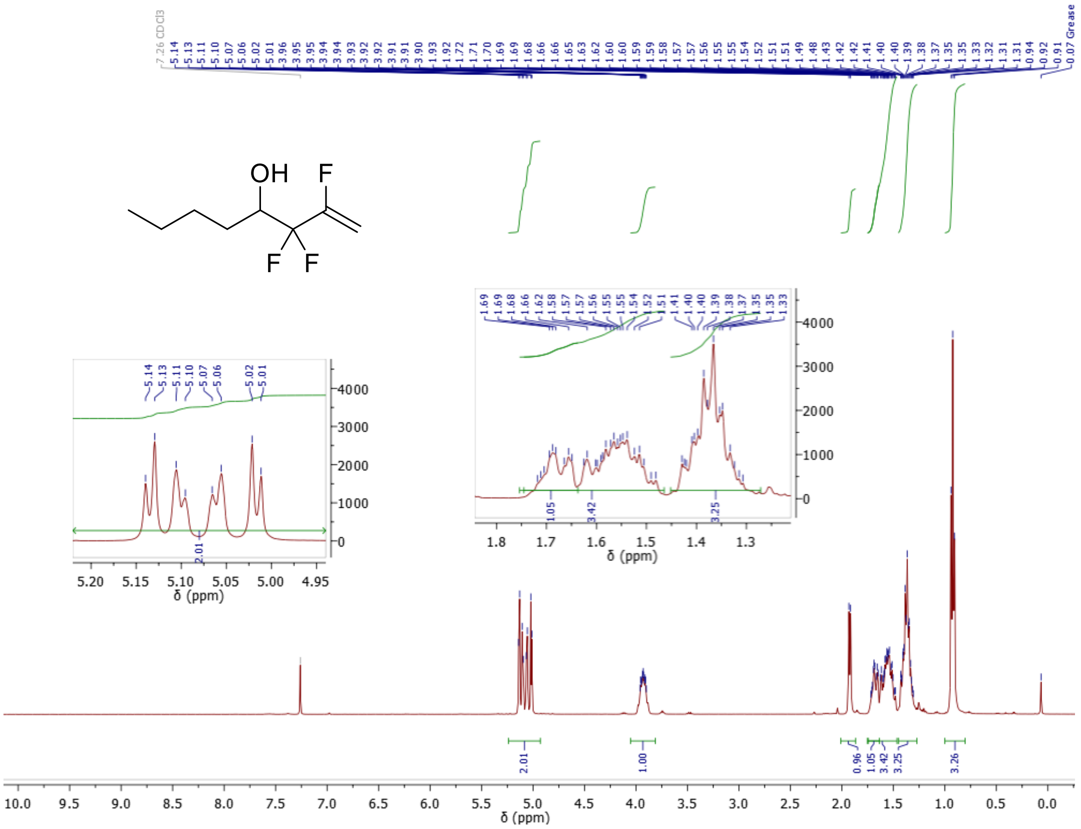

^{1}H NMR (400 MHz, $CDCl_3$) spectra of 2,3,3-trifluorooct-1-en-4-ol, **5h**

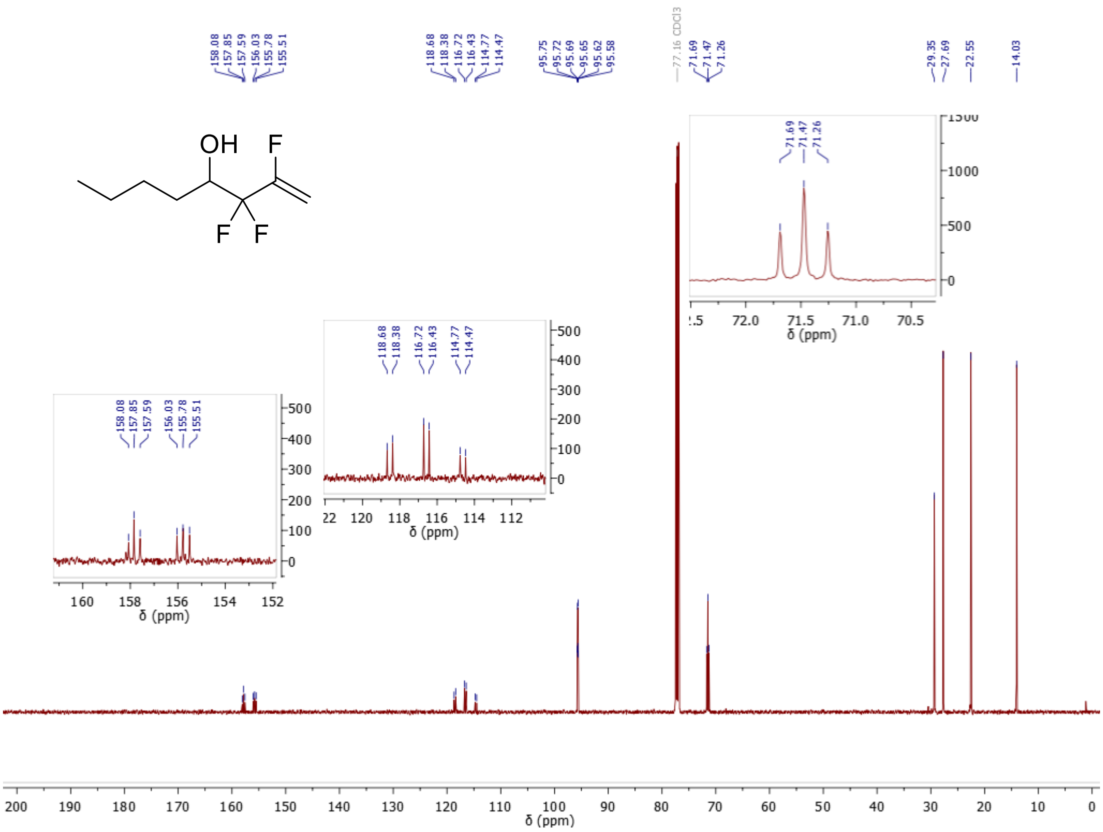

^{13}C NMR (125 MHz, $CDCl_3$) spectra of 2,3,3-trifluorooct-1-en-4-ol, **5h**

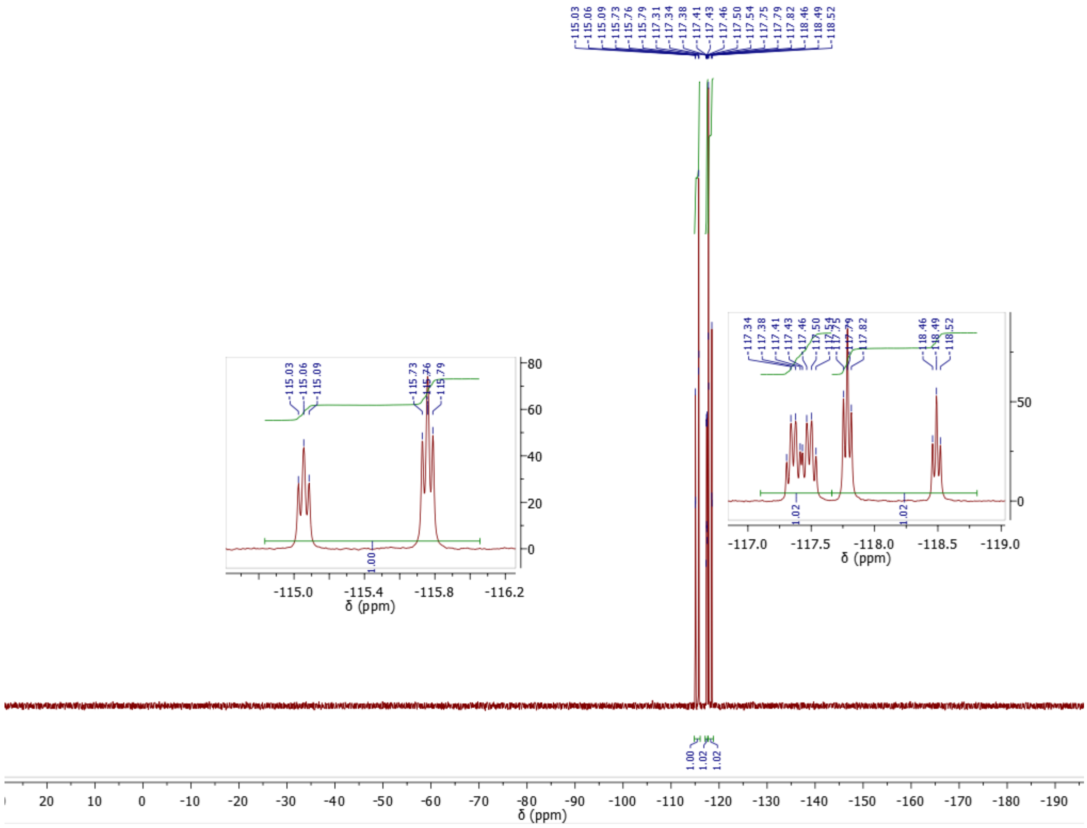

^{19}F NMR (376 MHz, $CDCl_3$) spectra of 2,3,3-trifluorooct-1-en-4-ol, **5h**

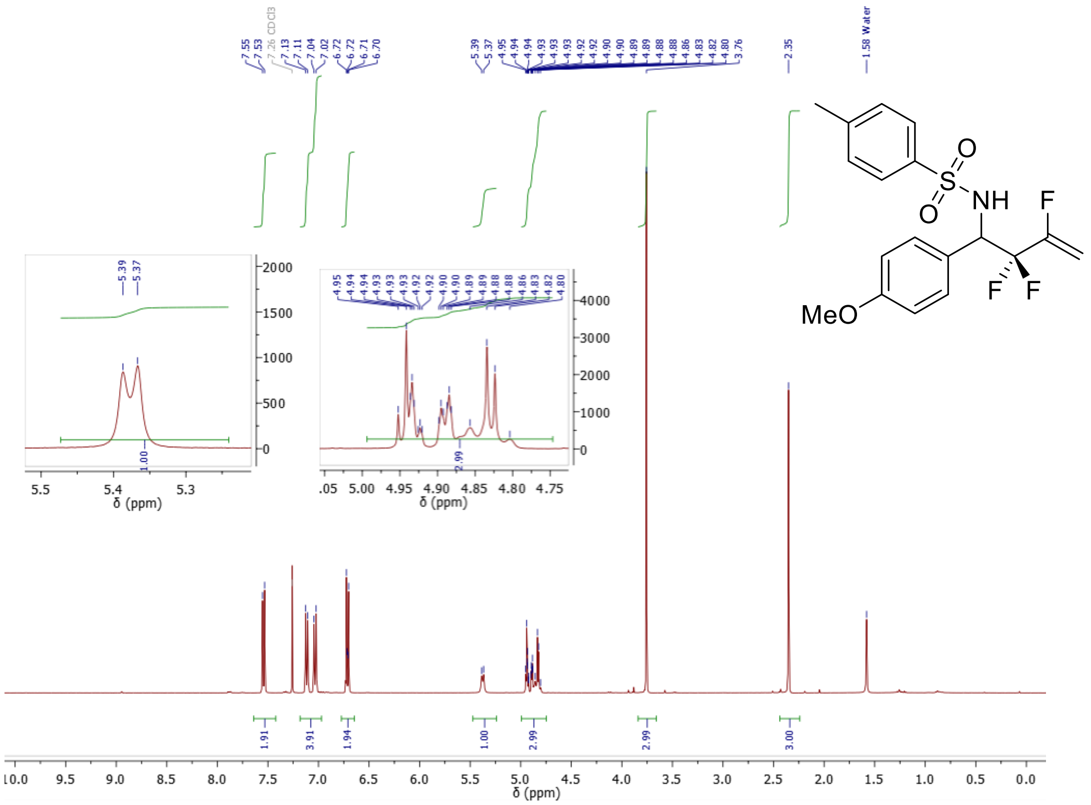

^{1}H NMR (400 MHz, $CDCl_3$) spectra of 4-methyl-N-(2,2,3-trifluoro-1-(4-methoxyphenyl)but-3-en-1-yl)benzenesulfonamide, **5i**

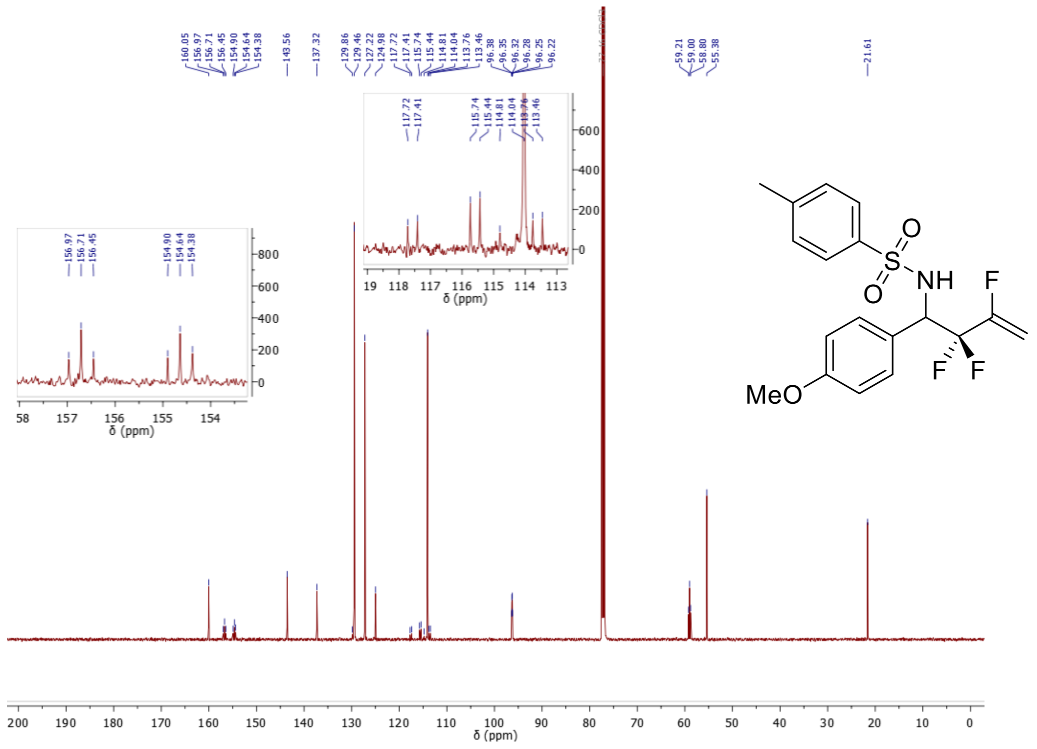

^{13}C NMR (125 MHz, $CDCl_3$) spectra of 4-methyl-N-(2,2,3-trifluoro-1-(4-methoxyphenyl)but-3-en-1-yl)benzenesulfonamide, **5i**

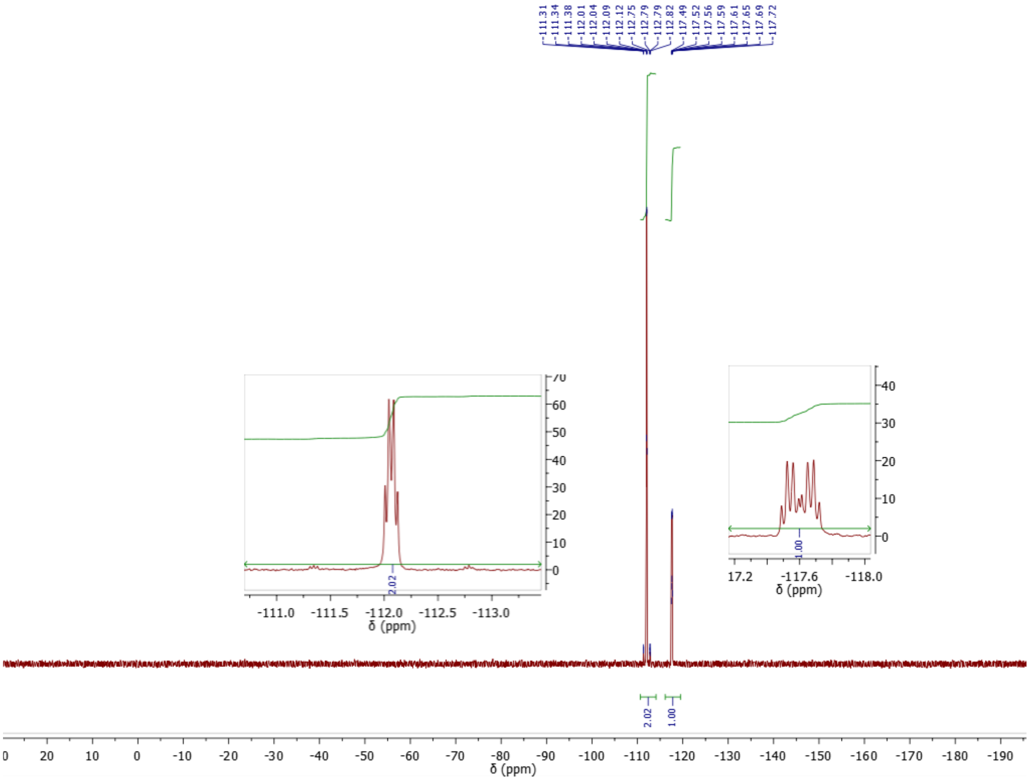

^{19}F NMR (376 MHz, $CDCl_3$) spectra of 4-methyl-N-(2,2,3-trifluoro-1-(4-methoxyphenyl)but-3-en-1-yl)benzenesulfonamide, **5i**

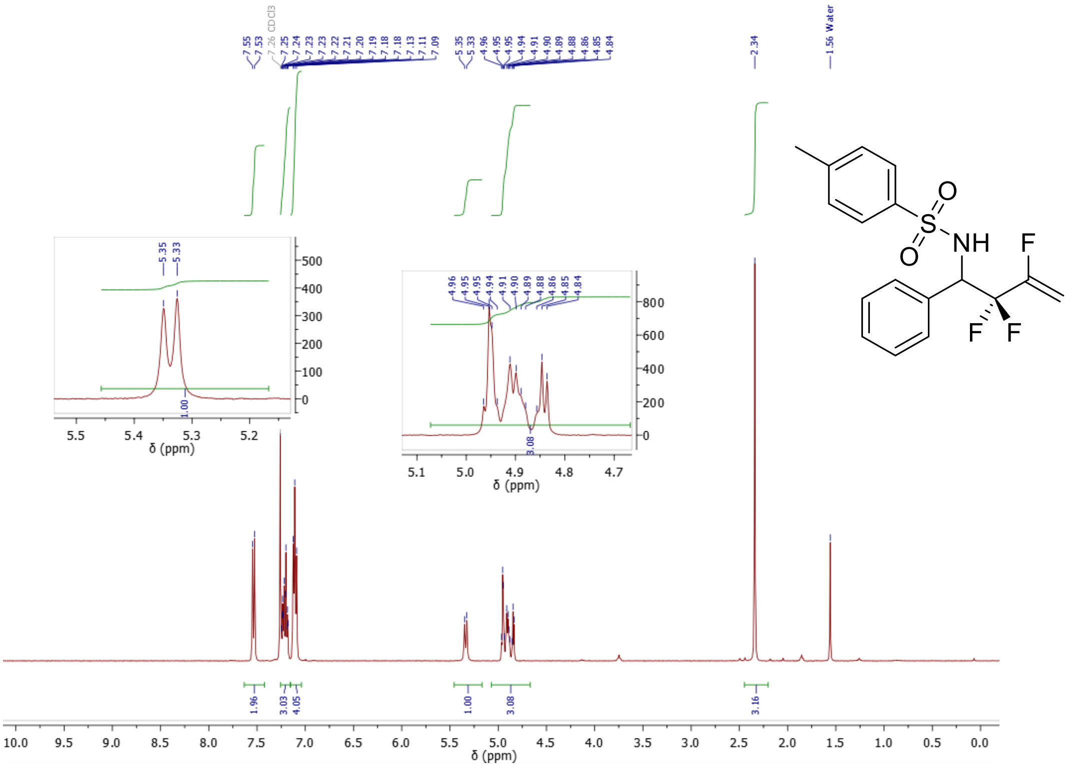

^{1}H NMR (400 MHz, $CDCl_3$) spectra of 4-methyl-N-(2,2,3-trifluoro-1-phenylbut-3-en-1-yl)benzenesulfonamide, **5j**

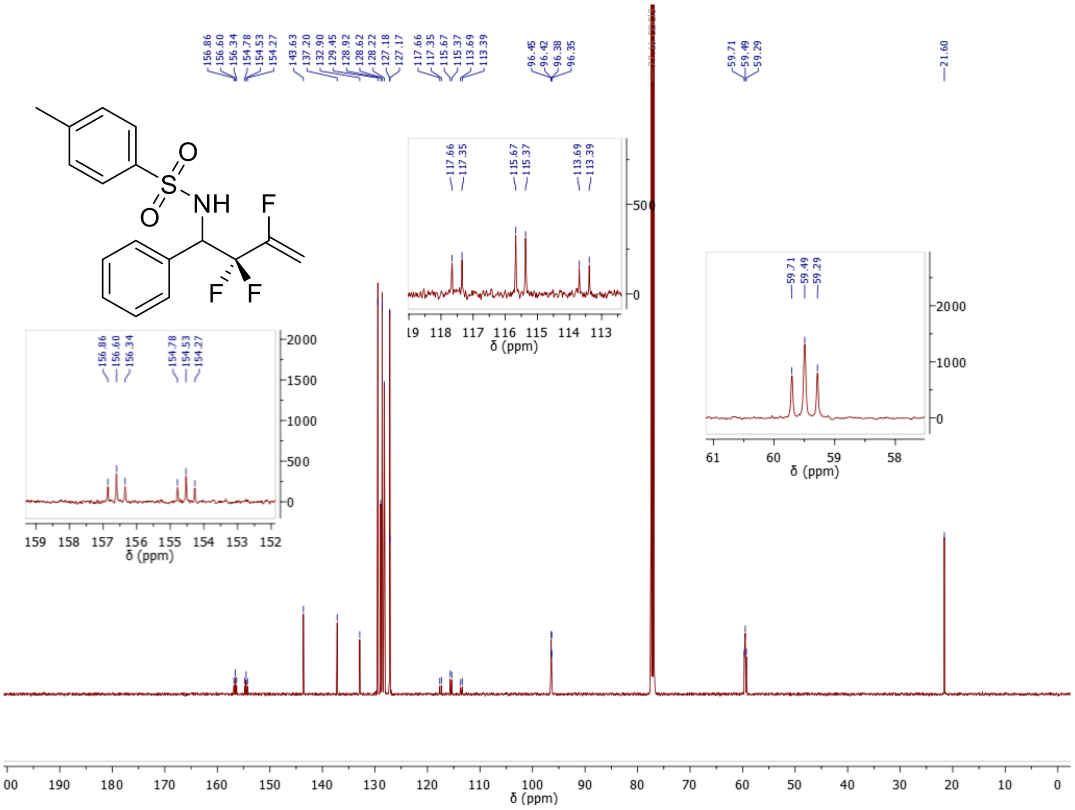

^{13}C NMR (125 MHz, $CDCl_3$) spectra of 4-methyl-N-(2,2,3-trifluoro-1-phenylbut-3-en-1-yl)benzenesulfonamide, **5j**

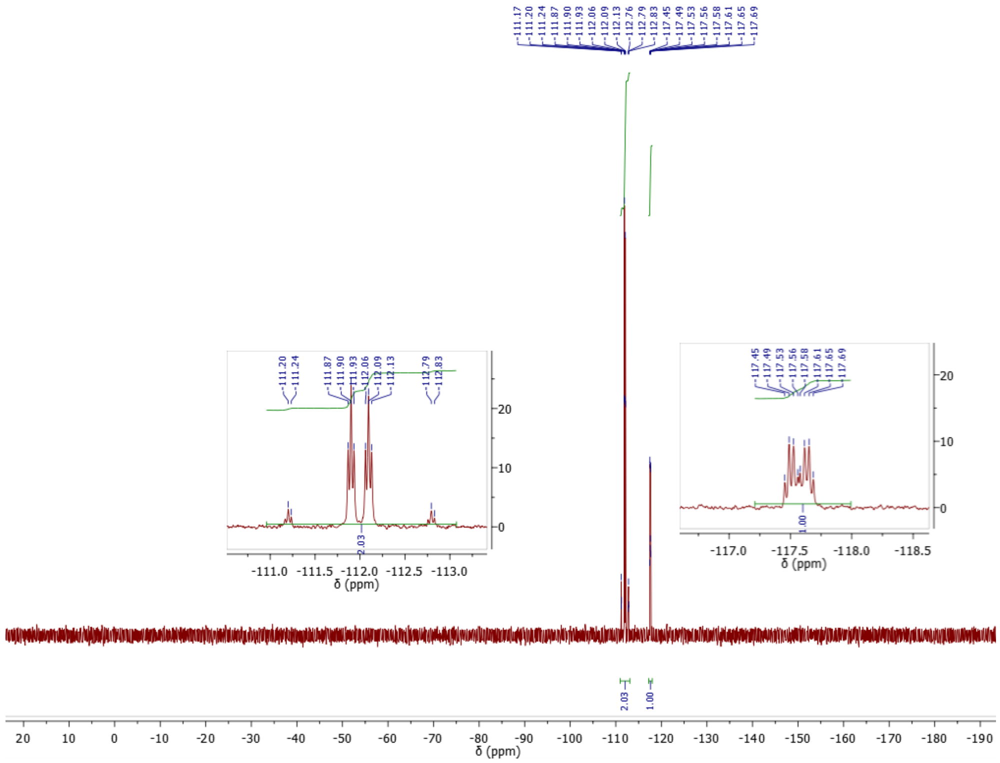

^{19}F NMR (376 MHz, $CDCl_3$) spectra of 4-methyl-N-(2,2,3-trifluoro-1-phenylbut-3-en-1-yl)benzenesulfonamide, **5j**

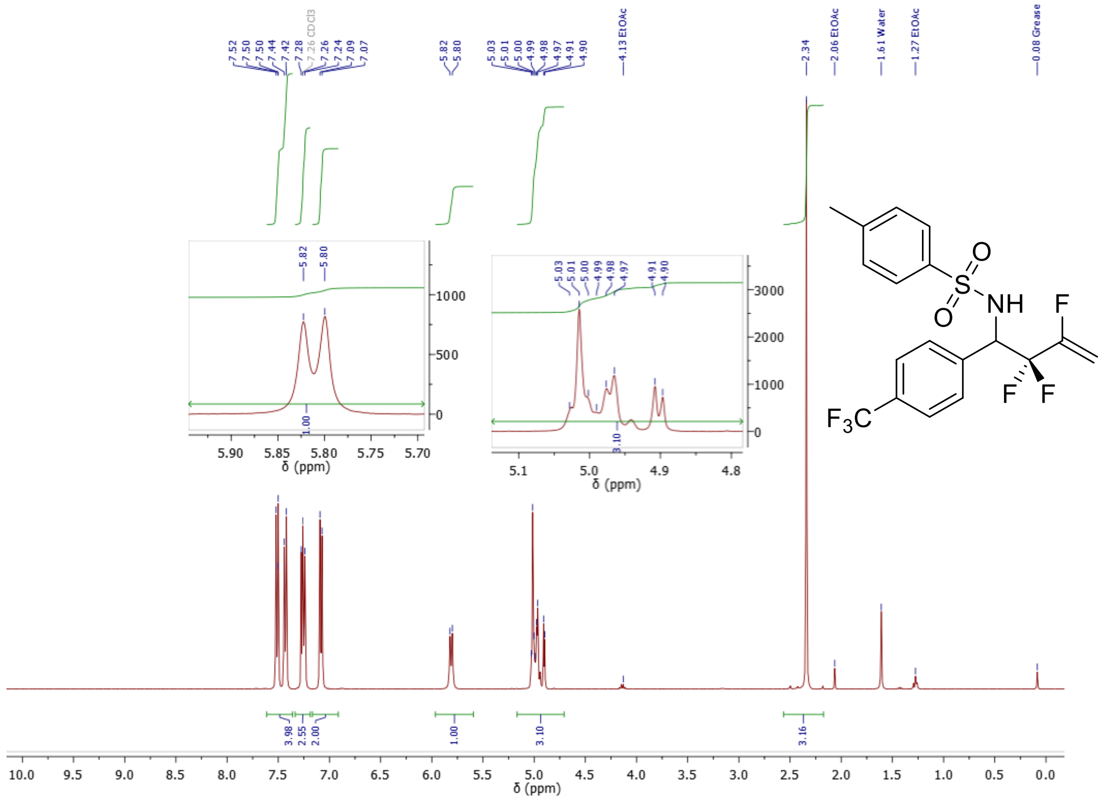

^{1}H NMR (400 MHz, CDCl3) spectra of 4-methyl-N-(2,2,3-trifluoro-1-(4-(trifluoromethyl)phenyl)but-3-en-1-yl)benzenesulfonamide, **5k**

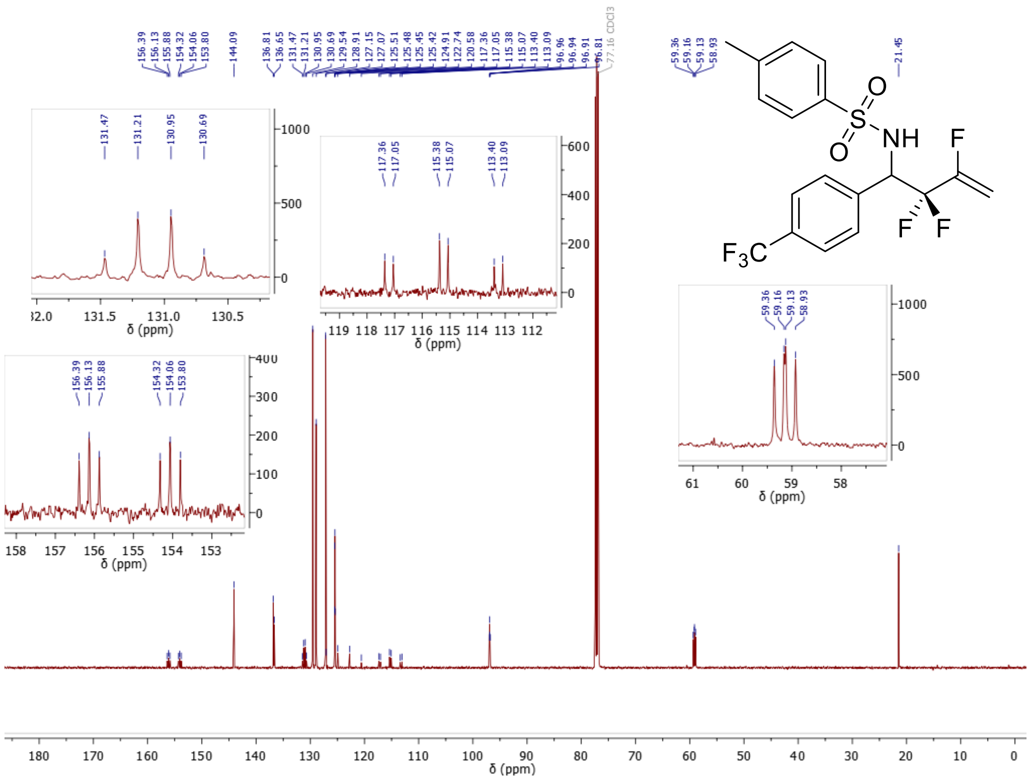

^{13}C NMR (125 MHz, CDCl3) spectra of 4-methyl-N-(2,2,3-trifluoro-1-(4-(trifluoromethyl)phenyl)but-3-en-1-yl)benzenesulfonamide, **5k**

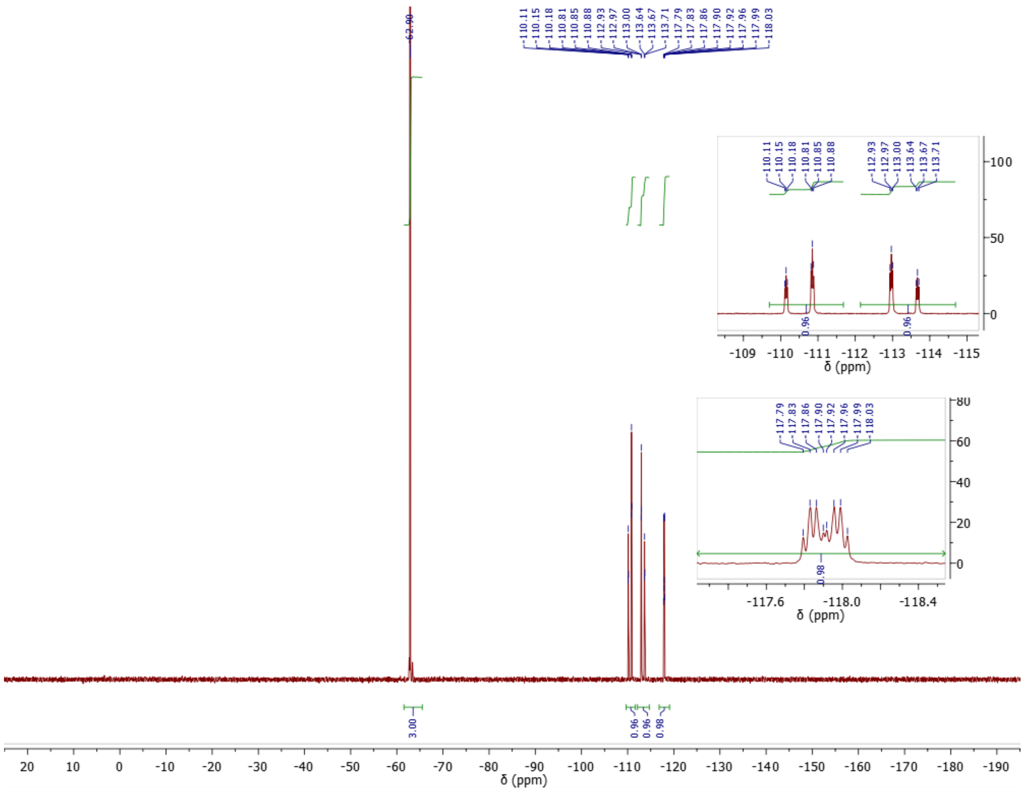

^{19}F NMR (376 MHz, $CDCl_3$) spectra of 4-methyl-N-(2,2,3-trifluoro-1-(4-(trifluoromethyl)phenyl)but-3-en-1-yl)benzenesulfonamide, **5k**

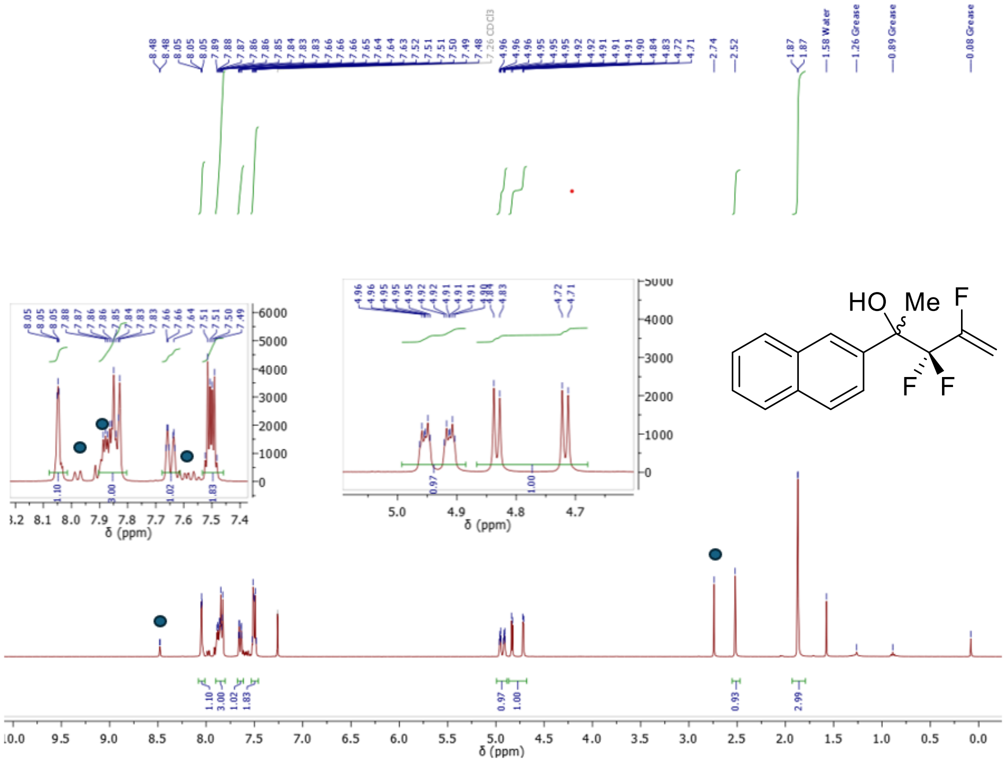

^{1}H NMR (400 MHz, $CDCl_3$) spectra of 3,3,4-trifluoro-2-(naphthalen-2-yl)pent-4-en-2-ol, **5l,** (15% starting material in blue)

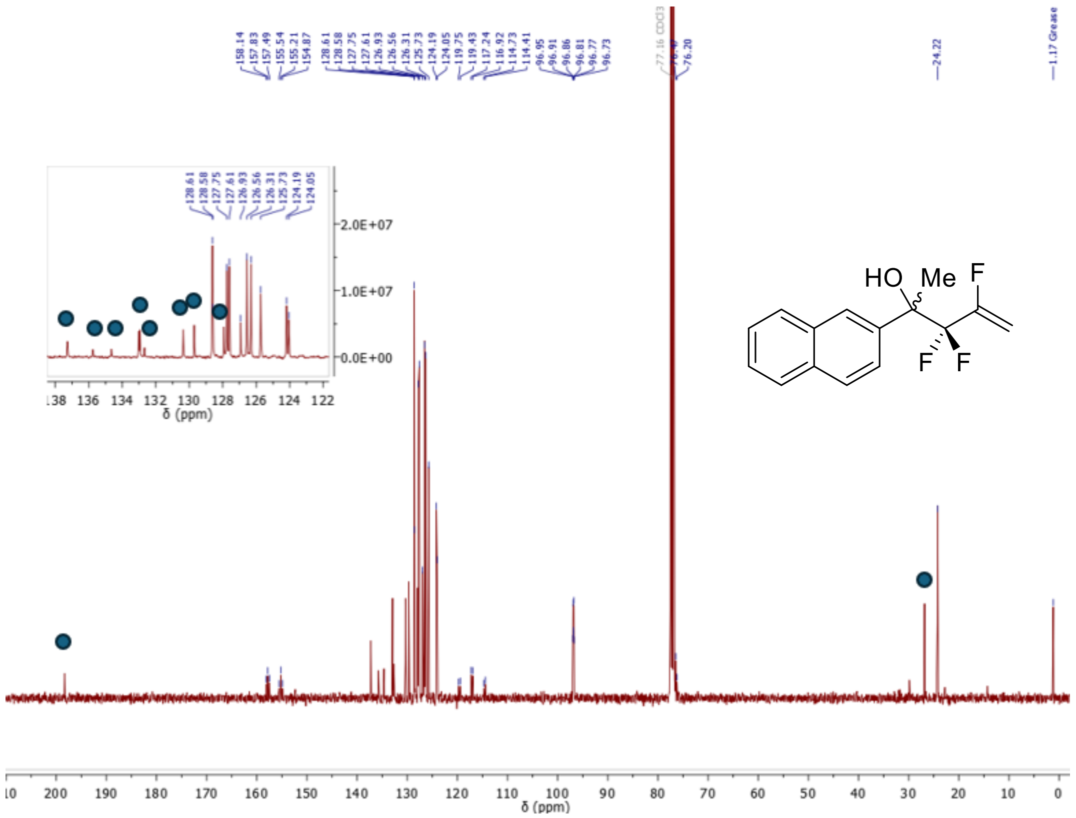

^{13}C NMR (100 MHz, $CDCl_3$) spectra of 3,3,4-trifluoro-2-(naphthalen-2-yl)pent-4-en-2-ol, **5l,** (15% starting material in blue)

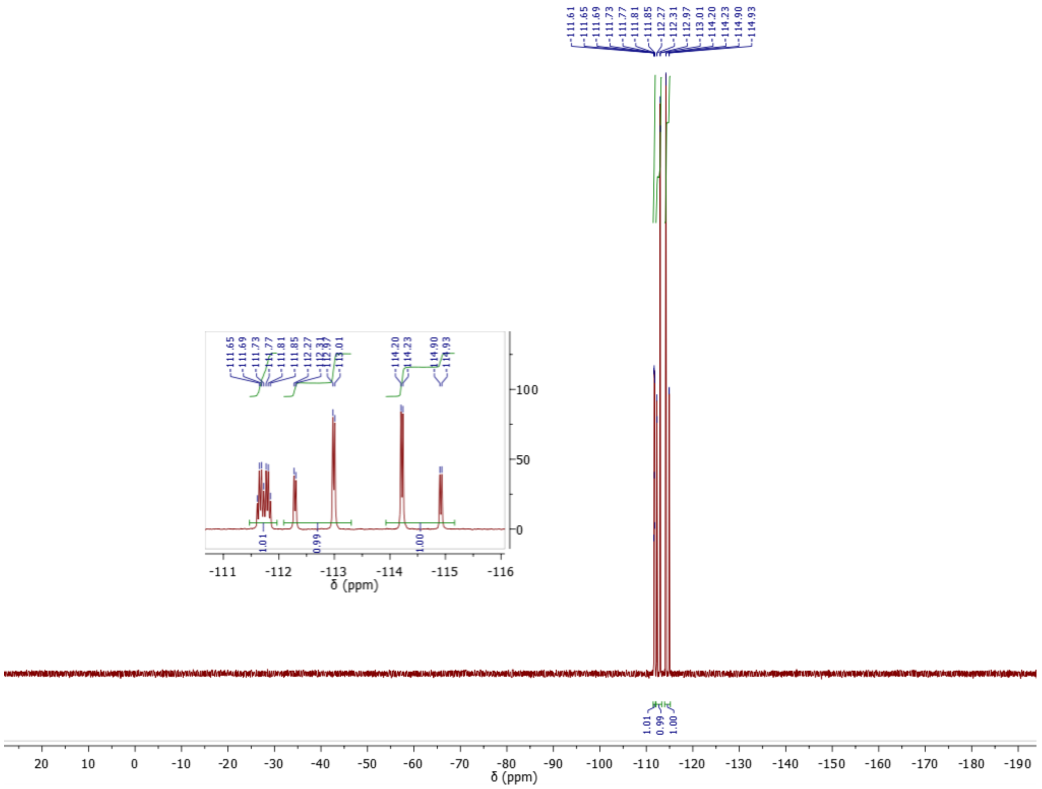

^{19}F NMR (376 MHz, $CDCl_3$) spectra of 3,3,4-trifluoro-2-(naphthalen-2-yl)pent-4-en-2-ol, **5l**

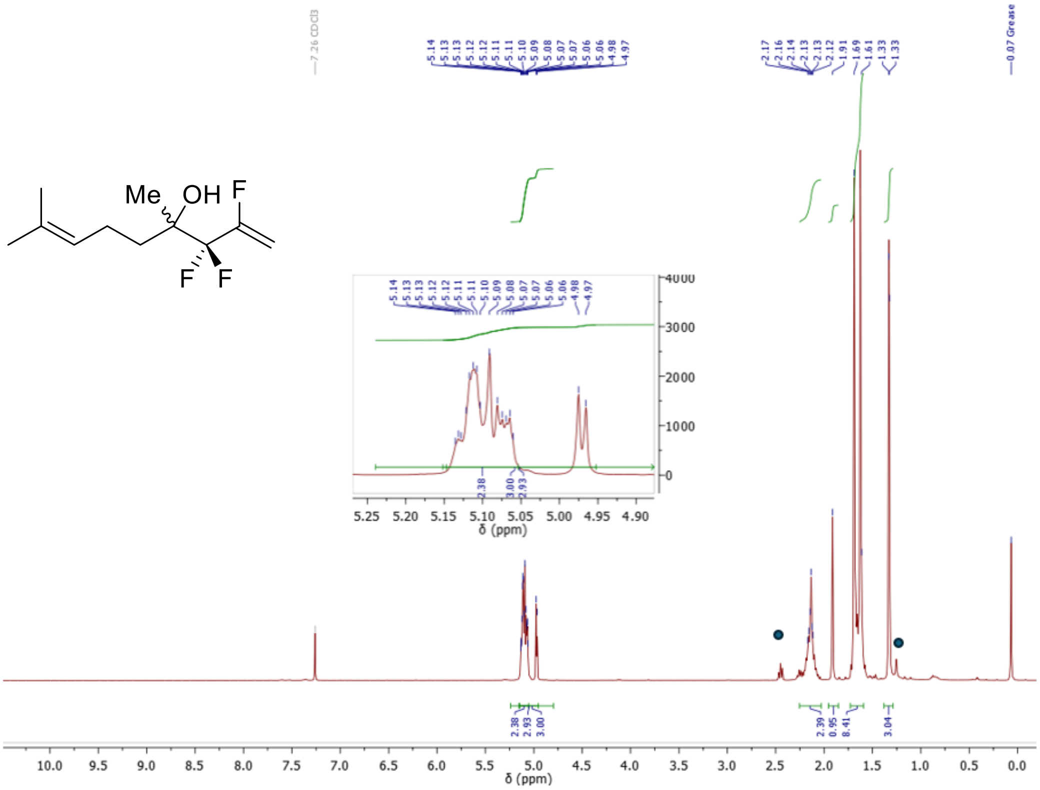

^{1}H NMR (400 MHz, $CDCl_3$) spectra of 2,3,3-trifluoro-4,8-dimethylnona-1,7-dien-4-ol, **5m,** (8% starting material in blue)

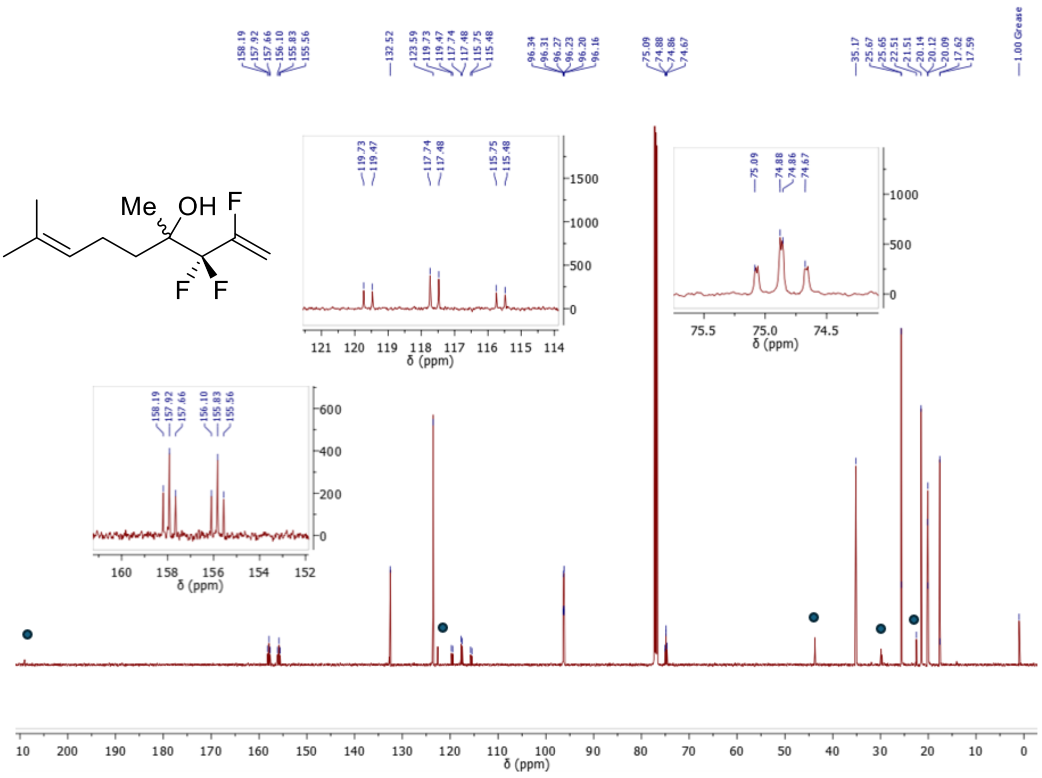

^{13}C NMR (125 MHz, $CDCl_3$) spectra of 2,3,3-trifluoro-4,8-dimethylnona-1,7-dien-4-ol, **5m,** (8% starting material in blue)

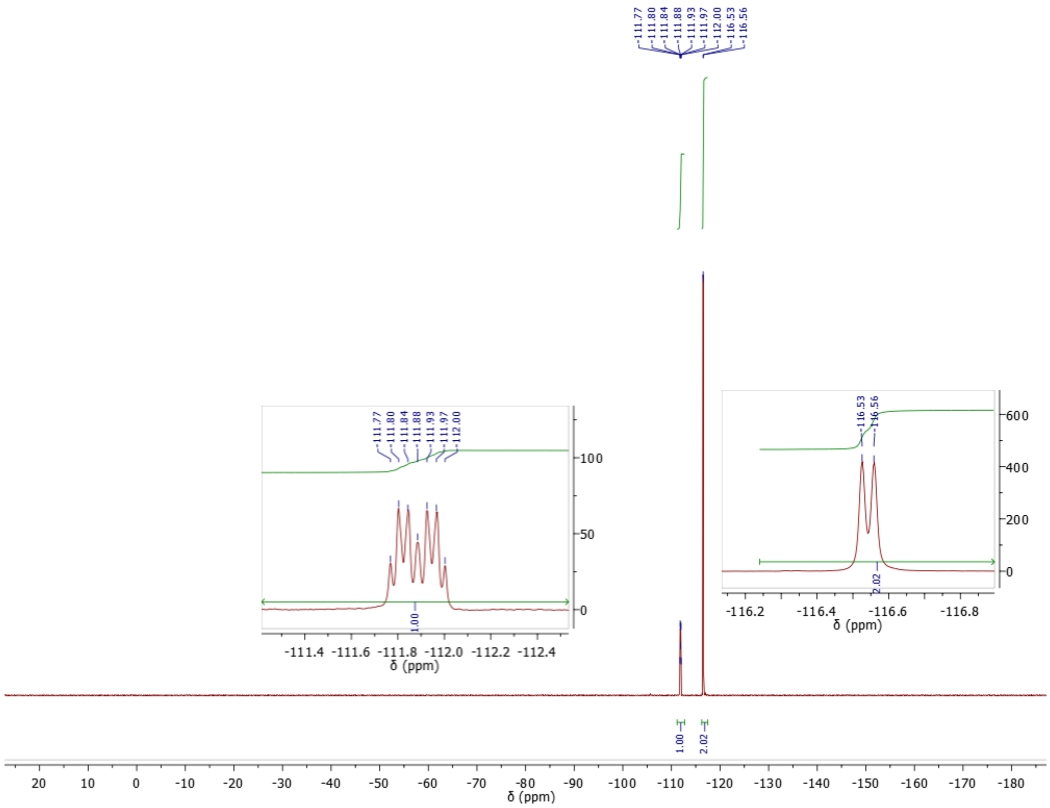

^{19}F NMR (376 MHz, $CDCl_3$) spectra of 2,3,3-trifluoro-4,8-dimethylnona-1,7-dien-4-ol, **5m**

9. References

1 V. Jurkauskas, J. P. Sadighi and S. L. Buchwald, *Org. Lett.,* 2003, **5**, 2417–2420.

2 N. P. Mankad, D. S. Laitar and J. P. Sadighi, *Organometallics*, 2004, **23**, 3369–3371.

3 D. J. Sheldon, G. Coates and M. R. Crimmin, *Chem. Commun.*, 2020, **56**, 12929–12932.

4 G. Coates, H. Y. Tan, C. Kalff, A. J. P. White and M. R. Crimmin, *Angew. Chem. Int. Ed.,* 2019, **58**, 12514–12518.

5 S. Krishnamoorthy, S. Kar, J. Kothandaraman and G. K. S. Prakash, *J. Fluor. Chem.*, 2018, **208**, 10–14.

6 S. Mizuta, N. Shibata, S. Ogawa, H. Fujimoto, S. Nakamura and T. Toru, *Chem. Commun.*, 2006, 2575.

7 Z. Deng, J.-H. Lin, J. Cai and J.-C. Xiao, *Org. Lett.,* 2016, **18**, 3206–3209.

8 Y. Yamamoto, Y. Nakazato, R. Tadano and T. Yasui, *J. Org. Chem.*, 2022, **87**, 10216–10228.

9 N. A. Phillips, G. J. Coates, A. J. P. White and M. R. Crimmin, *Chem. - Eur. J.*, 2020, **26**, 5365–5368.

10 O. V. Dolomanov, L. J. Bourhis, R. J. Gildea, J. A. K. Howard and H. Puschmann, *J. Appl. Crystallogr.*, 2009, **42**, 339–341.

11 G. M. Sheldrick, *SHELXTL v5.1*, Madison WI, 1998.

12 G. M. Sheldrick, *Acta Crystallogr. Sect. A Found. Adv.,* 2015, **71**, 3–8.
